# Stereoselective [4+2] Cycloaddition of Singlet Oxygen to Naphthalenes Controlled by Carbohydrates

**DOI:** 10.3390/molecules26040804

**Published:** 2021-02-04

**Authors:** Marcel Bauch, Werner Fudickar, Torsten Linker

**Affiliations:** Department of Chemistry, University of Potsdam, Karl-Liebknecht-Street 24-25, 14476 Golm, Germany; marcel.potsdam@gmail.com (M.B.); wfudicka@uni-potsdam.de (W.F.)

**Keywords:** singlet oxygen, photooxygenation, naphthalenes, carbohydrates, stereoselectivity, auxiliary control, [4+2] cycloaddition

## Abstract

Stereoselective reactions of singlet oxygen are of current interest. Since enantioselective photooxygenations have not been realized efficiently, auxiliary control is an attractive alternative. However, the obtained peroxides are often too labile for isolation or further transformations into enantiomerically pure products. Herein, we describe the oxidation of naphthalenes by singlet oxygen, where the face selectivity is controlled by carbohydrates for the first time. The synthesis of the precursors is easily achieved starting from naphthoquinone and a protected glucose derivative in only two steps. Photooxygenations proceed smoothly at low temperature, and we detected the corresponding endoperoxides as sole products by NMR. They are labile and can thermally react back to the parent naphthalenes and singlet oxygen. However, we could isolate and characterize two enantiomerically pure peroxides, which are sufficiently stable at room temperature. An interesting influence of substituents on the stereoselectivities of the photooxygenations has been found, ranging from 51:49 to up to 91:9 *dr* (*diastereomeric ratio*). We explain this by a hindered rotation of the carbohydrate substituents, substantiated by a combination of NOESY measurements and theoretical calculations. Finally, we could transfer the chiral information from a pure endoperoxide to an epoxide, which was isolated after cleavage of the sugar chiral auxiliary in enantiomerically pure form.

## 1. Introduction

Singlet oxygen (^1^O_2_) is an excited state of molecular oxygen and has been used for various oxidations, in biology, or medical applications [1]. Although the lifetime of this reactive species is short [2], depending on the solvent [3] or the biological environment [4], it can react with high regio- [5] and stereoselectivity [6]. It was the pioneering work of Adam, who discovered that OH groups can control the diastereoselectivity in ene reactions [7,8] or [4+2] cycloadditions of ^1^O_2_ to naphthalenes [9,10]. Later on, the influence of other functional groups on photooxygenations has been investigated [6,11], and our group described the influence of acids and esters on ene reactions [12,13]. However, the enantioselective transfer of ^1^O_2_ is still challenging, because of its short lifetime. Although the α-hydroxylation of aldehydes and ketones was realized by organocatalysis with moderate to good *ee* (*enantiomeric excess*) [14,15,16], ene reactions or cycloadditions proceed less successfully. Thus, asymmetrically modified zeolites [17], chiral salts [18], or chiral sensitizers [19] afforded only low stereoselectivities. Best results with up to 90% *ee* have been obtained in photooxygenations of 2-pyridones in the presence of chiral bicyclic lactams, which, however, had to be used in excess [20].

Another possibility to control the face-selectivity of singlet oxygen attack are chiral auxiliaries. First attempts with oxazolines gave no selectivity [21], but menthol derivatives [22] or tartrates [23] showed moderate to good induction. Best results have been obtained with oxazolidines [24], sultams [25], and especially enecarbamates [26], all developed in the group of Adam [27]. However, to the best of our knowledge, carbohydrates have not served as chiral auxiliaries for the stereoselective transfer of ^1^O_2_ until now. Only sugar-furans have been photooxygenated, but the intermediary formed peroxides rearrange thermally and cannot be isolated [28,29,30].

**Scheme 1:**
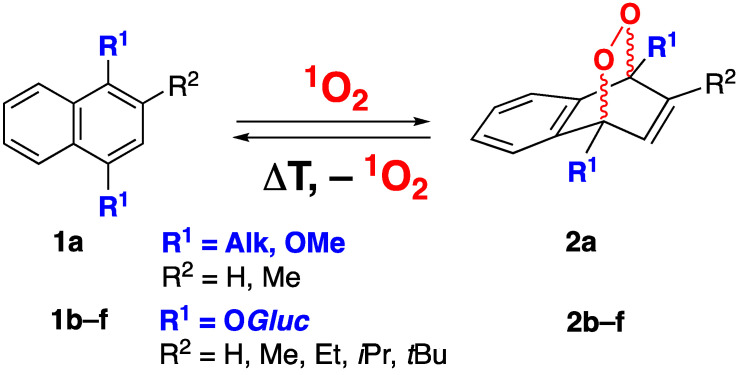
Reversible reaction of naphthalenes **1** with singlet oxygen to endoperoxides **2**.

Based on our experience in carbohydrate chemistry [31,32,33] and acene oxidations [34,35,36,37], we became interested in a combination of both research topics and in sugar-substituted naphthalenes **1** (Scheme 1). The advantage of such arenes is that they react with ^1^O_2_ by a [4+2] cycloaddition in high yields to endoperoxides (EPOs) **2**, as pointed out with simple alkyl- [38] and methoxy-substituted [39] systems **1a** (R^1^ = Alk, OMe). Furthermore, the EPOs **2a** reconvert quantitatively to the parent naphthalene **1a** under thermal conditions and under release of singlet oxygen [40,41,42]. We investigated the influence of substituents R^2^ on the kinetics of such reactions [43] and could establish an intramolecular transfer of ^1^O_2_ [44]. However, carbohydrate-substituted naphthalenes **1b**–**f** were hitherto unknown, but will generate new stereogenic centers during the oxidation (Scheme 1). Herein, we describe the synthesis of such compounds, their photooxygenation, isolation of peroxides, release of singlet oxygen, and a transformation into an enantiomerically pure epoxide.

## 2. Results and Discussion

**Scheme 2:**
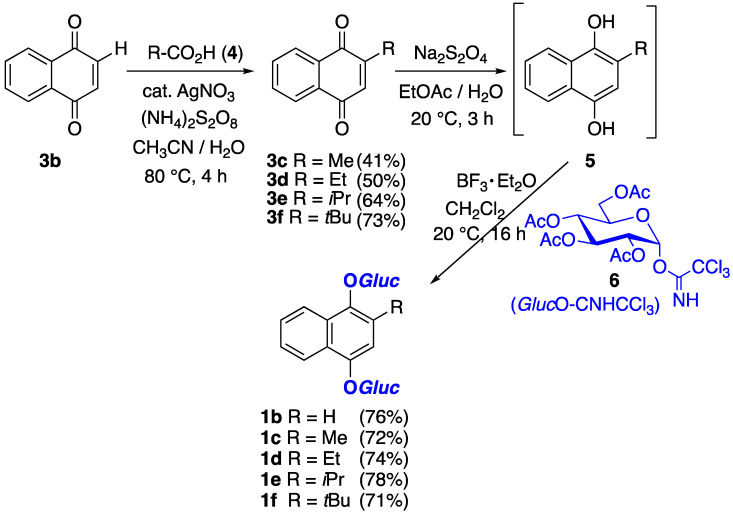
Synthesis of carbohydrate-substituted naphthalenes **1b**–**f** from naphthoquinone **3b**.

The synthesis of carbohydrate-substituted naphthalenes **1b**–**f** started from 1,4-naphthoquinone (**3b**), an inexpensive and easily available precursor. Addition of alkyl radicals, generated by decarboxylation of acids **4**, afforded substituted naphthoquinones **3c**–**f** in moderate to good yields, in analogy to literature (Scheme 2) [45]. In situ reduction with sodium dithionite [46] gave the corresponding 1,4-dihydroxynaphthalenes **5**, which were directly used for the next step without purification, because of their oxidation lability. Finally, Schmidt’s trichloroacetimidate **6** [47,48] gave best results for glycosylations, and the hitherto unknown glucose-substituted naphthalenes **1b**–**f** were isolated in good yields as single β-anomers in analytically pure form.

We started the photooxygenations with unsubstituted naphthalene **1b** (R = H). Thus, substrate **1b** (123 mg, 0.15 mmol) and sensitizer tetraphenylporphyrin (TPP, 1.0 mg) were dissolved in CD_2_Cl_2_ (3.0 mL) on an NMR scale. Oxygen was slowly bubbled through the solution, which was irradiated externally with a sodium lamp (400 W) at room temperature. The progress of the reaction was followed by TLC and NMR (300 MHz, measurements for every 60 min), however, even after 6 h, no conversion was observed (Table 1, entry 1). This can be explained by a steric hindrance of the carbohydrate substituents, or by a reversible reaction of ^1^O_2_ with naphthalenes [40,41], which is especially fast with alkoxy groups [39]. Indeed, we repeated the reaction at −70 °C and obtained full conversion after 2 h and the sole formation of one endoperoxide (EPO) **2b**, determined by NMR (500 MHz) at the same temperature (entry 2, for detailed NMR spectra see Supplementary Materials). The fact that the reaction is completely reversible was confirmed by thermolysis at different temperatures and by measurement of the reappearing naphthalene UV absorption band, which provided kinetic data. We determined a half lifetime of 2 min at 20 °C, explaining why no conversion was observed at room temperature. Furthermore, at least 68% of the oxygen released during thermolysis was in its singlet state, which we proved by trapping with tetramethylethylene, and which is in accordance to simple naphthalene EPOs [40]. However, first attempts to transfer ^1^O_2_ even enantioselectively to a prochiral acceptor failed.

After the successful oxidation of naphthalene **1b** (Table 1, entry 2), we next investigated the photooxygenations of the alkyl-substituted derivatives **1c**–**f** under the same conditions (entries 3–6). For all substrates, the conversion was complete after 2 h, and we observed EPOs **2c**–**f** as sole products in the NMR of the crude reaction mixture at low temperature (for detailed NMR spectra, see Supplementary Materials). Again, thermolysis of all EPOs proceeded completely reversible, and the parent naphthalenes **1c**–**f** were reisolated quantitatively. However, the half lifetimes differ depending on the substituents R. Thus, the *t*Bu derivative **2f** was stable at room temperature for several hours and the products could be isolated in analytically pure form by column chromatography (entry 6). The higher stability of EPOs with larger substituents is in accordance to our previous studies with achiral naphthalenes [43].

Besides the remarkable thermal stability of EPOs **2**, we found an interesting influence of the substituents on the stereoselectivity of the photooxygenations (Table 1). For naphthalene **1b,** the two arene planes are identical, and only one EPO **2b** can be formed (entry 2). However, for all other substrates **1c**–**f**, singlet oxygen can attack from two different faces, affording two diastereomers 1*S*,4*R*-**2** and 1*R*,4*S*-**2**. Indeed, naphthalenes **1c**–**e** reacted with high stereoselectivities of about 9:1 to the corresponding EPOs **2c**–**e** (entries 3–5). Surprisingly, the selectivity drops with *t*Bu-substituted naphthalene **1f** completely, and EPOs **2f** are formed almost as 1:1 mixture (Table 1, entry 6). On the other hand, the diastereomers could be easily separated by column chromatography, characterized in pure form, and used for further transformations (see below). As the glucose substituents provide the stereochemical information, their steric and electrostatic repulsion of ^1^O_2_ must be responsible for the selectivities, and thus, our concept of a carbohydrate-auxiliary-controlled photooxygenation has been realized.

**Figure 1:**
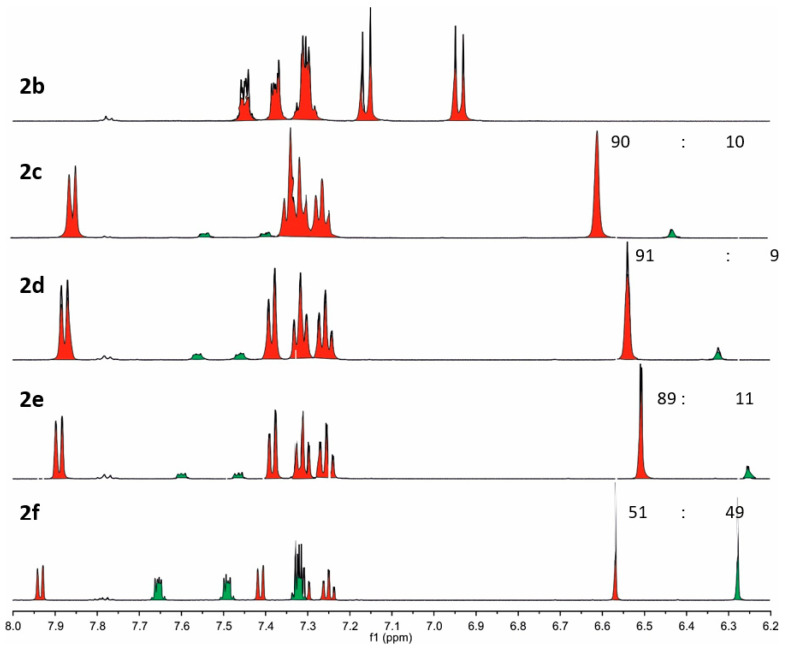
^1^H-NMR spectra (500 MHz, 6–8 ppm) of the crude photooxygenation products of carbohydrate-substituted naphthalenes **2b**–**f** (major isomer red, minor isomer green).

The thermally labile peroxides **2c**–**e** could not be separated or isolated by column chromatography, but the *dr* was unequivocally determined from the ^1^NMRs of the crude products at low temperature (Figure 1 for selected chemical shifts, see Supplementary Materials for complete spectra). Although carbohydrate signals overlap, arene and double bond protons give distinctive signals. For the single isomer **2b,** we obtained only one signal set with the protons at the double bond giving two doublets at 6.94 and 7.16 ppm. For EPOs **2c**–**f,** the two diastereomers 1*S*,4*R*-**2** and 1*R*,4*S*-**2** exhibit two different signal sets, i.e., the major isomer as marked red and the minor isomer as marked green (Figure 1b–e). Characteristic is the double doublet at 7.9 ppm for the major isomers, which is assigned as H^8^ and speaks for its close proximity to the carbohydrate ring *ortho* to the substituent R. On the other hand, this proton has a chemical shift of 7.6 ppm for the minor isomers (Figure 1, green), which is a remarkable difference.

**Figure 2:**
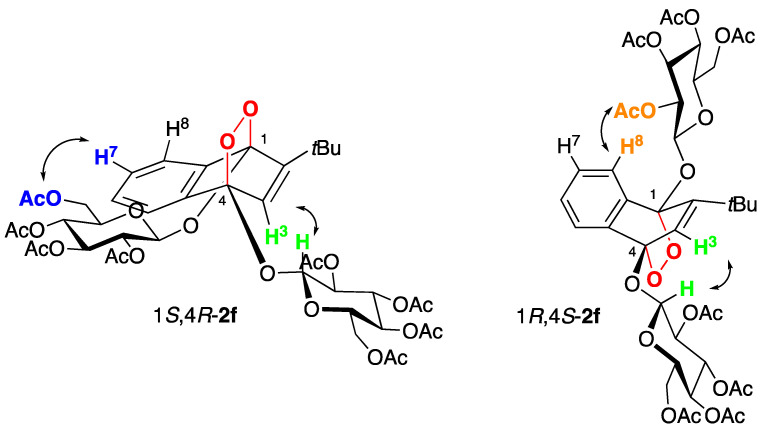
Characteristic NOE interactions in EPOs 1*S*,4*R*-**2f** and 1*R*,4*S*-**2f**.

To understand this behavior, to determine the absolute configurations, and to find a rationale for the stereoselectivities, we analyzed the two diastereomeric EPOs **2f**, which we isolated in analytically pure form, in more detail by a combination of analytical and theoretical methods. Thus, we measured their NOESY spectra (Supplementary Materials), which gave distinctive cross-peaks (Figure 2). For both isomers, the anomeric proton of the carbohydrate in the 4-position is in close proximity to H^3^ and, therefore, to the double bond (marked green). On the other hand, only EPO 1*S*,4*R*-**2f** shows an NOE between the 6-OAc group of the sugar in the 1-position and H^7^ (marked blue), which is located far away in the benzene ring. Thus, this carbohydrate is pushed away by the sterically demanding *t*Bu group under the arene ring. Additionally, this explains the downfield shift of H^8^ to 8 ppm for all main isomers 1*S*,4*R*-**2c**–**f**, resulting from the ring-oxygen of the carbohydrate. In contrast, in isomer EPO 1*R*,4*S*-**2f,** H^8^ shows a normal shift of 7.6 ppm and an NOE is only observed to the 2-OAc and not to the 6-OAc group (marked orange). In summary, we propose for the main isomers a conformation with the carbohydrate in the 1-position far away from the peroxide bridge, whereas for the minor isomer, this sugar is in close proximity to the newly formed stereocenter.

**Figure 3:**
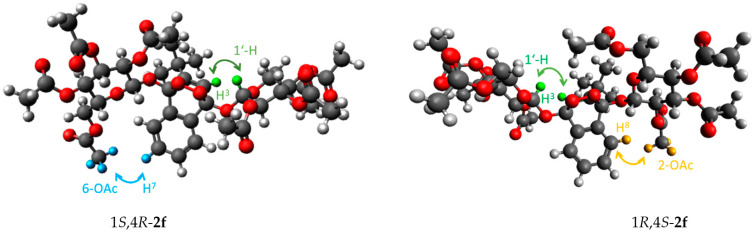
Calculated energetically minimized structures of EPOs 1*S*,4*R*-**2f** and 1*R*,4*S*-**2f**. Carbon atoms are marked black; oxygen atoms are marked red; hydrogen atoms are marked grey; blue, green, and yellow indicate the distinct NOE interactions (compare Figure 2).

To further support the observed NOESY interactions, we conducted theoretical calculations at the B3LYP/6-31G* level (for details see Supplementary Materials). Thus, structures for both diastereomeric EPOs 1*S*,4*R*-**2f** and 1*R*,4*S*-**2f** were minimized (Figure 3). Indeed, for the main isomer, we find a close proximity of the 6-OAc group of the sugar in the 1-position and H^7^, which nicely is in accordance to the NOESY measurements (Figure 2). On the other hand, in EPO 1*R*,4*S*-**2f,** both carbohydrates are located in the plane of the peroxide bridge, again in good correlation to the NMR spectra.

**Figure 4:**
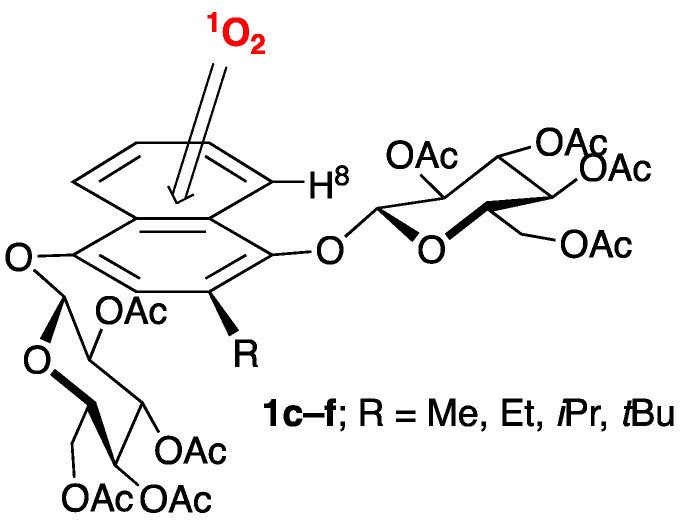
Proposed conformations of naphthalenes **1c**–**f** and preferred attack of ^1^O_2_.

Our structural assignment of the configurations and conformations of EPOs **2** might give an explanation for the observed stereoselectivities and influence of substituents as well (Figure 4). Thus, for starting materials **1c**–**f,** the carbohydrate in the 1-position is pushed away by the substituent R, resulting in a downfield shift of H^8^ (see NMR data in the section Materials and Methods). The second carbohydrate in the 4-position is much closer to the reacting naphthalene ring and, therefore, shields the *si* face. Thus, singlet oxygen has to attack from the opposite face, passing by the R group. Although the discussion of rotamers for the explanation of stereoselectivities is a bit speculative, our mechanistic proposal nicely fits to the observed influence of substituents. For naphthalene **1b** (R = H), no selectivity can result, but the photooxygenation of isomers **1c**–**e** (R = Me, Et, *i*Pr) provide good stereoselectivities (see Table 1). Only for starting material **1f** (R = *t*Bu), the selectivity drops remarkably, because ^1^O_2_ has to pass by this sterically demanding group, and therefore, both faces are shielded equally. The fact that the *t*Bu (**1f**) and *i*Pr (**1e**) derivatives react with such different selectivity is surprising, because their steric substituent constants are in the same range [49]. We explain this again by hindered rotations due to interactions with the carbohydrates. Thus, for R = *i*Pr, the two methyl groups may point away from the sugar in the 1-position, whereas for R = *t*Bu, one methyl group shields the upper plane (Figure 4). The fact that a *t*Bu group gives remarkably different stereoselectivities than an *i*Pr group was found in other auxiliary-controlled reactions as well [50].

**Scheme 3:**
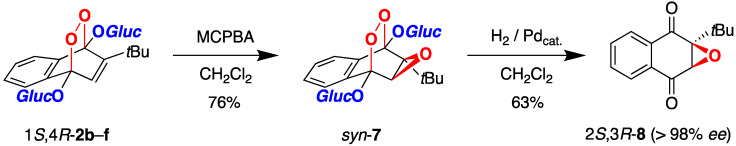
Transformation of EPO 1*S*,4*R*-**2f** into enantiomerically pure epoxide 2*S*,3*R*-**8**.

Finally, because our structural assignment was hitherto only based on NOESY measurements and calculations, we wanted to determine the absolute configurations unequivocally. Thus, we selected EPO 1*S*,4*R*-**2f**, which we could isolate in analytically pure form, which may have the same configuration as all main isomers, and which is stable enough for further transformations. Epoxidation with *m*-chloroperoxybenzoic acid (MCPBA) in dichloromethane afforded a single diastereomer *syn*-**7** as shown by the NMR of the crude product. This is in accordance to literature-known epoxidations of simple naphthalene EPOs [51] and can be explained by steric interactions and hydrogen bonding to the peroxide bridge. The epoxide *syn*-**7** was isolated by column chromatography in 76% yield in analytically pure form (Scheme 3). Finally, the chiral carbohydrate auxiliaries could be removed by catalytic hydrogenation of the O–O bond, and naphthoquinone epoxide 2*S*,3*R*-**8** was isolated in 63% yield, besides some decomposition products. Now, we could determine its absolute configuration unequivocally by comparison of the optical rotation with literature-known epoxide 2*R*,3*S*-**8**, which was synthesized from chiral hydroperoxides in 82% *ee* [52].

**Figure 5:**
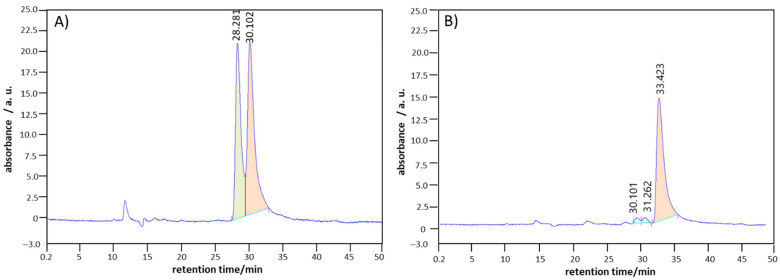
HPLC separation of epoxide **8** on chiral phase. (**A**) racemate **8** and (**B**) epoxide 2*S*,3*R*-**8**. 2*R*,3*S* isomer is marked green; 2*S*,3*R* isomer is marked red.

The fact that our epoxide is enantiomerically pure was confirmed by HPLC on a chiral phase (Figure 5, Section 3). First, we synthesized racemic epoxide **8** by direct oxidation of naphthoquinone **3f** in analogy to literature [53]. This racemate gave two peaks, separated by 2 min retention time (A), and epoxide 2*S*,3*R*-**8** gave a single peak (B). Therefore, we could use carbohydrates as chiral auxiliaries to control the photooxygenation of naphthalenes, isolate endoperoxides in pure form, and transfer the chirality to new stereogenic centers under cleavage of the auxiliary, obtaining an enantiomerically pure product.

## 3. Materials and Methods

### 3.1. Chemicals and Instrumentation

Melting points were determined using a Mel-Temp from Electrothermal. TLC was performed using TLC Silica gel 60 F254 aluminum sheets from Merck. ^1^H-NMR and ^13^C-NMR spectra were measured using a Bruker Avance 300 (300 MHz, 75 MHz) (Rheinstetten, Germany), a Bruker NEO 500 (500 MHz, 125 MHz) (Rheinstetten, Germany), or a Bruker Avance 600 (600 MHz, 150 MHz) NMR spectrometer (Rheinstetten, Germany). The solvents (CDCl_3_ or CD_2_Cl_2_) were used as standard for calibrating chemical shifts. Signals were assigned by two-dimensional methods (HSQC). IR spectra were recorded in KBr pellets using a Nicolet Avatar 370 FT-IR spectrometer (Madison, WI, USA) from Thermo Electron Corporation. Optical rotations were measured on a JASCO P-1020 digital polarimeter (Tokio, Japan) at 589 nm. Elemental analysis was performed on a Vario EL III elemental analyzer (Elementar, Langenselbold, Germany). HRMS spectra were measured at a GC-MS Trace DSX II spectrometer (Dreieich, Germany). UV spectra were recorded with an Analytik Jena Specord S600 spectrometer (Analytik Jena, Jena, Germany). HPLC was performed with a Chrom Tech, Chiral-AGP column (100 mm × 3.00 mm, 5 μm), eluent hexane/ethyl acetate 10:1. All starting materials were used as purchased without further purification. Trichloroacetimidate *gluco*-**6** was synthesized according to the literature [54].

### 3.2. Synthesis of Naphthoquinones ***3***

According to the literature [45], 1,4-naphthoquinone **3b** (3.95 g, 25 mmol) and the corresponding carboxylic acid **4** (50 mmol, 2 equiv.) were dissolved in a 1:1 mixture of dichloromethane and acetonitrile (100 mL). This mixture was added to a solution of silver nitrate (425 mg, 2.5 mmol) and ammonium persulfate (11.4 g, 50 mmol) in water (100 mL) at room temperature. The mixture was heated under reflux for 4 h, cooled to room temperature, and diluted with dichloromethane (100 mL), and the organic phases were separated. The aqueous phase was extracted with dichloromethane (3 × 50 mL), and the combined organic layers were washed with brine (300 mL) and dried over magnesium sulfate. The solvent was removed in vacuo, and the crude products were purified by column chromatography (PE/EtOAc 10:1) to obtain the naphthoquinones **3c**–**f** in analytically pure form.

*2-Methyl-1,4-naphthoquinone***3c.** Obtained as a yellow solid (1.76 g, 41%). m.p. = 106–108 °C. *R_f_* = 0.23 (PE/EtOAc 10:1). ^1^H-NMR (500 MHz, CD_2_Cl_2_): δ = 2.16 (d, *J* = 1.6 Hz, 3H; C*H_3_*), 6.81 (q, *J* = 1.6 Hz, 1H; 3-H), 6.77 (t, *J* = 1.6 Hz, 1H; 3-H), 7.70–7.74 (m, 2H; 7-H, 6-H), 7.99–8.02 (m, 1H; H-5), 8.03–8.07 ppm (m, 1H; 8-H). ^13^C-NMR (125 MHz, CD_2_Cl_2_): δ= 16.5 (q; *C*H_3_), 126.2 (d; C-5), 126.6 (d; C-8), 132.6 (s; C-8a), 132.6 (s; C-4a), 133.9 (d; C-7), 134.0 (d; C-6), 135.9 (d; C-3), 148.5 (s; C-2), 185.2 (s; C-4), 185.8 ppm (s; C-1); IR (Film): v = 3656 (w), 3292 (w), 2956 (m), 2920 (m), 1621 (s), 1588 (m), 1464 (m), 1329 (m), 1300 (s), 1245 (m), 1143 (m), 1061 cm^−1^ (m). HRMS (GC-TOF): *m*/*z* Calcd for C_11_H_8_O_2_ [M^+^]: 172.0524; Found: 172.0533. Anal. Calcd for C_11_H_8_O_2_ (172.18): C 76.73, H 4.68; Found: C 77.01, H 4.73.

*2-Ethyl-1,4-naphthoquinone***3d.** Obtained as a yellow solid (2.35 g, 50%). m.p. = 83–84 °C. *R_f_* = 0.27 (PE/EtOAc 10:1). ^1^H-NMR (600 MHz, CD_2_Cl_2_): δ = 1.19 (t, *J* = 7.4 Hz, 3H; CH_3_), 2.59 (dq, *J* = 7.4, 1.6 Hz, 2H; CH_2_), 6.77 (t, *J* = 1.6 Hz, 1H; 3-H), 7.72–7.75 (m, 2H; 7-H, 6-H), 8.01–8.03 (m, 1H; H-5), 8.06–8.09 ppm (m, 1H; 8-H). ^13^C-NMR (150 MHz, CD_2_Cl_2_): δ = 12.0 (q; *C*H_3_), 23.0 (t; *C*H_2_), 126.2 (d; C-5), 126.7 (d; C-8), 132.5 (s; C-8a), 132.8 (s; C-4a), 133.9 (d; C-7), 134.0 (d; C-6), 134.3 (d; C-3), 153.5 (s; C-2), 185.5 (s; C-4), 185.5 ppm (s; C-1). IR (Film): v = 3668 (w), 3312 (w), 2973 (m), 2906 (m), 1661 (s), 1590 (m), 1458 (m), 1385 (m), 1327 (m), 1296 (s), 1250 (m), 1143 (m), 1057 cm^−1^ (m). HRMS (GC-TOF): *m*/*z* Calcd for C_12_H_10_O_2_ [M^+^]: 186.0681; Found: 186.0687. Anal. Calcd for C_12_H_10_O_2_ (186.21): C 77.40, H 5.41; Found: C 77.40, H 5.71.

*2-iPropyl-1,4-naphthoquinone***3e.** Obtained as a yellow solid (3.21 g, 64%). m.p. = 43–44 °C. *R_f_* = 0.34 (PE/EtOAc 10:1). ^1^H-NMR (600 MHz, CD_2_Cl_2_): δ = 1.19 (2d, *J* = 6.9 Hz, 6H; C*H_3_*), 3.22 (dsept, *J* = 6.9, 1.2 Hz, 1H; C*H*), 6.76 (d, *J* = 1.2 Hz, 1H; 3-H), 7.72–7.76 (m, 2H; 6-H, 7-H), 8.01–8.04 (m, 1H; H-5), 8.07–8.10 ppm (m, 1H; 8-H). ^13^C-NMR (150 MHz, CD_2_Cl_2_): δ = 21.7 (2q; *C*H_3_), 27.4 (d; *C*H), 126.1 (d; C-5), 126.8 (d; C-8), 132.3 (s; C-4a), 133.0 (s; C-8a), 133.0 (d; C-3), 133.9 (d; C-7), 134.0 (d; C-6), 157.5 (s; C-2), 185.1 (s; C-1), 185.8 ppm (s; C-4). IR (Film): v = 3660 (w), 3315 (w), 3071 (w), 2967 (m), 2873 (m), 1660 (s), 1594 (m), 1463 (w), 1379 (w), 1328 (m), 1299 (s), 1251 (s), 1130 (w), 1074 cm^−1^ (m). HRMS (GC-TOF): *m*/*z* Calcd for C_13_H_12_O_2_ [M^+^]: 200.0837; Found: 200.0832. Anal. Calcd for C_13_H_12_O_2_ (200.23): C 77.98, H 6.04; Found: C 77.84, H 5.94.

*2-tButyl-1,4-naphthoquinone***3f.** Obtained as a yellow solid (3.89 g, 73%). m.p. = 66–67 °C. *R_f_* = 0.42 (PE/EtOAc 10:1). ^1^H-NMR (600 MHz, CD_2_Cl_2_): δ = 1.36 (s, 9H; t-Bu), 6.83 (s, 1H; 3-H), 7.71 (ddd, *J* = 8.9, 7.4, 1.5 Hz, 1H; 6-H), 7.74 (ddd, *J* = 8.9, 7.4, 1.6 Hz, 1H; H-7), 8.00 (ddd, *J* = 7.4, 1.6, 0.6 Hz, 1H; H-5), 8.07 ppm (ddd, *J* = 7.4, 1.5, 0.6 Hz, 1H; 8-H). ^13^C-NMR (150 MHz, CD_2_Cl_2_): δ = 29.5 (q; t-Bu), 36.0 (s; t-Bu), 125.7 (d; C-5), 127.0 (d; C-8), 131.9 (s; C-4a), 133.6 (d; C-6), 134.0 (s; C-8a), 134.1 (d; C-7), 134.3 (d; C-3), 158.5 (s; C-2), 185.3 (s; C-1), 186.1 ppm (s; C-4). IR (Film): v = 3306 (w), 3071 (w), 2963 (m), 2910 (m), 2874 (m), 1792 (w), 1655 (s), 1594 (s), 1485 (m), 1460 (m), 1362 (m), 1331 (s), 1308 (s), 1249 (s), 1202 (m), 1127 cm^−1^ (s). HRMS (GC-TOF): *m*/*z* Calcd for C_14_H_14_O_2_ [M^+^]: 214.0994; Found: 214.0988. Anal. Calcd for C_14_H_14_O_2_ (214.26): C 78.48, H 6.59; Found: C 78.56, H 6.68.

### 3.3. Synthesis of Carbohydrate-Substituted Naphthalenes ***1***

In short, 1,4-Naphthoquinone **3** (5 mmol) was dissolved in EtOAc (15 mL) and an aqueous solution of sodium dithionite (2.61 g, 15 mmol, 15 mL) was added under nitrogen atmosphere at room temperature. After stirring for 3 h, the mixture was diluted with a saturated solution of ammonium chloride (15 mL) and extracted with EtOAc (3 × 25 mL). The combined organic layers were washed with water (50 mL) and brine (50 mL) and dried over magnesium sulfate. The solvent was removed in vacuo, and the crude 1,4-dihydroxynaphthalene **5** was directly used for the next step.

The crude 1,4-dihydroxynapthalene **5** was dissolved in dry dichloromethane (50 mL), molecular sieves (4 Å), and a solution of trichloroacetimidate **6** (4.93 g, 10 mmol) in dry dichloromethane (50 mL) was added at room temperature. The mixture was cooled to −15 °C, and a solution of BF_3_·Et_2_O (0.13 mL, 1 mmol) in dry dichloromethane (5 mL) was added within 30 min. After stirring for 16 h at room temperature, the mixture was filtered over celite; washed with saturated solution of NaHCO_3_ (50 mL), water (50 mL), and brine (50 mL); and dried over magnesium sulfate. The solvent was removed in vacuo, and the crude products were purified by column chromatography (hexanes/EtOAc 1:1) to obtain the carbohydrate-substituted naphthalenes **1** in analytically pure form.

*1,4-Bis-(1-O-2,3,4,6-tetra-O-acetyl-β-d-glucopyranosyl)-naphthalene***1b.** Obtained as a colorless solid (2.923 g, 76%). m.p. = 169–171 °C. *R_f_* = 0.18 (PE/EtOAc 1:1). [α${]}_{D}^{24}$ = −67 (*c* 1.00, CHCl_3_). ^1^H-NMR (600 MHz, CD_2_Cl_2_): δ = 2.04 (2s, 6H; 3-OAc), 2.04 (2s, 6H; 4-OAc), 2.05 (2s, 6H; 2-OAc), 2.06 (2s, 6H; 6-OAc), 3.94 (2ddd, *J* = 10.0, 5.0, 2.5 Hz, 2H; 5-H), 4.20 (2dd, *J* = 12.3, 2.5 Hz, 2H; 6_a_-H), 4.32 (2dd, *J* = 12.3, 5.0 Hz, 2H; 6_b_-H), 5.21 (2dd, *J* = 10.0, 9.4 Hz, 2H; 4-H), 5.21 (2d, *J* = 7.9 Hz, 2H; 1-H), 5.36 (2dd, *J* = 9.7, 9.4 Hz, 2H; 3-H), 5.43 (2dd, *J* = 9.7, 7.9 Hz, 2H; 2-H), 7.02 (2s, 2H; 2-ArH, 3-ArH), 7.53–7.56 (m, 2H; 6-ArH, 7-ArH), 8.02–8.05 ppm (m, 2H; 5-ArH, 8-ArH). ^13^C-NMR (150 MHz, CD_2_Cl_2_): δ = 20.8 (4q; OAc-2, OAc-3), 20.9 (2q; OAc-6), 20.9 (2q; OAc-4), 62.2 (2t; C-6), 68.7 (2d; C-4), 71.4 (2d; C-2), 72.5 (2d; C-5), 72.8 (2d; C-3), 100.3 (2d; C-1), 109.7 (2d; ArC-2, ArC-3), 121.8 (2d; ArC-5, ArC-8), 126.9 (2s; ArC-4a, ArC-8a), 127.1 (2d; ArC-6, ArC-7), 149.0 (2s; ArC-1, ArC-4), 169.8 (2s; OAc-4), 169.9 (2s; OAc-2), 170.3 (2s; OAc-3), 170.7 ppm (2s; OAc-6). IR (Film): ν = 3024 (w), 2960 (w), 1746 (s), 1599 (w), 1371 (m), 1212 (s), 1122 (m), 1035 cm^−1^ (s). HRMS (ESI-Q-TOF): *m*/*z* Calcd for C_38_H_44_NaO_20_ [M + Na^+^]: 843.2324; Found: 843.2296. Anal. Calcd for C_38_H_44_O_20_ (820.75): C 55.61, H 5.40; Found: C 55.61, H 5.55.

*2-Methyl-1,4-bis-(1-O-2,3,4,6-tetra-O-acetyl-β-d-glucopyranosyl)-naphthalene***1c**. Obtained as a colorless solid (2.994 g, 72%). m.p. = 216–217 °C. *R_f_* = 0.17 (PE/EtOAc 1:1). [α${]}_{D}^{24}$ = −34 (*c* 1.00, CHCl_3_). ^1^H-NMR (600 MHz, CD_2_Cl_2_): δ = 1.88 (s, 3H; 6-OAc), 1.99 (s, 3H; 4-OAc), 2.03 (s, 3H; 2′-OAc), 2.03 (s, 3H; 3-OAc), 2.04 (s, 3H; 3′-OAc), 2.05 (s, 3H; 4′-OAc), 2.08 (s, 3H; 6′-OAc), 2.18 (s, 3H; 2-OAc), 2.46 (s, 3H; C*H_3_*), 3.56 (ddd, *J* = 10.0, 5.1, 2.6 Hz, 1H; 5-H), 3.98 (dd, *J* = 12.2, 2.6 Hz, 1H; 6a-H), 4.01 (ddd, *J* = 10.1, 5.8, 2.5 Hz, 1H, 5′-H), 4.17 (dd, *J* = 12.2, 5.1 Hz, 1H; 6b-H), 4.22 (dd, *J* = 12.3, 2.5 Hz, 1H; 6a′-H), 4.28 (dd, *J* = 12.3, 5.8 Hz, 1H; 6b′-H), 5.05 (d, *J* = 8.1 Hz, 1H; 1-H), 5.18 (dd, *J* = 10.0, 9.4 Hz, 1H; 4-H), 5.18 (dd, *J* = 10.1, 9.3 Hz, 1H; 4′-H), 5.26 (d, *J* = 7.8 Hz, 1H; 1′-H), 5.30 (dd, *J* = 9.8, 9.4 Hz, 1H; 3-H), 5.38 (dd, *J* = 9.8, 9.3 Hz, 1H; 3′-H), 5.44 (dd, *J* = 9.8, 8.1 Hz, 1H; 2-H), 5.45 (dd, *J* = 9.8, 7.8 Hz, 1H; 2′-H), 6.94 (s, 1H; 3-ArH), 7.44 (ddd, *J* = 8.4, 6.8, 1.2 Hz, 1H; 6-ArH), 7.52 (ddd, *J* = 8.6, 6.8, 1.3 Hz, 1H; 7-ArH), 8.00 (ddd, *J* = 8.4, 1.3, 0.6 Hz, 1H; 5-ArH), 8.12 ppm (ddd, *J* = 8.6, 1.2, 0.6 Hz, 1H; 8-ArH). ^13^C-NMR (150 MHz, CD_2_Cl_2_): δ = 17.5 (q; *C*H_3_), 20.6 (q; OAc-6), 20.7 (q; OAc-4), 20.8 (q; OAc-2′), 20.8 (2q; OAc-2, OAc-3′), 20.9 (2q; OAc-4′, OAc-6′), 21.0 (q; OAc-2), 62.0 (t; C-6), 62.6 (t; C-6′), 68.8 (d; C-4), 68.9 (d; C-4′), 71.4 (d; C-2′), 72.1 (C-5), 72.2 (d; C-2), 72.5 (d; C-5′), 72.7 (d; C-3′), 73.1 (d; C-3), 99.9 (d; C-1′), 102.4 (d; C-1), 112.6 (d; ArC-3), 121.9 (d; ArC-5), 122.2 (d; ArC-8), 125.5 (s; ArC-4a), 125.8 (s; ArC-6), 127.1 (d; ArC-7), 128.2 (s; ArC-8a), 129.0 (s; ArC-2), 144.8 (s; ArC-1), 150.0 (s; ArC-4), 169.6 (s; OAc-2), 169.8 (s; OAc-2′), 169.8 (s; OAc-4), 169.8 (s; OAc-4), 170.3 (s; OAc-3′), 170.4 (s; OAc-3), 170.5 (s; OAc-6), 170.7 ppm (s; OAc-6′). IR (Film): v = 2968 (m), 2899 (w), 1742 (s), 1367 (m), 1220 (s), 1170 (m), 1123 (m), 1038 cm^−1^ (s). HRMS (ESI-Q-TOF): *m*/*z* Calcd for C_39_H_46_NaO_20_ [M + Na^+^]: 857.2475; Found: 857.2477. Anal. Calcd for C_39_H_46_O_20_ (834.78): C 56.11, H 5.55; Found: C 56.03, H 5.61.

*2-Ethyl-1,4-bis-(1-O-2,3,4,6-tetra-O-acetyl-β-d-glucopyranosyl)-naphthalene***1d.** Obtained as a colorless solid (3.155 g, 74%). m.p. = 218–219 °C. *R_f_* = 0.22 (PE/EtOAc 1:1). [α${]}_{D}^{23}$ = −28 (*c* 1.01, CHCl_3_). ^1^H-NMR (600 MHz, CD_2_Cl_2_): δ = 1.25 (t, *J* = 7.6 Hz, 3H; C*H_3_*), 1.89 (s, 3H; 6-OAc), 1.99 (s, 3H; 4-OAc), 2.03 (s, 3H; 2′-OAc), 2.03 (s, 3H; 3-OAc), 2.04 (s, 3H; 3′-OAc), 2.05 (s, 3H; 4′-OAc), 2.07 (s, 3H; 6′-OAc), 2.19 (s, 3H; 2-OAc), 2.88 (q, *J* = 7.6 Hz, 2H; C*H_2_*), 3.55 (ddd, *J* = 10.0, 5.0, 2.6 Hz, 1H; 5-H), 3.94 (dd, *J* = 12.2, 2.6 Hz, 1H; 6a-H), 4.00 (ddd, *J* = 10.1, 5.7, 2.5 Hz, 1H, 5′-H), 4.15 (dd, *J* = 12.2, 5.0 Hz, 1H; 6b-H), 4.22 (dd, *J* = 12.3, 2.5 Hz, 1H; 6a′-H), 4.26 (dd, *J* = 12.3, 5.7 Hz, 1H; 6b′-H), 5.04 (d, *J* = 8.1 Hz, 1H; 1-H), 5.17 (dd, *J* = 10.0, 9.4 Hz, 1H; 4-H), 5.18 (dd, *J* = 10.1, 9.3 Hz, 1H; 4′-H), 5.26 (d, *J* = 7.8 Hz, 1H; 1′-H), 5.29 (dd, *J* = 9.8, 9.4 Hz, 1H; 3-H), 5.37 (dd, *J* = 9.8, 9.3 Hz, 1H; 3′-H), 5.44 (dd, *J* = 9.8, 8.1 Hz, 1H; 2-H), 5.44 (dd, *J* = 9.8, 7.8 Hz, 1H; 2′-H), 6.98 (s, 1H; 3-ArH), 7.46 (ddd, *J* = 8.4, 6.8, 1.3 Hz, 1H; 6-ArH), 7.52 (ddd, *J* = 8.5, 6.8, 1.4 Hz, 1H; 7-ArH), 8.00 (ddd, *J* = 8.4, 1.4, 0.6 Hz, 1H; 5-ArH), 8.14 ppm (ddd, *J* = 8.5, 1.3, 0.6 Hz, 1H; 8-ArH). ^13^C-NMR (150 MHz, CD_2_Cl_2_): δ = 15.1 (q; *C*H_3_), 20.7 (q; OAc-6), 20.8 (q; OAc-4), 20.8 (q; OAc-2′), 20.8 (2q; OAc-2, OAc-3′), 20.8 (q; OAc-4′), 20.9 (q; OAc-6′), 21.0 (q; OAc-2), 23.5 (t; *C*H_2_), 62.0 (t; C-6), 62.6 (t; C-6′), 68.8 (d; C-4), 68.8 (d; C-4′), 71.4 (d; C-2′), 72.2 (C-5), 72.2 (d; C-2), 72.7 (d; C-5′), 72.7 (d; C-3′), 73.2 (d; C-3), 100.0 (d; C-1′), 102.4 (d; C-1), 110.9 (d; ArC-3), 121.9 (d; ArC-5), 122.5 (d; ArC-8), 125.6 (s; ArC-4a), 125.9 (s; ArC-6), 127.1 (d; ArC-7), 129.0 (s; ArC-8a), 134.2 (s; ArC-2), 144.0 (s; ArC-1), 150.4 (s; ArC-4), 169.6 (s; OAc-2′), 169.8 (s; OAc-2), 169.8 (s; OAc-4′), 169.8 (s; OAc-4), 170.3 (s; OAc-3′), 170.4 (s; OAc-3), 170.6 (s; OAc-6), 170.8 ppm (s; OAc-6′). IR (Film): v = 3667 (w), 3482 (w), 2970 (m), 2901 (m), 1746 (s), 1365 (m), 1211 (s), 1170 (m), 1034 cm^−1^ (s). HRMS (ESI-Q-TOF): *m*/*z* Calcd for C_40_H_48_NaO_20_ [M + Na^+^]: 871.2631; Found: 871.2634. Anal. Calcd for C_40_H_48_O_20_ (848.80): C 56.60, H 5.70; Found: C 56.85, H 5.83.

*2-iPropyl-1,4-bis-(1-O-2,3,4,6-tetra-O-acetyl-β-d-glucopyranosyl)-naphthalene***1e.** Obtained as a colorless solid (3.375 g, 74%). m.p. = 219–220 °C. *R_f_* = 0.27 (PE/EtOAc 1:1). [α${]}_{D}^{23}$ = −26 (*c* 1.01, CHCl_3_). ^1^H-NMR (600 MHz, CD_2_Cl_2_): δ = 1.23 (d, *J* = 6.9 Hz, 3H; C*H_3_*), 1.24 (d, *J* = 6.9 Hz, 3H; C*H_3_*), 1.91 (s, 3H; 6-OAc), 1.99 (s, 3H; 4-OAc), 2.03 (s, 3H; 3-OAc), 2.04 (2s, 6H; 2′-OAc, 3′-OAc), 2.05 (s, 3H; 4′-OAc), 2.06 (s, 3H; 6′-OAc), 2.20 (s, 3H; 2-OAc), 3.55 (ddd, *J* = 10.0, 4.8, 2.5 Hz, 1H; 5-H), 2.88 (sept; *J* = 6.9 Hz, 1H; C*H*), 3.95 (dd, *J* = 12.3, 2.5 Hz, 1H; 6a-H), 4.01 (ddd, *J* = 10.1, 5.2, 2.8 Hz, 1H, 5′-H), 4.13 (dd, *J* = 12.3, 4.8 Hz, 1H; 6b-H), 4.22 (dd, *J* = 12.4, 2.8 Hz, 1H; 6a′-H), 4.25 (dd, *J* = 12.4, 5.2 Hz, 1H; 6b′-H), 5.05 (d, *J* = 8.0 Hz, 1H; 1-H), 5.17 (dd, *J* = 10.0, 9.4 Hz, 1H; 4-H), 5.18 (dd, *J* = 10.1, 9.3 Hz, 1H; 4′-H), 5.27 (d, *J* = 7.8 Hz, 1H; 1′-H), 5.30 (dd, *J* = 9.8, 9.4 Hz, 1H; 3-H), 5.38 (dd, *J* = 9.7, 9.3 Hz, 1H; 3′-H), 5.44 (dd, *J* = 9.7, 7.8 Hz, 1H; 2′-H), 5.44 (dd, *J* = 9.8, 8.0 Hz, 1H; 2-H), 7.02 (s, 1H; 3-ArH), 7.47 (ddd, *J* = 8.4, 6.8, 1.2 Hz, 1H; 6-ArH), 7.53 (ddd, *J* = 8.5, 6.8, 1.3 Hz, 1H; 7-ArH), 8.01 (ddd, *J* = 8.4, 1.3, 0.7 Hz, 1H; 5-ArH), 8.14 ppm (ddd, *J* = 8.5, 1.2, 0.7 Hz, 1H; 8-ArH). ^13^C-NMR (150 MHz, CD_2_Cl_2_): δ = 20.7 (q; OAc-6), 20.8 (q; OAc-4), 20.8 (q; OAc-2), 20.8 (2q; OAc-2′, OAc-3′), 20.9 (q; OAc-4′), 21.0 (q; OAc-6′), 21.0 (q; OAc-2), 23.4 (q; *C*H_3_), 23.9 (q; *C*H_3_), 26.6 (d; *C*H), 62.1 (t; C-6), 62.7 (t; C-6′), 68.7 (d; C-4), 68.7 (d; C-4′), 71.3 (d; C-2′), 72.2 (C-5), 72.2 (d; C-2), 72.7 (d; C-3′), 72.7 (d; C-5′), 73.2 (d; C-3), 100.0 (d; C-1′), 102.3 (d; C-1), 108.0 (d; ArC-3), 121.9 (d; ArC-5), 122.4 (d; ArC-8), 125.6 (s; ArC-4a), 125.9 (s; ArC-6), 127.1 (d; ArC-7), 128.8 (s; ArC-8a), 138.9 (s; ArC-2), 142.8 (s; ArC-1), 150.7 (s; ArC-4), 169.6 (s; OAc-2′), 169.7 (s; OAc-2), 169.8 (s; OAc-4), 169.8 (s; OAc-4′), 170.3 (s; OAc-3′), 170.5 (s; OAc-3), 170.6 (s; OAc-6), 170.8 ppm (s; OAc-6′). IR (Film): v = 3667 (w), 3482 (w), 2969 (m), 2897 (w), 1746 (s), 1601 (w), 1365 (m), 1212 (s), 1171 (m), 1035 cm^−1^ (s). HRMS (ESI-Q-TOF): *m*/*z* Calcd for C_41_H_50_NaO_20_ [M + Na^+^]: 871.2631; Found: 871.2634. Anal. Calcd for C_41_H_50_O_20_ (862.83): C 57.07, H 5.84; Found: C 56.98, H 5.90.

*2-tButyl-1,4-bis-(1-O-2,3,4,6-tetra-O-acetyl-β-d-glucopyranosyl)-naphthalene***1f.** Obtained as a colorless solid (3.106 g, 71%). m.p. = 230–231 °C. *R_f_* = 0.31 (PE/EtOAc 1:1). [α${]}_{D}^{24}$ = −28 (*c* 1.01, CHCl_3_). ^1^H-NMR (600 MHz, CD_2_Cl_2_): δ = 1.49 (s, 9H; *t*-Bu), 1.93 (s, 3H; 6-OAc), 1.98 (s, 3H; 4′-OAc), 2.03 (s, 3H; 3′-OAc), 2.04 (s, 3H; 3-OAc), 2.05 (s, 3H; 4-OAc), 2.05 (s, 3H; 2′-OAc), 2.06 (s, 3H; 6′-OAc), 2.22 (s, 3H; 2-OAc), 3.49 (ddd, *J* = 10.0, 4.2, 2.5 Hz, 1H; 5-H), 3.92 (dd, *J* = 12.3, 2.5 Hz, 1H; 6a-H), 3.99 (ddd, *J* = 10.1, 5.2, 2.9 Hz, 1H, 5′-H), 4.08 (dd, *J* = 12.3, 4.2 Hz, 1H; 6b-H), 4.21 (dd, *J* = 12.4, 2.9 Hz, 1H; 6a′-H), 4.24 (dd, *J* = 12.4, 5.2 Hz, 1H; 6b′-H), 5.17 (dd, *J* = 10.1, 9.4 Hz, 1H; 4-H), 5.18 (dd, *J* = 10.0, 9.3 Hz, 1H; 4′-H), 5.24 (d, *J* = 7.8 Hz, 1H; 1′-H), 5.25 (d, *J* = 8.1 Hz, 1H; 1-H), 5.30 (dd, *J* = 9.8, 9.4 Hz, 1H; 3-H), 5.37 (dd, *J* = 9.8, 9.3 Hz, 1H; 3′-H), 5.43 (dd, *J* = 9.8, 7.8 Hz, 1H; 2′-H), 5.45 (dd, *J* = 9.7, 8.1 Hz, 1H; 2-H), 7.19 (s, 1H; 3-ArH), 7.47 (ddd, *J* = 8.3, 6.8, 1.2 Hz, 1H; 6-ArH), 7.54 (ddd, *J* = 8.5, 6.8, 1.3 Hz, 1H; 7-ArH), 7.99 (ddd, *J* = 8.3, 1.3, 0.6 Hz, 1H; 5-ArH), 8.06 ppm (ddd, *J* = 8.5, 1.2, 0.6 Hz, 1H; 8-ArH). ^13^C-NMR (150 MHz, CD_2_Cl_2_): δ = 20.7 (q; OAc-6), 20.8 (q; OAc-4′), 20.8 (q; OAc-3), 20.8 (2q; OAc-2′, OAc-3′), 20.9 (q; OAc-4), 21.0 (q; OAc-6′), 21.1 (q; OAc-2), 32.3 (q; *t*-Bu), 36.8 (s; *t*-Bu), 61.7 (t; C-6), 62.8 (t; C-6′), 68.6 (d; C-4), 68.7 (d; C-4′), 71.3 (d; C-2′), 72.0 (C-5), 72.3 (d; C-2), 72.7 (d; C-3′), 72.8 (d; C-5′), 73.1 (d; C-3), 100.2 (d; C-1′), 100.8 (d; C-1), 111.1 (d; ArC-3), 122.0 (d; ArC-5), 122.5 (d; ArC-8), 125.9 (d; ArC-6), 126.0 (s; ArC-4a), 126.9 (d; ArC-7), 128.4 (s; ArC-8a), 139.9 (s; ArC-2), 143.2 (s; ArC-1), 149.5 (s; ArC-4), 169.6 (s; OAc-2), 169.7 (s; OAc-2′), 169.8 (s; OAc-4), 169.9 (s; OAc-4′), 170.3 (s; OAc-3), 170.4 (s; OAc-3′), 170.6 (s; OAc-6), 170.8 ppm (s; OAc-6′). IR (Film): v = 2965 (w), 1741 (s), 1434 (w), 1369 (m), 1220 (s), 1034 cm^−1^ (s). HRMS (ESI-Q-TOF): *m*/*z* Calcd for C_42_H_52_NaO_20_ [M + Na^+^]: 899.2944; Found: 899.2949. Anal. Calcd for (%) C_42_H_52_O_20_ (876.86): C 57.53, H 5.98; Found: C 57.37, H 6.06.

### 3.4. Photooxygenation of Carbohydrate-Substituted Naphthalenes ***1*** to Endoperoxides ***2***

A solution of naphthalene **1** (0.15 mmol) and sensitizer tetraphenylporphyrin (TPP, 1.0 mg) in CD_2_Cl_2_ (3.0 mL) was cooled to −70 °C, oxygen was slowly bubbled through the solution, and the mixture was irradiated externally with a sodium lamp (400 W) for 2 h. NMR spectra (500 MHz), which were directly measured from the crude products at −70 °C, showed complete conversion and the sole formation of endoperoxides **2**. The diastereomeric ratios (*dr*) could be easily determined by integration of the distinctive signals of H^3^, which are separated by 0.2 ppm (for NMR spectra, see Supplementary Materials; the characteristic chemical shifts of the minor isomers are marked bold). The reaction of *t*Bu derivative **1f** with ^1^O_2_ was conducted on a preparative scale as well. Thus, naphthalene **1f** (877 mg, 1.0 mmol) and TPP (15 mg) were dissolved in dichloromethane (100 mL) and the solution was photooxygenated at −70 °C for 2 h, as described above. The solvent was removed in vacuo and direct column chromatography (hexanes/EtOAc 1:1) at room temperature afforded endoperoxide 1*S*,4*R*-**2f** (417 mg, 46%) and 1*R*,4*S*-**2f** (373 mg, 41%) in pure form.

*Endoperoxide***2b.***R_f_* = 0.56 (PE/EtOAc 3:1). ^1^H-NMR (500 MHz, CD_2_Cl_2_, −78 °C): δ = 1.93 (s, 3H; OAc), 1.99 (s, 3H; OAc), 1.99 (s, 3H; OAc), 2.00 (s, 6H; OAc), 2.00 (s, 3H; OAc), 2.05 (s, 3H; OAc), 2.06 (s, 3H; OAc), 3.84 (ddd, *J* = 9.9, 4.7, 2.1 Hz, 1H; 5-H), 3.88 (ddd, *J* = 10.0, 3.8, 2.4 Hz, 1H; 5′-H), 4.01 (dd, *J* = 12.5, 2.4 Hz, 1H; 6_a_’-H), 4.06 (dd, *J* = 12.5, 2.1 Hz, 1H; 6_a_-H), 4.28 (m, 2H; 6_b_-H, 6_b_’-H), 5.13 (dd, *J* = 10.0, 9.7 Hz, 1H; 4′-H), 5.16 (dd, *J* = 9.9, 9.5 Hz, 1H; 4-H), 5.19–5.31 (m, 6H; 1-H, 1′-H, 2-H, 2′-H, 3-H, 3′-H); 6.94 (d, *J* = 9.3 Hz, 1H; 3-ArH), 7.16 (d, *J* = 9.3 Hz, 1H; 2-ArH), 7.28–7.33 (m, 2H; 6-ArH, 7-ArH), 7.35–7.39 (m, 1H; 8-ArH), 7.43–7.46 ppm (m, 1H; 5-ArH). ^13^C-NMR (125 MHz, CD_2_Cl_2_, –78 °C): δ = 20.6 (q; 2 x OAc), 20.6 (q; 3 x OAc), 20.7 (q; 2 x OAc), 20.7 (q; OAc), 61.0 (t; C-6), 61.1 (t; C-6′), 66.5 (d; C-4), 66.8 (d; C-4′), 69.4 (d; C-2′), 70.1 (d; C-2), 70.9 (d; C-3′), 71.2 (d; C-3), 71.5 (d; C-5′), 71.6 (d; C-5), 96.5 (d; C-1′), 96.6 (d; C-1), 101.7 (s; ArC-1), 102.8 (s; ArC-4), 119.2 (d; ArC-8), 119.4 (d; ArC-5), 127.5 (d; ArC-6), 127.6 (d; ArC-7), 133.3 (s; ArC-4a), 135.5 (d; ArC-2), 135.9 (s; ArC-8a), 136.0 (d; ArC-3), 169.1 (s; OAc), 169.4 (s; OAc), 169.4 (s; OAc), 169.5 (s; OAc), 170.0 (s; OAc), 170.2 (s; OAc), 170.6 (s; OAc-6), 170.6 ppm (s; OAc-6‘). HRMS (ESI-Q-TOF): *m*/*z* Calcd for C_38_H_45_O_22_ [M + H^+^]: 853.2402; Found: 853.2427.

*Endoperoxide***2c.***R_f_* = 0.51 (PE/EtOAc 3:1). ^1^H-NMR (500 MHz, CD_2_Cl_2_, −78 °C): δ = 1.81 (d, *J* = 1.4 Hz, 3H; Me), 1.94 (s, 3H; OAc), 1.98 (s, 3H; OAc), 1.99 (s, 3H; OAc), 2.00 (s, 3H; OAc), 2.00 (s, 3H; OAc), 2.04 (s, 3H; 6′-OAc), 2.06 (s, 3H; OAc), 2.15 (s, 3H; 6-OAc), 3.82 (ddd, *J* = 9.7, 5.4, 2.1 Hz, 1H; 5-H), 3.89 (ddd, *J* = 9.8, 5.4, 2.3 Hz, 1H; 5′-H), 4.07 (dd, *J* = 12.5, 2.3 Hz, 1H; 6_a_’-H), 4.13 (dd, *J* = 12.3, 2.1 Hz, 1H; 6_a_-H), 4.19−4.25 (m, 2H; 6_b_-H, 6_b_’-H), 5.05 (dd, *J* = 9.8, 9.7 Hz, 1H; 4′-H), 5.15 (dd, *J* = 9.8, 9.7 Hz, 1H; 4-H), 5.13–5.33 (m, 6H; 2-H, 1-H, 2′-H, 1′-H, 3-H, 3′-H), 6.43 (d, *J* = 1.5 Hz, 1H; **3-ArH**), 6.61 (d, *J* = 1.4 Hz, 1H; 3-ArH), 7.26 (ddd, *J* = 7.4, 7.3, 1.2 Hz, 1H; 7-ArH), 7.31 (ddd, *J* = 7.4, 7.4, 1.2 Hz, 1H; 6-ArH), 7.34 (dd, *J* = 7.4, 1.3 Hz, 1H; 5-ArH, **6-ArH**, **7-ArH**), 7.39–7.40 (m, 1H; **5-ArH**), 7.53–7.55 (m, 1H; **8-ArH**), 7.85 ppm (dd, *J* = 7.3, 1.2 Hz, 1H; 8-ArH). ^13^C-NMR (125 MHz, CD_2_Cl_2_, −78 °C)^a^: δ = 14.7 (q; Me), 20.6 (q; 2 x OAc), 20.6 (q; 3 x OAc), 20.7 (q; OAc), 20.8 (q; OAc), 21.0 (q; OAc), 61.4 (t; C-6′), 62.1 (t; C-6), 67.0 (d; C-4), 67.4 (d; C-4′), 69.5 (d; C-2′), 69.8 (d; C-2), 70.7 (d; C-3), 70.9 (d; C-3′), 71.5 (d; C-5′), 71.7 (d; C-5), 96.1 (2d; C-1, C-1‘), 101.7 (s; ArC-4), 104.0 (s; ArC-1), 118.6 (d; ArC-5), 120.3 (d; ArC-8), 125.2 (d; ArC-3), 126.9 (d; ArC-7), 127.7 (d; ArC-6), 134.0 (s; ArC-8a), 138.8 (s; ArC-4a), 149.1 (d; ArC-2), 169.3 (s; OAc), 169.5 (s; OAc), 169.6 (s; OAc), 169.7 (s; OAc), 169.9 (s; OAc), 170.0 (s; OAc), 170.5 (s; OAc-6‘), 170.6 ppm (s; OAc-6).

*Endoperoxide***2d.***R_f_* = 0.44 (PE/EtOAc 2:1). ^1^H-NMR (500 MHz, CD_2_Cl_2_, −20 °C): δ = 0.97 (d, *J* = 7.2 Hz, 3H; CH_3_), 1.97 (s, 3H; OAc), 1.99 (s, 3H; OAc), 2.00 (2s, 6H; 2 x OAc), 2.02 (s, 3H; OAc), 2.03 (s, 3H; OAc), 2.06 (s, 3H; 6′-OAc), 2.14 (s, 3H; 6-OAc), 2.73 (dq; *J* = 7.2, 1.7 Hz, 2H; C*H_2_*), 3.82 (ddd, *J* = 10.0, 6.0, 2.2 Hz, 1H; 5-H), 3.91 (ddd, *J* = 10.0, 6.5, 2.1 Hz, 1H; 5′-H), 4.12 (dd, *J* = 12.2, 2.1 Hz, 1H; 6_a_’-H), 4.19 (dd, *J* = 12.5, 6.0 Hz, 1H; 6_b_-H), 4.20 (dd, *J* = 12.5, 2.2 Hz, 1H; 6_a_-H), 4.27 (dd, *J* = 12.2, 6.5 Hz, 1H; 6‘_b_-H), 5.06−5.11 (m, *2*H; 4-H, 4′-H), 5.16 (dd, *J* = 9.8, 7.8 Hz, 1H; 2-H), 5.17–5.26 (m, 4H; 2′-H, 3′-H, 1-H, 1′-H); 5.31 (dd, *J* = 9.6, 9.0 Hz, 1H; 3′-H), 6.31 (s, 1H; **3-ArH**), 6.54 (t, *J* = 1.7 Hz, 1H; 3-ArH), 7.26 (ddd, *J* = 7.6, 7.5, 1.0 Hz, 1H; 7-ArH), 7.32 (ddd, *J* = 7.5, 7.4, 0.9 Hz, 1H; 6-ArH, **6-ArH**, **7-ArH**), 7.38 (dd, *J* = 7.4, 1.0 Hz, 1H; 5-ArH), 7.45–7.47 (m, 1H; **5-ArH**), 7.55–7.57 (m, 1H; **8-ArH**), 7.88 ppm (dd, *J* = 7.6, 0.9 Hz, 1H; 8-ArH). ^13^C-NMR (125 MHz, CD_2_Cl_2_, –20 °C): δ = 10.2 (q; *C*H_3_), 20.7 (q; OAc), 20.7 (q; OAc), 20.7 (q; 2 x OAc), 20.7 (q; OAc), 20.8 (q; OAc), 20.9 (q; OAc), 21.0 (q; *C*H_3_), 21.1 (q; OAc), 62.3 (t; C-6′), 62.5 (t; C-6), 67.9 (d; C-4‘), 68.1 (d; C-4), 70.2 (d; C-2′), 70.6 (d; C-2), 71.7 (d; C-3‘), 71.8 (d; C-3), 72.3 (d; C-5′), 72.4 (d; C-5), 96.5 (d; C-1), 96.7 (d; C-1‘), 102.5 (s; ArC-4), 104.6 (s; ArC-1), 119.0 (d; ArC-5), 120.7 (d; ArC-8), 123.5 (d; ArC-3), 127.2 (d; ArC-7), 127.9 (d; ArC-6), 135.2 (s; ArC-8a), 139.6 (s; ArC-4a), 155.1 (d; ArC-2), 169.5 (s; OAc), 169.7 (s; OAc), 169.7 (s; OAc), 169.8 (s; OAc), 170.0 (s; OAc), 170.1 (s; OAc), 170.6 (s; OAc-6), 170.7 ppm (s; OAc-6‘).

*Endoperoxide***2e.***R_f_* = 0.47 (PE/EtOAc 2:1). ^1^H-NMR (500 MHz, CD_2_Cl_2_, −20 °C): δ = 0.90 (d, *J* = 6.7 Hz, 3H; C*H_3_*), 1.04 (d, *J* = 6.8 Hz, 3H; C*H_3_*), 1.97 (s, 3H; OAc), 1.99 (s, 3H; OAc), 2.00 (s, 3H; OAc), 2.00 (s, 3H; OAc), 2.02 (s, 3H; OAc), 2.03 (s, 3H; 6′-OAc), 2.05 (s, 3H; 2-OAc), 2.14 (s, 3H; 6-OAc), 2.73 (dsept; *J* = 6.8, 1.4 Hz, 1H; C*H*), 3.83 (ddd, *J* = 10.1, 6.5, 2.4 Hz, 1H; 5-H), 3.91 (ddd, *J* = 10.0, 6.0, 2.1 Hz, 1H; 5′-H), 4.12 (dd, *J* = 12.3, 2.1 Hz, 1H; 6_a_’-H), 4.17 (dd, *J* = 12.5, 6.0 Hz, 1H; 6_b_-H), 4.19 (dd, *J* = 12.5, 2.4 Hz, 1H; 6_a_-H), 4.24 (dd, *J* = 12.3, 6.5 Hz, 1H; 6‘_b_-H), 5.08 (dd, *J* = 10.0, 9.6 Hz, 1H; 4′-H), 5.10 (dd, *J* = 10.1, 9.2 Hz, 1H; 4-H), 5.17 (dd, *J* = 9.5, 7.6 Hz, 1H; 2-H), 5.21 (dd, *J* = 9.7, 7.8 Hz, 1H; 2′-H), 5.22–5.27 (m, 3H; 3-H, 1-H, 1′-H); 5.31 (dd, *J* = 9.7, 9.6 Hz, 1H; 3′-H), 6.25 (d, *J* = 1.6 Hz, 1H; **3-ArH**), 6.50 (d, *J* = 1.4 Hz, 1H; 3-ArH), 7.25 (ddd, *J* = 7.6, 7.5, 1.2 Hz, 1H; 7-ArH), 7.31 (ddd, *J* = 7.5, 7.5, 0.9 Hz, 1H; 6-ArH, **6-ArH**, **7-ArH**), 7.38 (dd, *J* = 7.4, 1.2 Hz, 1H; 5-ArH), 7.45–7.47 (m, 1H; **5-ArH**), 7.59–7.61 (m, 1H; **8-ArH**), 7.89 ppm (dd, *J* = 7.4, 1.0 Hz, 1H; 8-ArH). ^13^C-NMR (125 MHz, CD_2_Cl_2_, –20 °C): δ = 20.1 (q; *C*H_3_), 20.7 (q; 2 x OAc), 20.7 (q; 2 x OAc), 20.7 (q; OAc), 20.8 (q; OAc), 20.8 (q; *C*H_3_), 20.9 (q; OAc), 21.0 (q; OAc), 27.0 (d; *C*H), 62.4 (t; C-6′), 62.6 (t; C-6), 67.9 (d; C-4‘), 68.1 (d; C-4), 70.2 (d; C-2′), 70.7 (d; C-2), 71.8 (d; C-3‘), 71.9 (d; C-3), 72.3 (d; C-5′), 72.4 (d; C-5), 96.5 (d; C-1), 96.7 (d; C-1‘), 102.4 (s; ArC-4), 104.9 (s; ArC-1), 119.1 (d; ArC-5), 120.5 (d; ArC-8), 122.4 (d; ArC-3), 127.2 (d; ArC-7), 127.9 (d; ArC-6), 135.7 (s; ArC-8a), 139.6 (s; ArC-4a), 159.3 (d; ArC-2), 169.5 (s; OAc), 169.7 (s; OAc), 169.7 (s; OAc), 169.7 (s; OAc), 170.0 (s; OAc), 170.1 (s; OAc), 170.7 (s; OAc-6), 170.7 ppm (s; OAc-6‘).

*Endoperoxide* 1*S*,4*R*-**2f.** *R_f_* = 0.19 (PE/EtOAc 1:1). [α${]}_{D}^{24}$ = +13 (*c* 1.04, CHCl_3_). ^1^H-NMR (600 MHz, CD_2_Cl_2_): δ = 1.15 (s, 9H; ^t^Bu), 1.98 (s, 3H; 3′-OAc), 1.99 (s, 3H; 2-OAc), 2.01 (s, 3H; 3-OAc), 2.02 (2s, 6H; 6′-OAc, 4′-OAc), 2.03 (s, 3H; 4-OAc), 2.06 (s, 3H; 2′-OAc), 2.14 (s, 3H; 6-OAc), 3.81 (ddd, *J* = 10.0, 5.1, 3.8 Hz, 1H; 5-H), 3.93 (ddd, *J* = 10.1, 4.5, 3.9 Hz, 1H; 5′-H), 4.15–4.18 (m, 2H; 6a′-H, 6b′-H), 4.21–4.28 (m, 2H; 6a-H, 6b-H), 5.09 (dd, *J* = 10.1, 9.4 Hz, 1H; 4′-H), 5.13 (dd, *J* = 10.0, 9.3 Hz, 4-H), 5.19–5.22 (m, 3H; 2′-H, 2-H, 3′-H), 5.28 (d, *J* = 7.6 Hz, 1H; 1-H), 5.30 (d, *J* = 7.8 Hz, 1H; 1′-H), 5.32 (dd, *J* = 9.7, 9.3 Hz, 1H; 3-H), 6.57 (s, 1H; 3-ArH), 7.25 (ddd, *J* = 7.6, 7.5, 1.3 Hz, 1H; 7-ArH), 7.31 (ddd, *J* = 7.6, 7.5, 1.1 Hz, 1H; 6-ArH), 7.41 (ddd, *J* = 7.5, 1.3, 0.5 Hz, 1H; 5-ArH). 7.94 ppm (ddd, *J* = 7.5, 1.1, 0.5 Hz, 1H; 8-ArH). ^13^C-NMR (150 MHz, CD_2_Cl_2_): δ = 20.8 (q; OAc-3′), 20.8 (q; OAc-2), 20.8 (q; OAc-3), 20.8 (q; OAc-6′), 20.9 (q; OAc-4′), 20.9 (q; OAc-4), 21.0 (q; OAc-2′), 21.0 (q; OAc-6), 28.4 (q; ^t^Bu), 35.4 (s; ^t^Bu), 62.8 (t; C-6′), 63.0 (t; C-6), 68.7 (d; C-4′), 68.7 (d; C-4), 70.9 (d; C-2), 71.4 (d; C-2′), 72.4 (d; C-3), 72.9 (d; C-5′), 72.9 (d; C-3′), 72.9 (d; C-5), 97.1 (d; C-1′), 97.3 (d; C-1), 102.4 (s; ArC-4), 107.3 (s; ArC-1), 119.6 (d; ArC-5), 120.8 (d; ArC-8), 124.5 (d; ArC-3), 127.3 (d; ArC-7), 128.0 (d; ArC-6), 137.0 (s; ArC-8a), 140.2 (s; ArC-4a), 161.3 (s; ArC-2), 169.7 (2s; OAc-2, OAc-2′), 169.8 (s; OAc-4), 169.9 (s; OAc-4′), 170.1 (s; OAc-3′), 170.2 (s; OAc-3), 170.7 (s; OAc-6), 170.8 ppm (s; OAc-6′). IR (Film): v = 3387 (br), 2927 (w), 1579 (w), 1395 (m), 1266 (w), 1239 (w), 1147 (m), 1072 (s), 1036 cm^−1^ (s). HRMS (ESI-Q-TOF): *m*/*z* Calcd for C_42_H_52_NaO_22_ (M + Na^+^): 931.2842; Found: 931.2842.

*Endoperoxide* 1*R*,4*S*-**2f.** *R_f_* = 0.12 (PE/EtOAc 1:1). [α${]}_{D}^{24}$ = −2.5 (*c* 1.01, CHCl_3_). ^1^H-NMR (600 MHz, CD_2_Cl_2_): δ = 1.15 (s, 9H; ^t^Bu), 1.98 (s, 3H; 4-OAc), 1.99 (s, 3H; 2′-OAc), 2.00 (s, 3H; 3-OAc), 2.00 (s, 3H; 3′-OAc), 2.01 (s, 3H; 4′-OAc), 2.03 (s, 3H; 6-OAc), 2.05 (s, 3H; 6′-OAc), 2.14 (s, 3H; 2-OAc), 3.70 (ddd, *J* = 10.1, 4.8, 2.5 Hz, 1H; 5-H), 3.86 (ddd, *J* = 10.0, 4.5, 2.4 Hz, 1H; 5′-H), 4.06 (dd, *J* = 12.3, 2.5 Hz, 1H; 6a-H), 4.08 (dd, *J* = 12.4, 2.4 Hz, 1H; 6a′-H), 4.23 (dd, *J* = 12.3, 4.8 Hz, 1H; 6b-H), 4.30 (dd, *J* = 12.4, 2.4 Hz, 6b’-H), 5.13 (dd, *J* = 10.1, 9.2 Hz, 1H; 4-H), 5.15 (dd, *J* = 10.0, 9.5 Hz, 1H; 4′-H), 5.23 (dd, *J* = 9.7, 8.0 Hz, 1H; 2′-H), 5.28 (dd, *J* = 9.6, 9.2 Hz, 1H; 3-H), 5.29 (d, *J* = 8.0 Hz, 1H: 1′-H), 5.31 (dd, *J* = 9.7, 9.5 Hz, 1H; 3′-H), 5.37 (d, *J* = 8.1 Hz, 1H; 1-H), 5.37 (dd, *J* = 9.6, 8.1 Hz, 1H; 2-H), 6.28 (s, 1H; 3-ArH), 7.30–7.33 (m, 2H; 6-ArH, 7-ArH), 7.47–7.49 (m, 1H; 5-ArH), 7.64–7.67 ppm (m, 1H; 8-ArH). ^13^C-NMR (150 MHz, CD_2_Cl_2_): δ = 20.7 (q; OAc-4), 20.7 (3q; OAc-2′, OAc-3, OAc-4′), 20.8 (q; OAc-3′), 20.8 (q; OAc-6), 20.8 (q; OAc-6′), 20.9 (q; OAc-2), 28.2 (q; ^t^Bu), 35.5 (s; ^t^Bu), 61.9 (t; C-6), 62.0 (t; C-6′), 68.2 (d; C-4′), 68.6 (d; C-4), 71.6 (d; C-2′), 71.8 (d; C-2), 72.5 (d; C-5), 72.8 (2d; C-5′, C-3′), 73.2 (d; C-3), 97.1 (d; C-1), 97.2 (d; C-1′), 103.2 (s; ArC-4), 107.3 (s; ArC-1), 119.9 (d; ArC-5), 120.1 (d; ArC-8), 124.6 (d; ArC-3), 127.3 (d; ArC-7), 128.0 (d; ArC-6), 136.7 (s; ArC-8a), 140.2 (s; ArC-4a), 161.5 (s; ArC-2), 169.1 (s; OAc-2), 169.2 (s; OAc-2′), 169.7 (s; OAc-4′), 169.7 (s; OAc-4), 170.3 (s; OAc-3), 170.3 (s; OAc-3′), 170.7 (s; OAc-6), 170.7 ppm (s; OAc-6′). IR (Film): v = 3387 (br), 2927 (w), 1579 (w), 1395 (m), 1266 (w), 1239 (w), 1147 (m), 1072 (s), 1036 cm^−1^ (s). HRMS (ESI-Q-TOF): *m*/*z* Calcd for C_42_H_52_NaO_22_ (M + Na^+^): 931.2842; Found: 931.2852.

### 3.5. Thermolysis of Endoperoxides ***2*** and Determination of Singlet Oxygen Yield

The half lifetimes of endoperoxides **2** were determined by measuring UV/VIS spectra at different temperatures and observing the increasing absorption band of the corresponding naphthalene **1** at 290–320 nm. The singlet oxygen yield was determined by trapping with tetramethylethylene. Thus, the cold solution of crude endoperoxide **2b** was purged with argon for 2 min, 20 equiv. of tetramethylethylene were added, and the solution was warmed to room temperature within 8 h. The ^1^O_2_ yield was then determined by ^1^H-NMR integrals of the formed hydroperoxide.

### 3.6. Transformation of Endoperoxide 1S,4R-***2f*** into Enantiomerically Pure Epoxide 2S,3R-***8***

Endoperoxide 1*S*,4*R*-**2f** (360 mg, 0.4 mmol) was dissolved in dichloromethane (10 mL), cooled to 0 °C, and a cold solution of *m*-chloroperoxybenzoic acid (70 mg, 0.4 mmol) in dichloromethane (10 mL) was added at 0 °C. After 16 h at 0 °C, TLC showed incomplete conversion and another amount of *m*-chloroperoxybenzoic acid (35 mg, 0.2 mmol) in dichloromethane (5 mL) was added. After 2 days at room temperature, the reaction mixture was extracted with a saturated solution of potassium carbonate (2 × 20 mL), washed with water (20 mL), and dried over sodium sulfate. After removal of solvent in vacuo, the crude product was purified by column chromatography (hexanes/EtOAc 1:1) to afford epoxide *syn*-**7** (280 mg, 76%) as sole diastereomer.

*R_f_* = 0.16 (PE/EtOAc 1:1). [α${]}_{D}^{24}$ = +66 (*c* 0.99, CHCl_3_). ^1^H-NMR (400 MHz, CDCl_3_): δ = 0.92 (s, 9H; ^t^Bu), 1.99 (s, 3H; OAc), 2.02 (s, 3H; OAc), 2.03 (s, 3H; OAc), 2.03 (s, 3H; OAc), 2.04 (s, 3H; OAc), 2.06 (s, 3H; OAc), 2.15 (s, 3H; OAc), 2.18 (s, 3H; OAc), 3.77–3.82 (m, 1H; 5-H), 3.92 (s, 1H; 3-H), 3.93–4.00 (m, 1H; 5′-H), 4.19–4.28 (m, 4H; 6a′-H, 6b′-H, 6a-H, 6b-H), 5.09–5.26 (m, 6H; 1′-H, 2-H, 2′-H, 3′-H, 4-H, 4′-H), 5.32–5.38 (m, 2H; 3-H, 1-H), 7.42–7.57 (m, 3-ArH; 5-ArH, 6-H, 7-ArH), 8.03–8.06 ppm (m, 8-ArH). ^13^C-NMR (100 MHz, CDCl_3_): δ = 20.8 (2q; OAc), 20.8 (3q; OAc), 20.8 (q; OAc), 21.0 (q; OAc), 21.0 (q; OAc), 26.5 (q; ^t^Bu), 32.9 (s; ^t^Bu), 61.0 (d; C-3), 62.5 (t; C-6′), 62.8 (t; C-6), 68.6 (d; C-4′), 68.9 (d; C-4), 70.9 (d; C-2), 71.3 (d; C-2′), 71.4 (s; ArC-2), 72.4 (d; C-3), 72.6 (d; C-5′), 73.0 (d; C-3′), 73.1 (d; C-5), 97.1 (d; C-1′), 97.1 (d; C-1), 100.1 (s; ArC-4), 106.4 (s; ArC-1), 121.3 (d; ArC-5), 123.5 (d; ArC-8), 129.1 (d; ArC-7), 129.3 (d; ArC-6), 137.0 (d; ArC-8a), 138.4 (s; ArC-4a),169.6 (s; OAc), 169.9 (3s; OAc), 170.1 (s; OAc), 170.2 (s; OAc), 170.7 (s; OAc), 170.7 ppm (s; OAc-6′). IR (Film): ν = 3660 (w), 3501 (w), 1743 (s), 1434 (m), 1364 (s), 1214 (s), 1037 (s). HRMS (ESI-Q-TOF): *m*/*z* Calcd for C_42_H_52_NaO_23_ (M + Na^+^): 947.2792, Found: 947.2790.

Epoxide *syn*-**7** (190 mg, 0.2 mmol) was dissolved in dichloromethane (10 mL), and palladium on charcoal (20 mg, 5 mol%) was added. The solution was purged with hydrogen gas for 5 min, equipped with a balloon filled with hydrogen gas and hydrogenated under stirring at 1 bar for 16 h. The solvent was removed in vacuo, and column chromatography (hexanes/EtOAc 1:1) afforded epoxide **8** (30 mg, 63%) as a white solid in enantiomerically pure form, which was confirmed by HPLC on a chiral phase (for chromatograms, see Figure 5).

M.p. = 61–63 °C. *R_f_* = 0.81 (PE/EtOAc 1:1). [α${]}_{D}^{25}$ = −22 (*c* 1.00, CHCl_3_). ^1^H-NMR (400 MHz, CDCl_3_): δ = 1.23 (s, 9H; ^t^Bu), 3.99 (s, 1H; 3-H), 7.67–7.76 (m, 2H; 5-H, 6-H), 7.88–7.91 (m, 1H; 3-H, 5-H), 7.96–7.99 ppm (m, 1H; 8-H). ^13^C-NMR (100 MHz, CDCl_3_): δ = 26.3 (q; *t*-Bu), 33.0 (s; *t*-Bu), 58.8 (d; C-3), 68.4 (s; C-2), 126.3 (d; C-5), 127.8 (d; C-8), 131.3 (s; C-4a), 134.0 (d; C-6), 134.2 (s; C-8a), 134.7 (d; C-7), 191.5 (s; C-1), 192.7 ppm (s; C-4). IR (Film): ν = 3484(w, br), 2962 (w), 2921 (w), 2877 (w), 1695 (s), 1596 (s), 1461 (m), 1341 (m), 1322 (m), 1299 (s), 1143 (s), 916 (s), 881 (s). LRMS (GC-TOF): *m*/*z* Calcd for C_14_H_14_O_3_ (M^+^): 230.0943; Found: 230.0941.

## 4. Conclusions

In conclusion, we could control the [4+2] cycloaddition of singlet oxygen to naphthalenes by carbohydrates. Such a chiral auxiliary approach with sugars was hitherto unknown. The precursors are easily available from naphthoquinone and Schmidt’s trichloroacetimidate in only 2–3 steps. The photooxygenations afforded endoperoxides (EPOs) as sole products, which were characterized by low-temperature NMR, but can reconvert to the parent naphthalenes under release of singlet oxygen. However, sterically demanding EPOs could be isolated by column chromatography in enatiomerically pure form at room temperature for the first time. Their absolute configurations were determined by a combination of NOESY measurements and theoretical calculations. We found a remarkable influence of substituents on the diastereoselectivities of the photooxygenations, ranging from no selectivity to up to 91:9 *dr*, which was rationalized by preferred rotamers of the carbohydrates. Thus, the sugars control the attack of singlet oxygen by steric and electrostatic repulsions. Finally, we could transfer the chiral information of the EPOs to an epoxide with high selectivity. After removal of the carbohydrates, we obtained a naphthoquinone epoxide in enantiomerically pure form, proven by HPLC on a chiral phase. This allowed the unequivocal determination of the absolute configurations of the EPOs. Furthermore, since the carbohydrates can be easily introduced, influence the selectivities of the photooxygenations, and are finally cleaved, the concept of a chiral auxiliary controlled [4+2] cycloaddition of singlet oxygen to naphthalenes has been realized.

## Figures and Tables

**Table 1:** Photooxygenation of naphthalenes **1b**–**f** to endoperoxides **2b**–**f** ^1^.

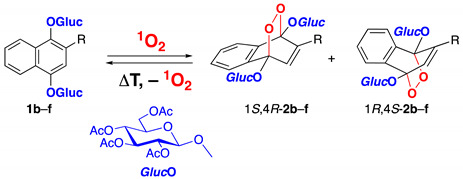					
Entry	Naphthalene 1	R	Temperature, Conversion ^2^	t_1/2_ (20 °C) 2 ^3^	*dr* 1*S*,4*R*-2:1*R*,4*S*-2 ^2^
1	**1b**	H	20 °C, <5%	2 min	–
2	**1b**	H	−70 °C, >98%	2 min	–
3	**1c**	Me	−70 °C, >98%	7 min	90:10
4	**1d**	Et	−70 °C, >98%	9 min	91:9
5	**1e**	*i*Pr	−70 °C, >98%	48 min	89:11
6	**1f**	*t*Bu	−70 °C, >98%	>4 h ^4^	51:49 ^5^

^1^ Reactions were performed on a 0.15 mmol NMR scale. For procedure and conditions, see Materials and Methods. ^2^ Determined by NMR (500 MHz) of the crude products. ^3^ Determined by UV measurements. ^4^ For 77 min at 60 °C. ^5^ On a larger 1.0 mmol scale, diastereomeric EPOs **2f** could be separated by column chromatography and isolated in 46% and 41% in analytically pure form.

## Data Availability

The data presented in this study are available in this article.

## Supplementary Materials

The following are available online, S2–S5: ^1^H- and ^13^C-NMR spectra of naphthoquinones **3**, S6–S10: ^1^H- and ^13^C-NMR spectra of carbohydrate-substituted naphthalenes **1**, S11–S17: ^1^H- and ^13^C-NMR spectra of endoperoxides **2**, S18–S19: NOESY spectra of endoperoxides **2f,** S20–S21: ^1^H- and ^13^C-NMR spectra of epoxides **7** and **8**, S22–S30: theoretical calculations.

## Sample Availability

Not available.
