# Supplementary material for: Stereoselective [4+2] Cycloaddition of Singlet Oxygen to Naphthalenes Controlled by Carbohydrates
Source: Molecules. 2021 Feb 4;26(4):804. doi: 10.3390/molecules26040804 (PMC7913910; doi:10.3390/molecules26040804)
Supplement: Supplementary file 1 [file molecules-26-00804-s001.pdf]

molecules

MDPI

*Article*

# Stereoselective [4+2] Cycloaddition of Singlet Oxygen to Naphthalenes Controlled by Carbohydrates

**Marcel Bauch, Werner Fudickar and Torsten Linker[1]***

[1] Department of Chemistry, University of Potsdam, Karl-Liebknecht-Str. 24-25, 14476 Golm, Germany
* Correspondence: linker@uni-potsdam.de

Received: date; Accepted: date; Published: date

## Supplementary Materials

## Table of Contents

1. **NMR Spectra of naphthoquinones 3** **S2 – S5**
2. **NMR Spectra of carbohydrate naphthalenes 1** **S6 – S10**
3. **NMR Spectra of endoperoxides 2** **S11 – S17**
4. **NOESY spectra of endoperoxides 2f** **S18 – S19**
5. **NMR Spectra of epoxides 7 and 8** **S20 – S21**
6. **Theoretical calculations** **S22 – S30**

1. **NMR Spectra of naphthoquinones 3**

**$^1$H NMR (500 MHz, $CDCl_3$) of 3c**

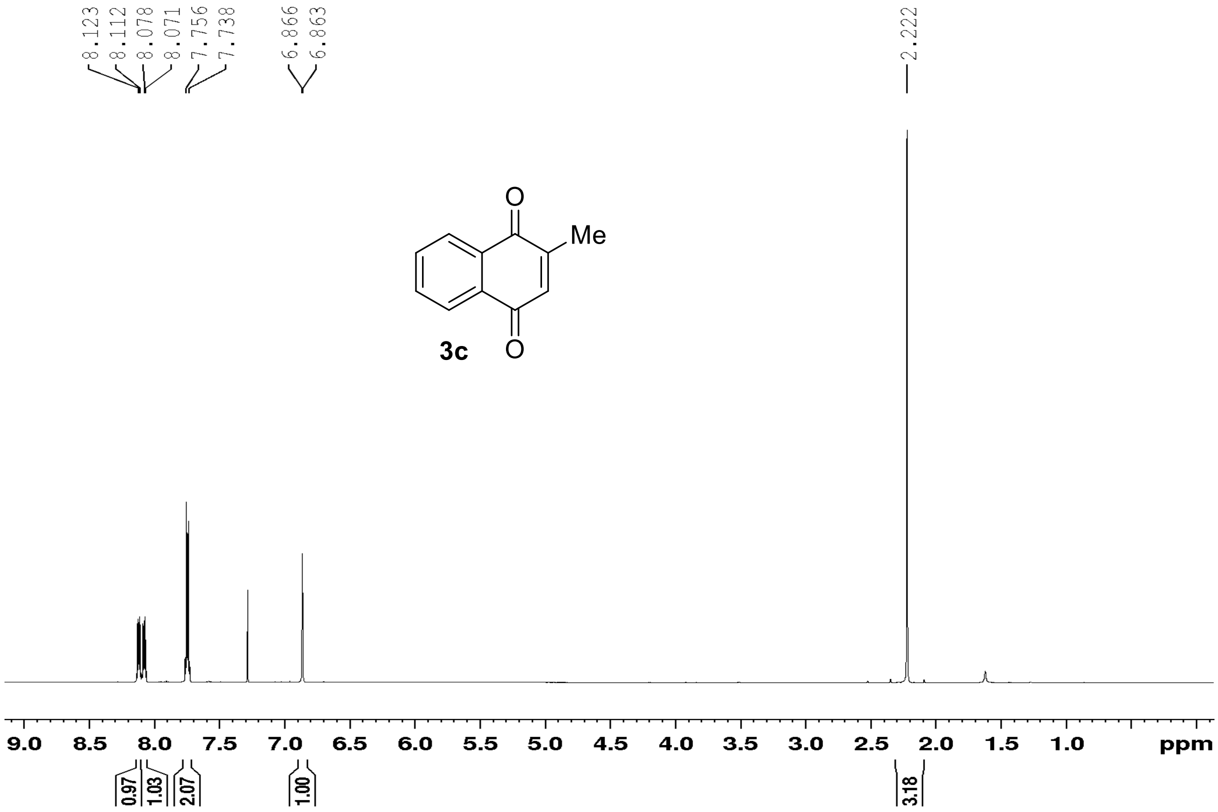


**$^{13}$C NMR (125 MHz, $CDCl_3$) of 3c**

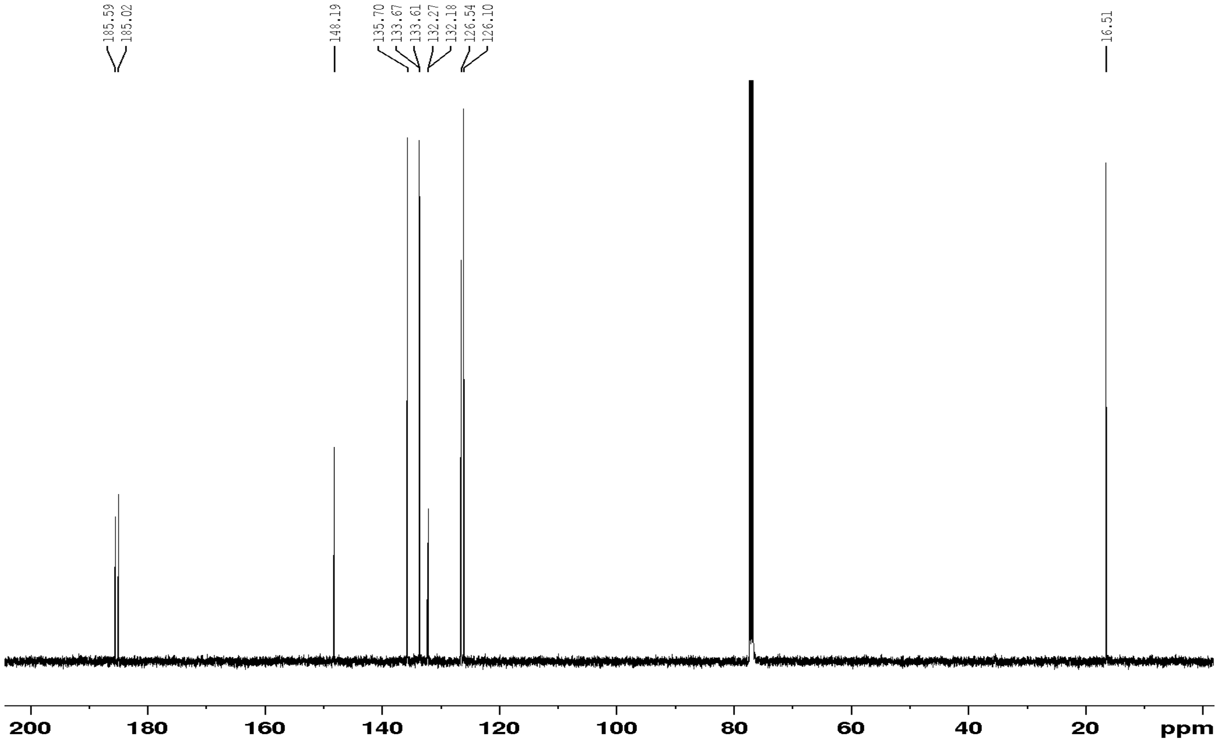

## $^{1}H$ NMR (600 MHz, $CD_2Cl_2$) of 3d

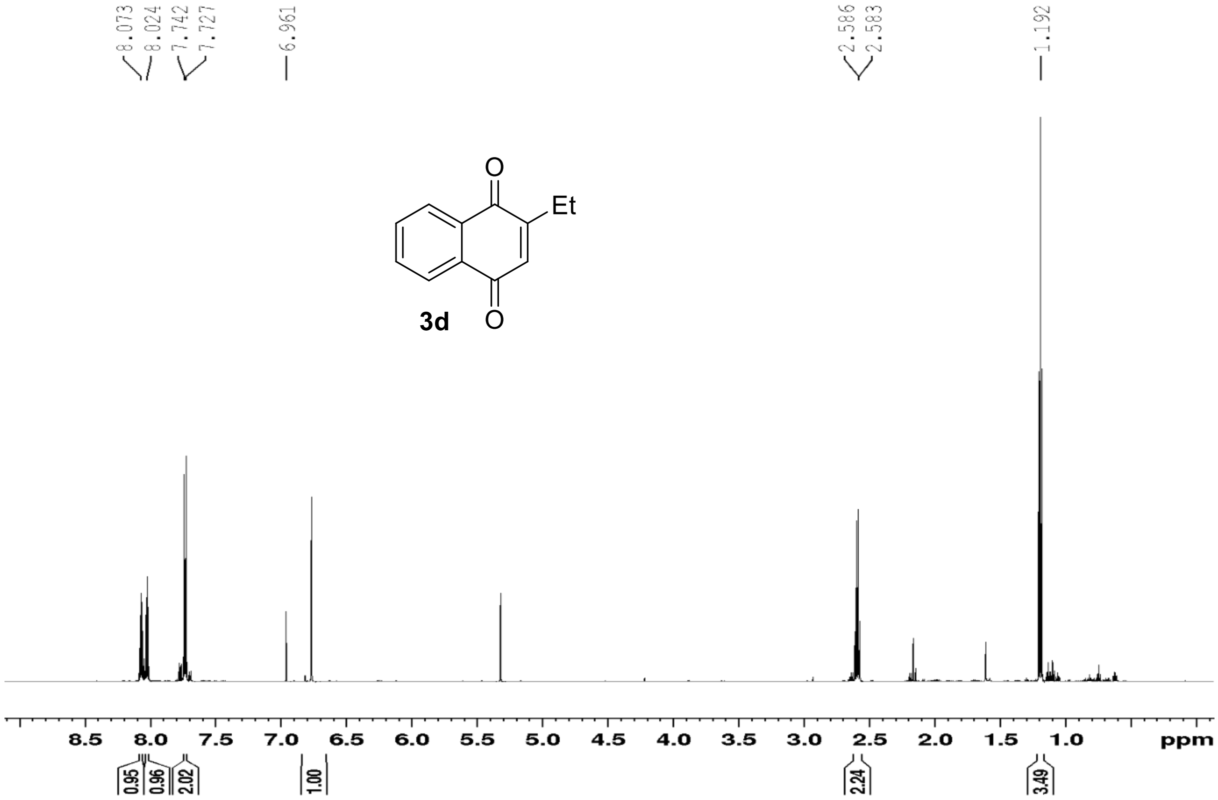


## $^{13}C$ NMR (150 MHz, $CD_2Cl_2$) of 3d

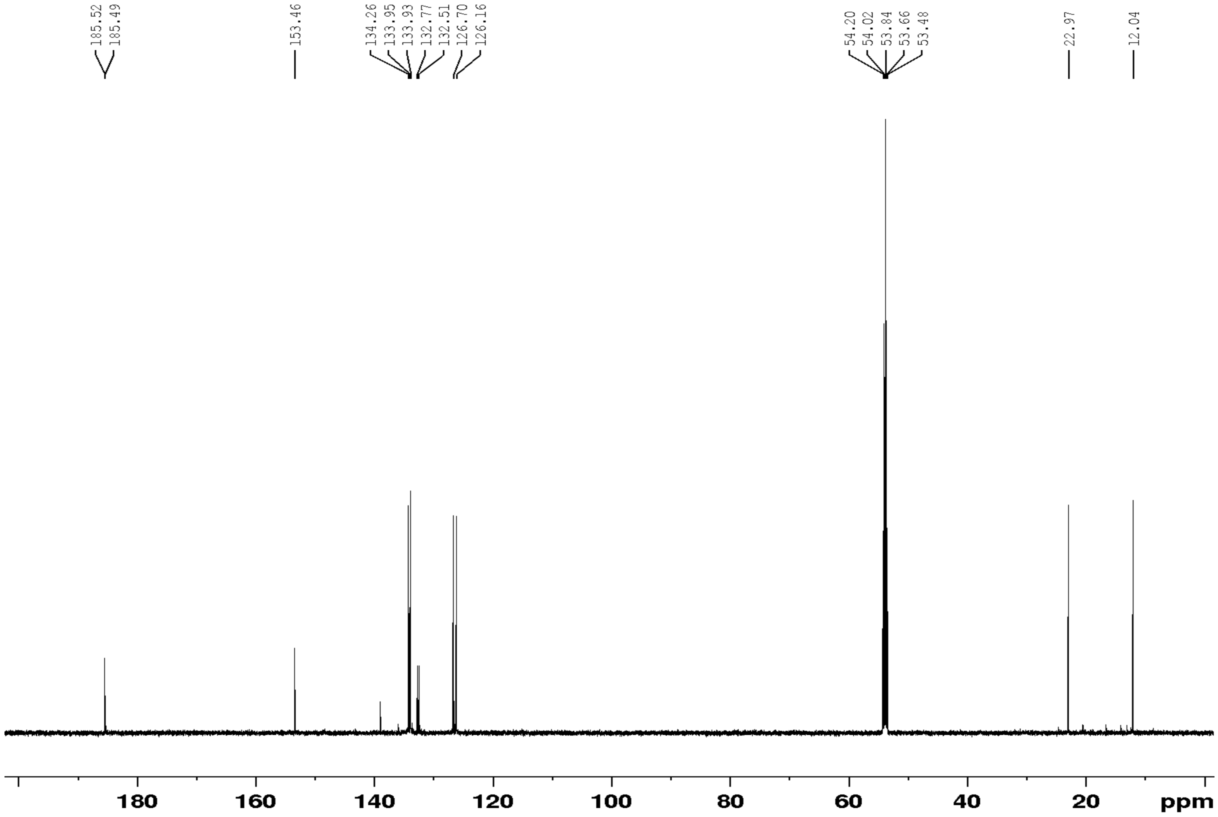

## $^{1}H$ NMR (600 MHz, $CD_2Cl_2$) of 3e

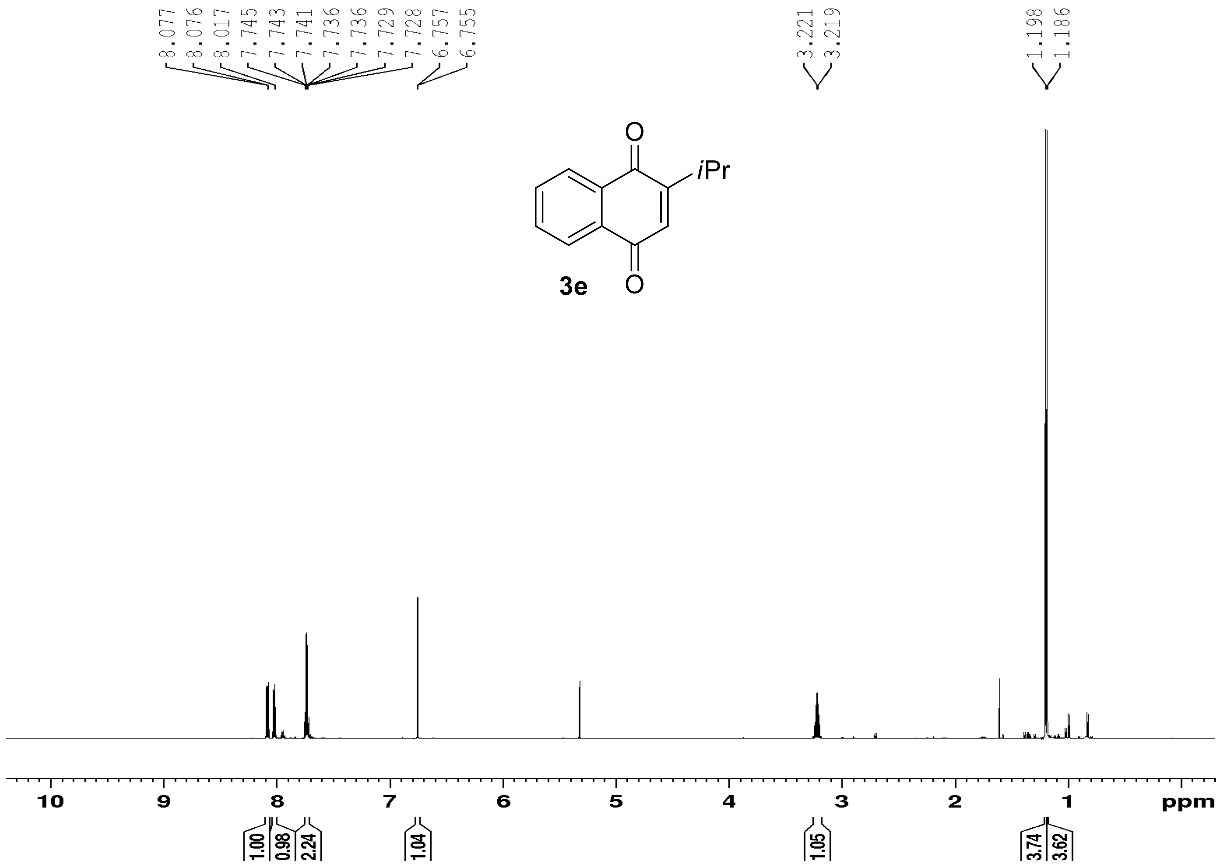


## $^{13}C$ NMR (150 MHz, $CD_2Cl_2$) of 3e

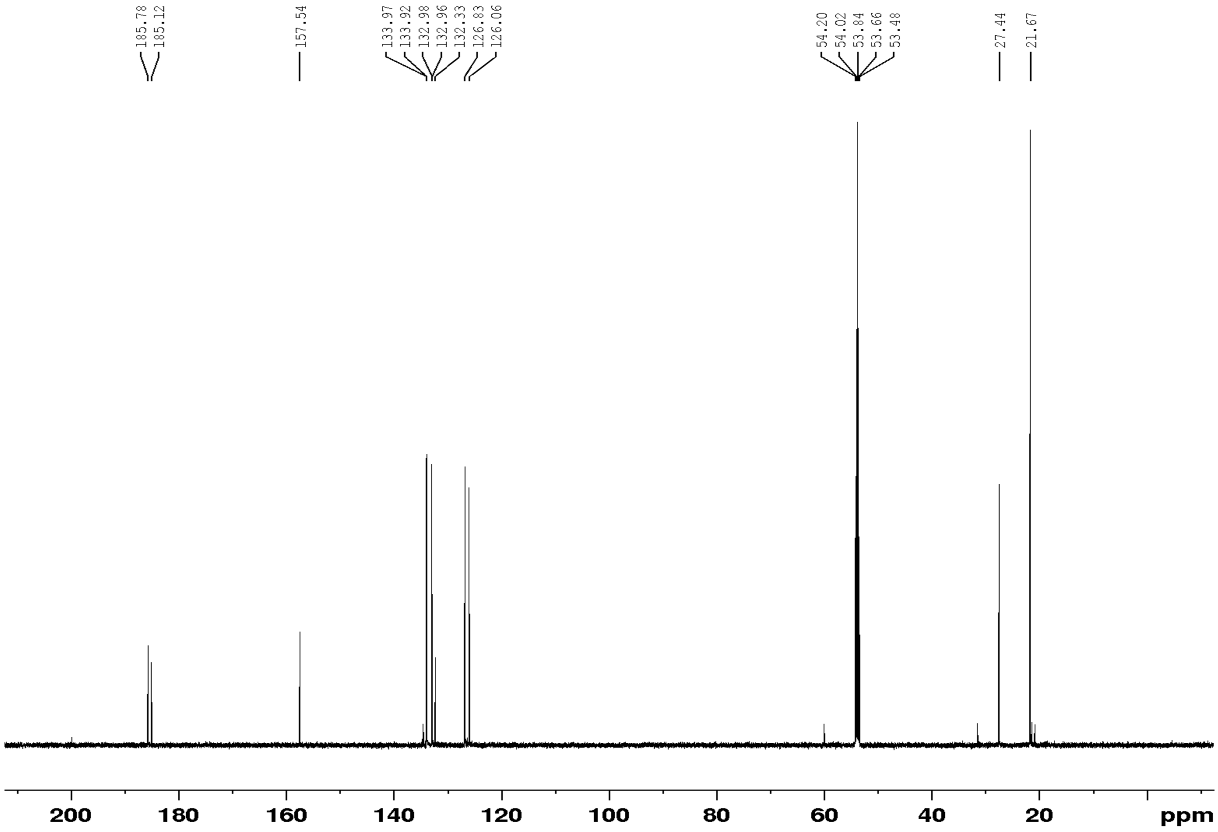

## $^1$H NMR (600 MHz, $CD_2Cl_2$) of 3f

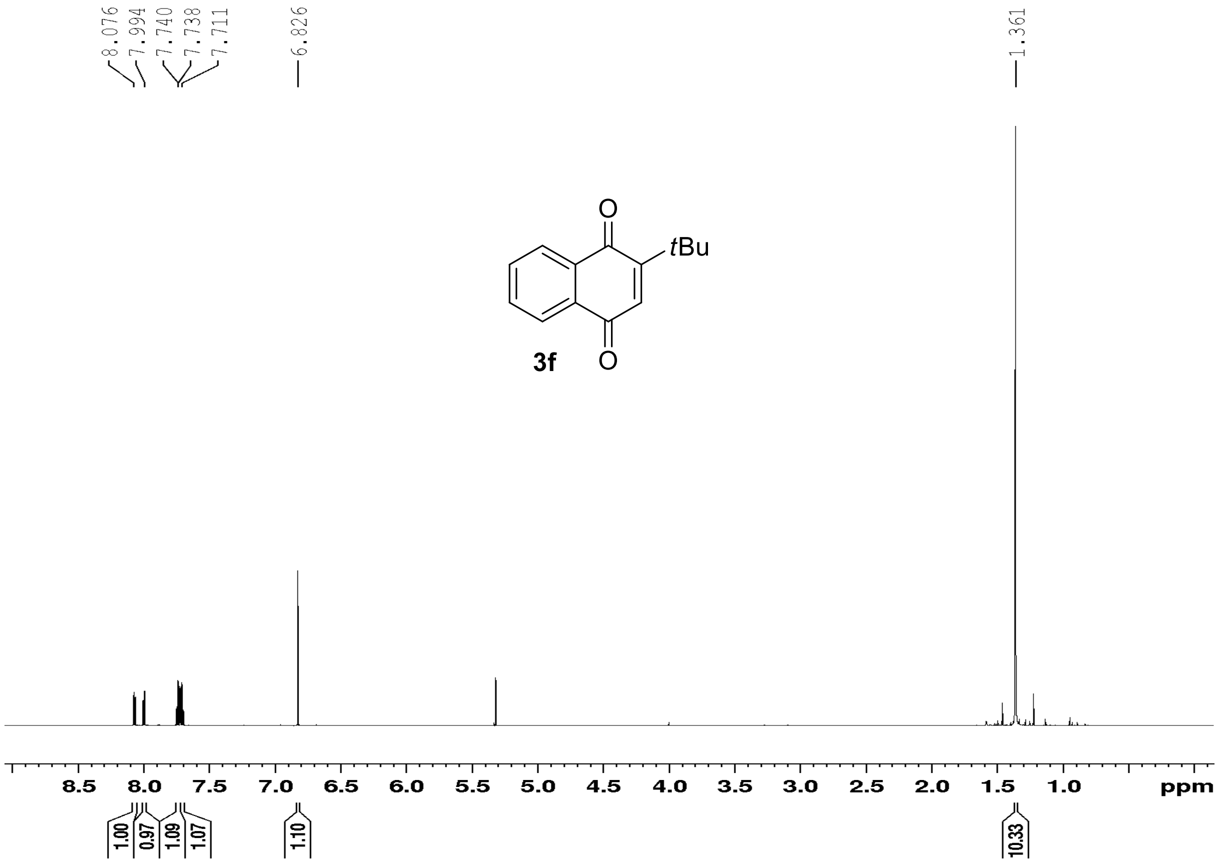


## $^{13}$C NMR (150 MHz, $CD_2Cl_2$) of 3f

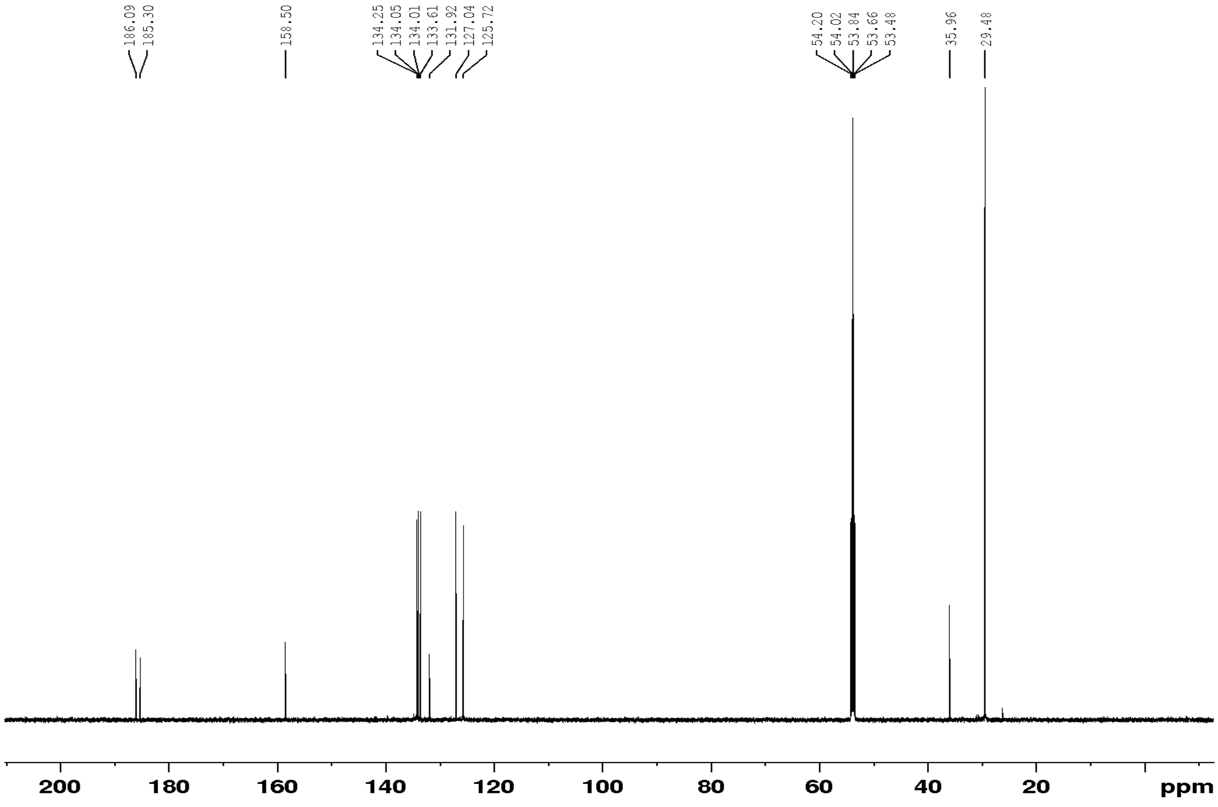

2. **NMR Spectra of carbohydrate naphthalenes 1**

## $^1$H NMR (600 MHz, $CD_2Cl_2$) of 1b

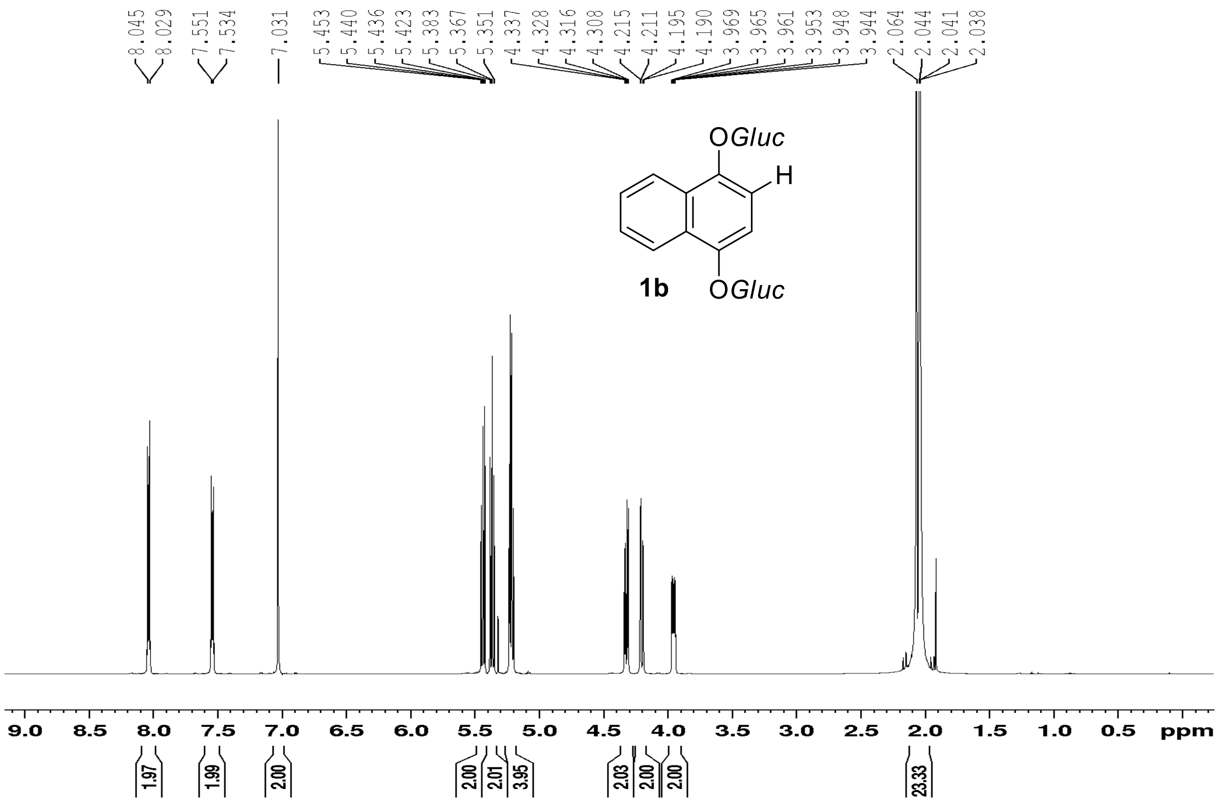


## $^{13}$C NMR (150 MHz, $CD_2Cl_2$) of 1b

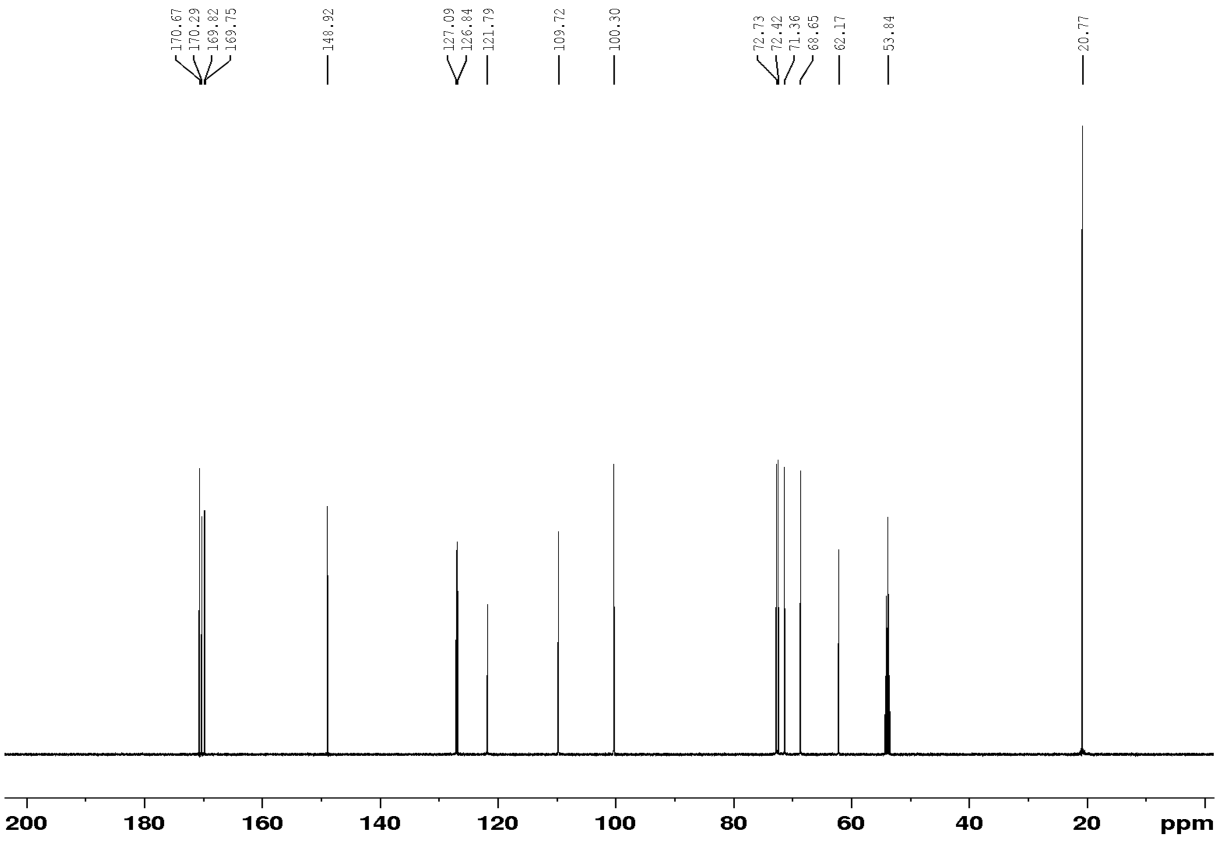

## $^{1}H$ NMR (600 MHz, $CD_2Cl_2$) of 1c

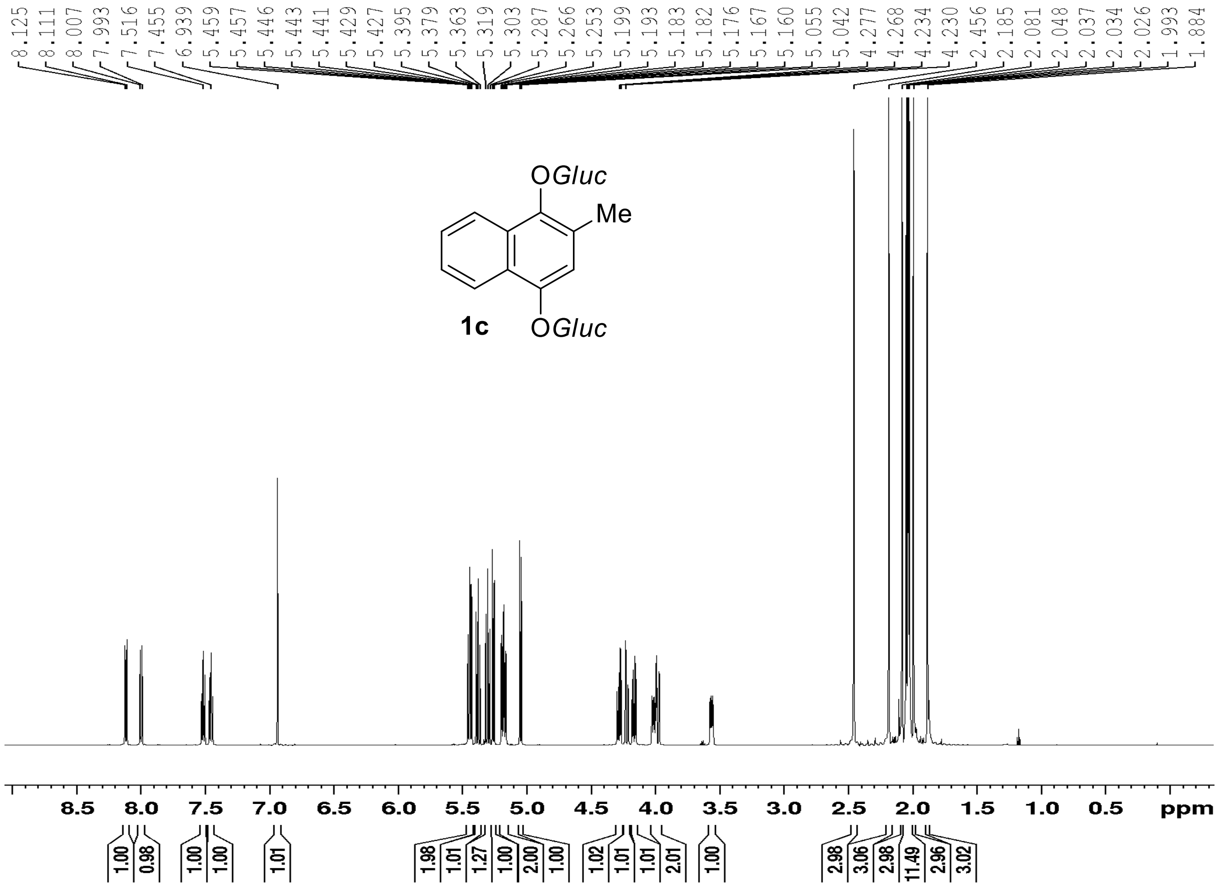


## $^{13}C$ NMR (150 MHz, $CD_2Cl_2$) of 1c

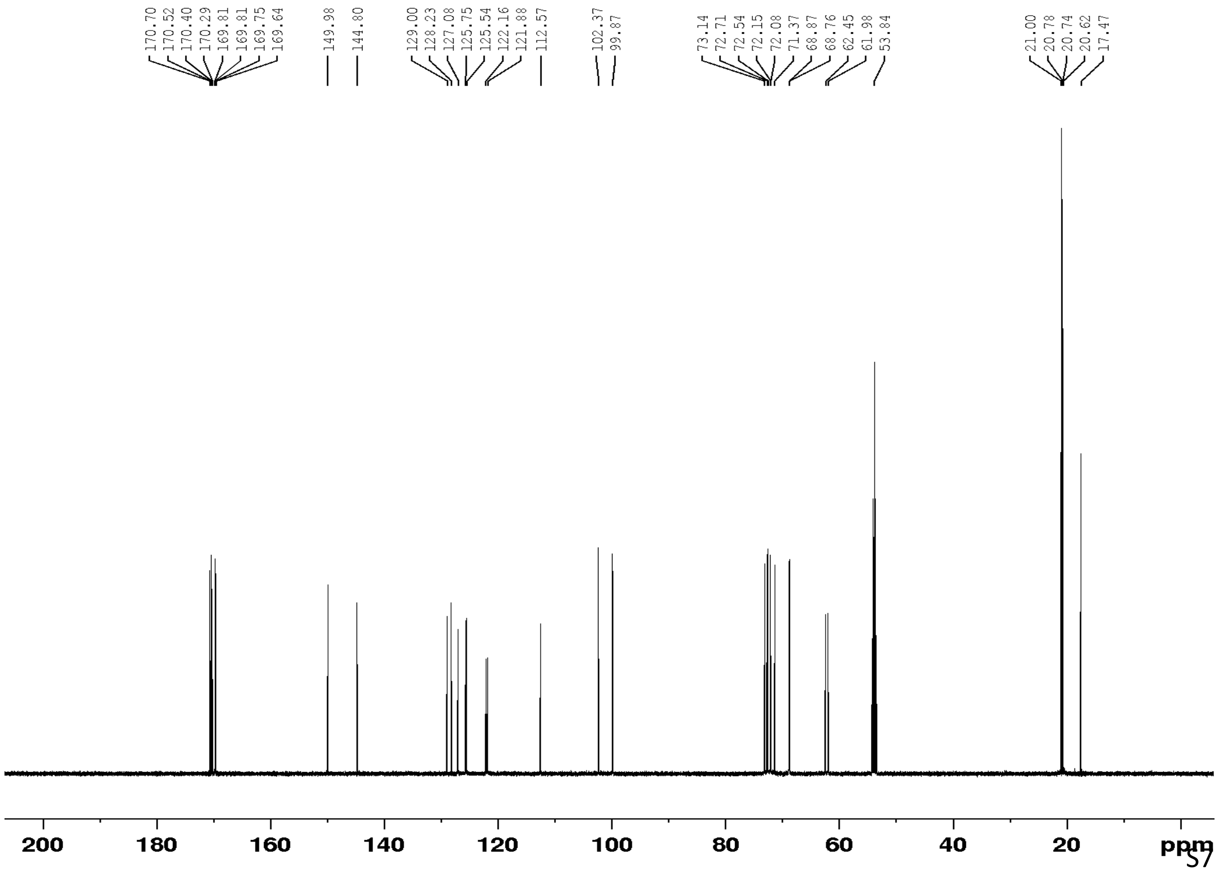

## $^1$H NMR (600 MHz, $CD_2Cl_2$) of 1d

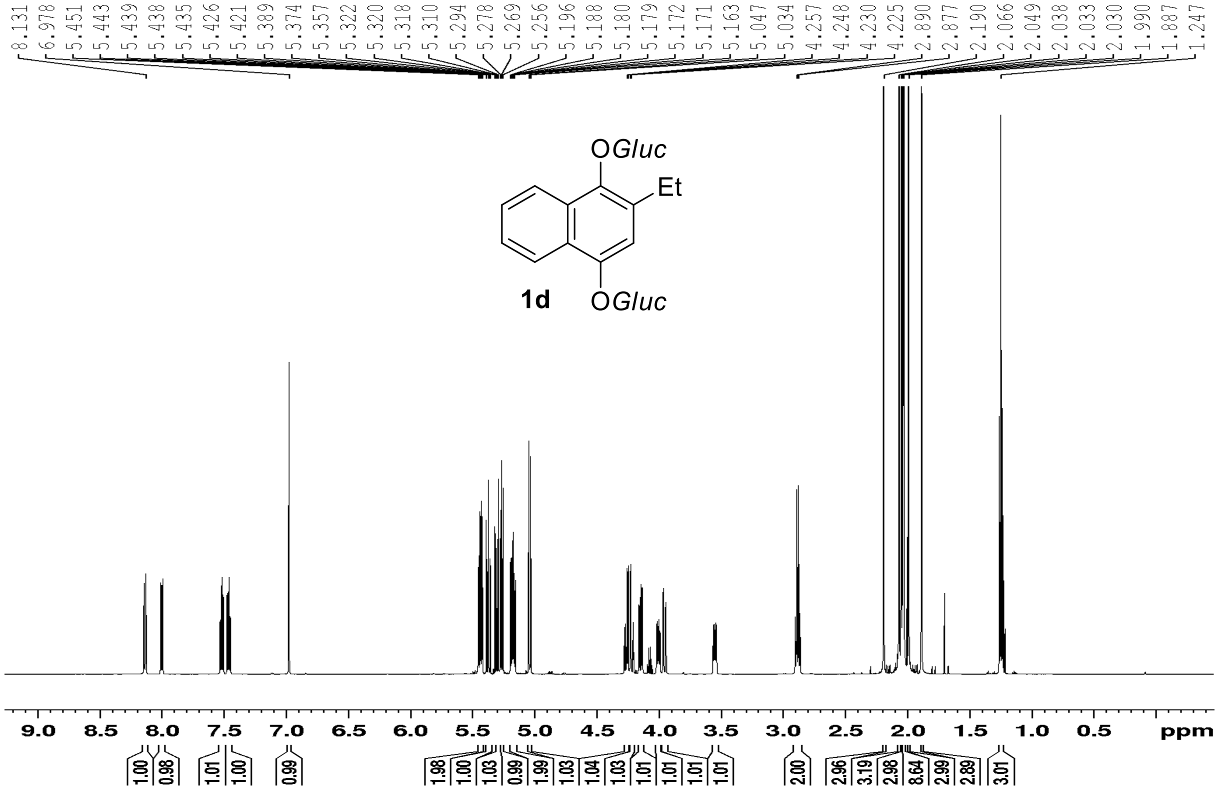


## $^{13}$C NMR (150 MHz, $CD_2Cl_2$) of 1d

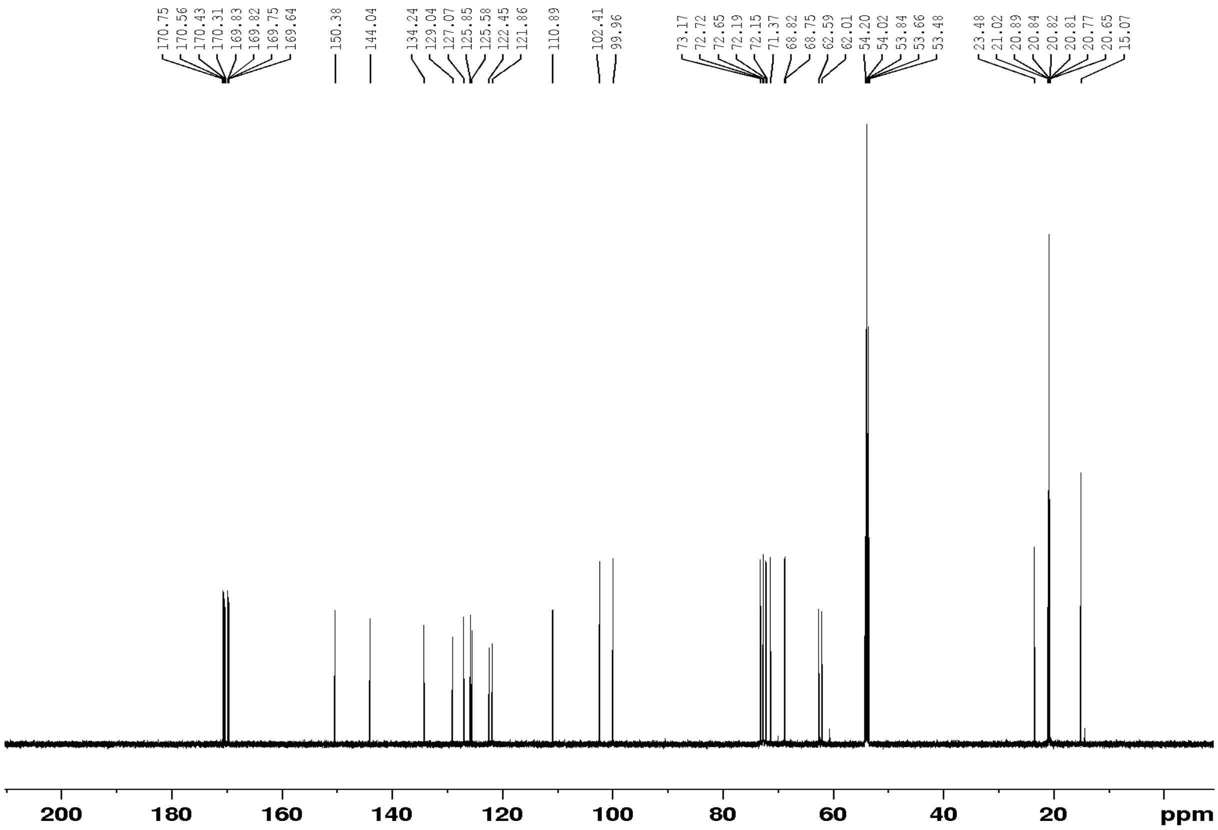

## $^1H$ NMR (600 MHz, $CD_2Cl_2$) of 1e

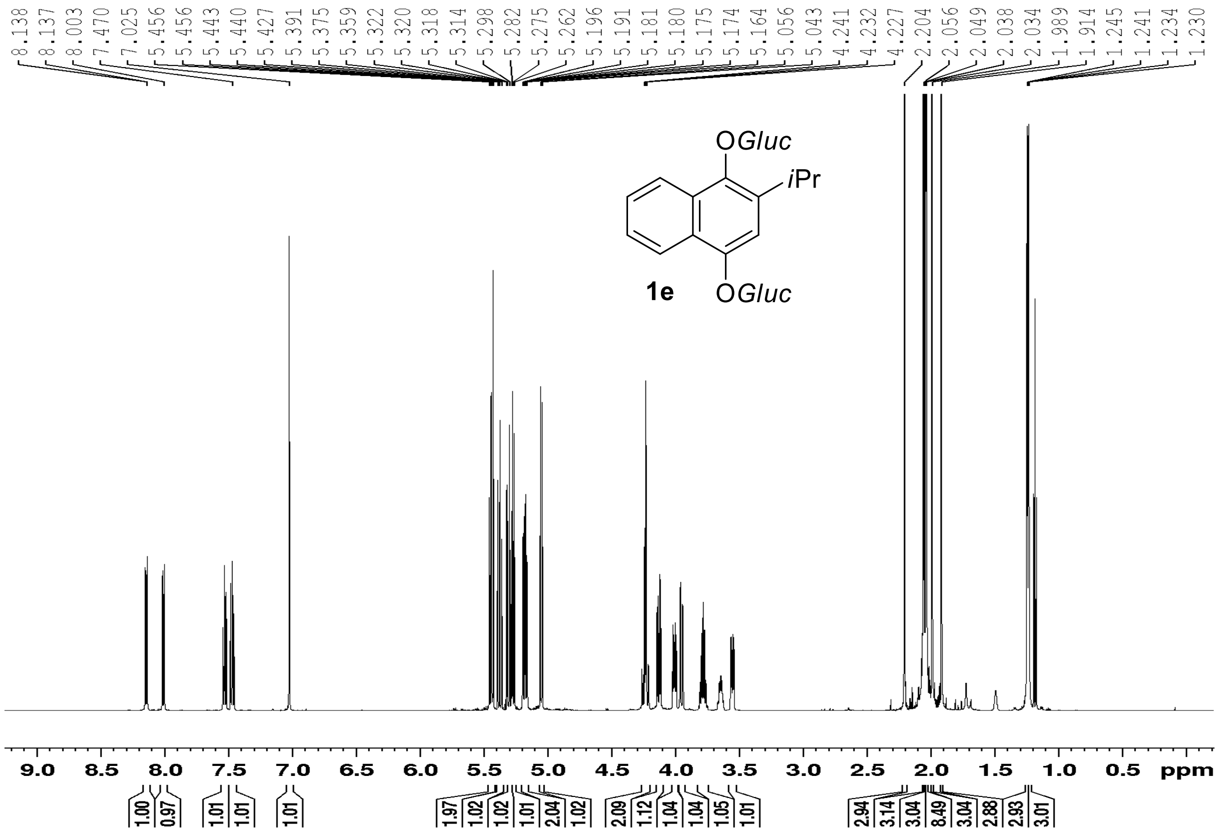


## $^{13}C$ NMR (150 MHz, $CD_2Cl_2$) of 1e

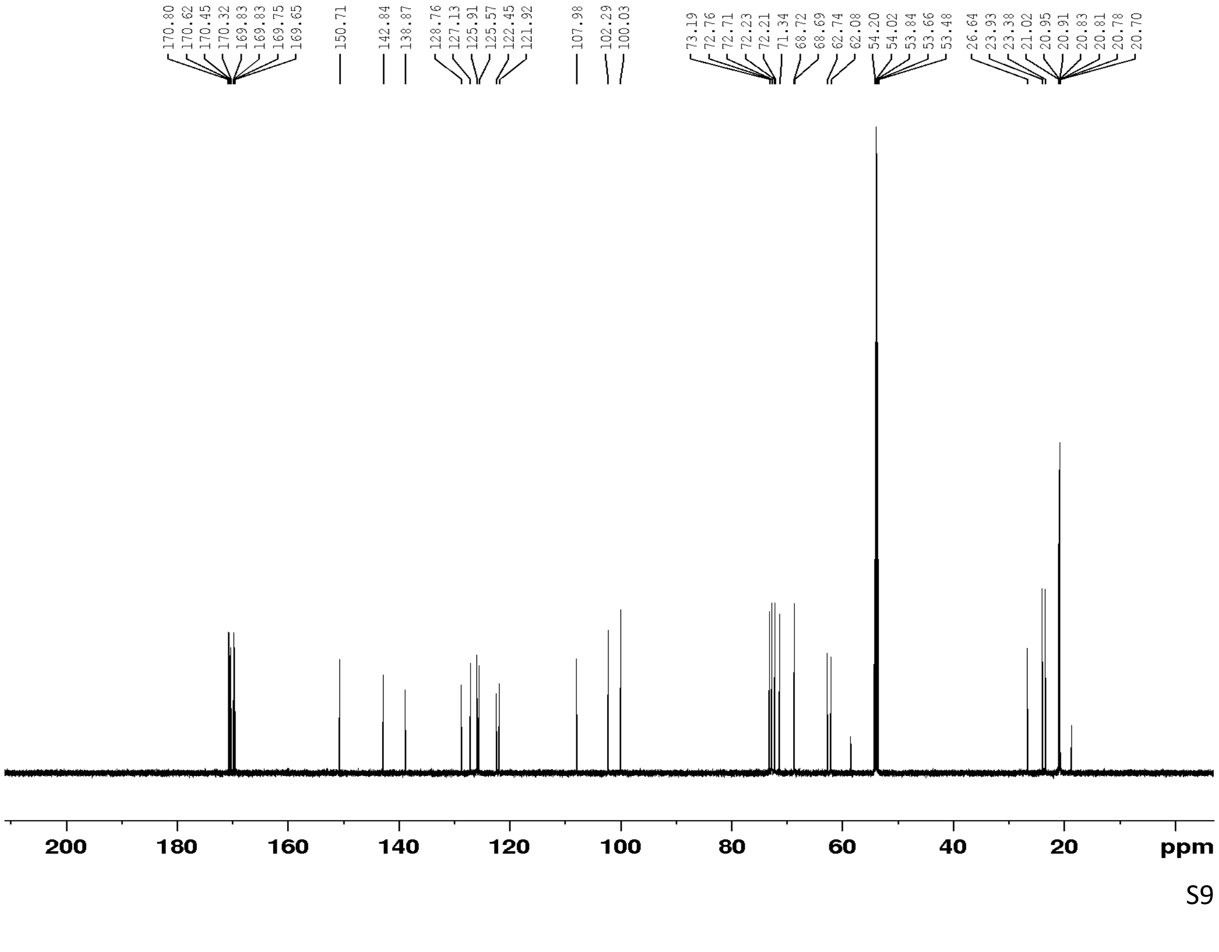

## $^{1}H$ NMR (600 MHz, $CD_2Cl_2$) of 1f

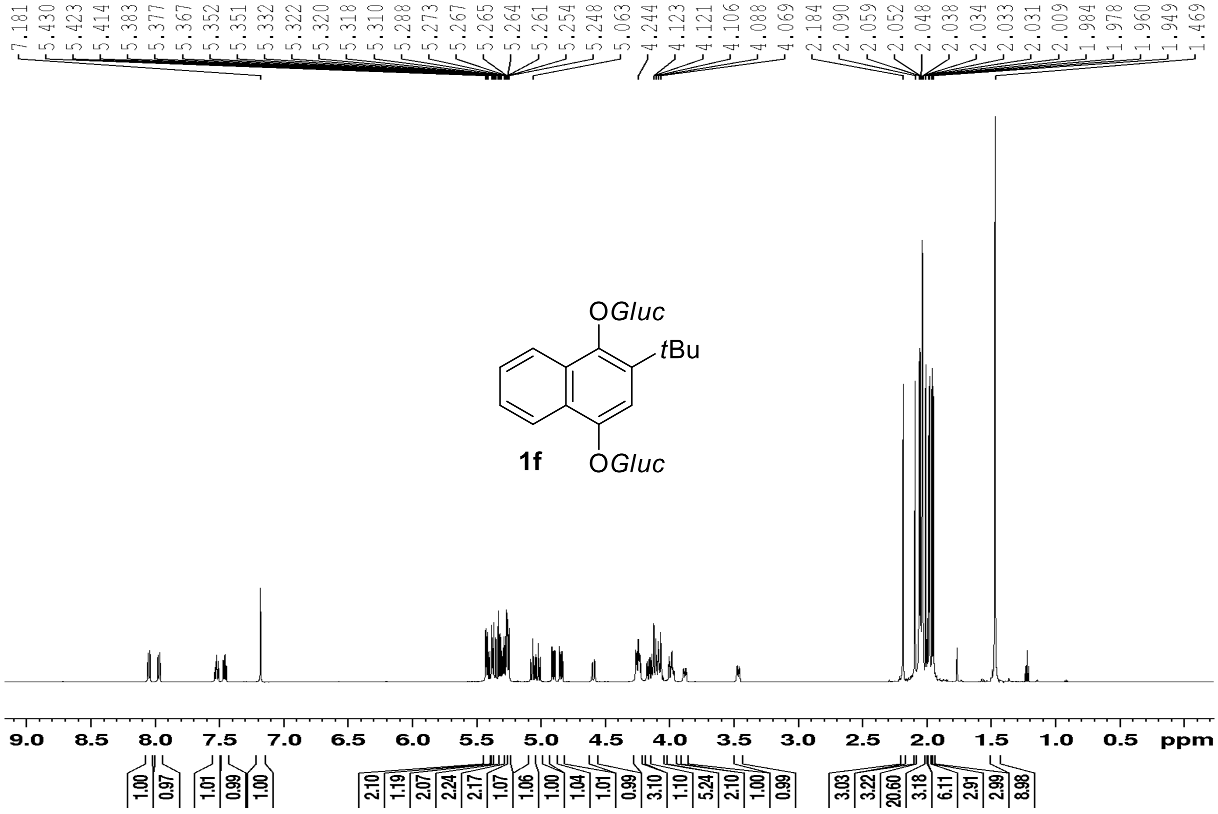


## $^{13}C$ NMR (150 MHz, $CD_2Cl_2$) of 1f

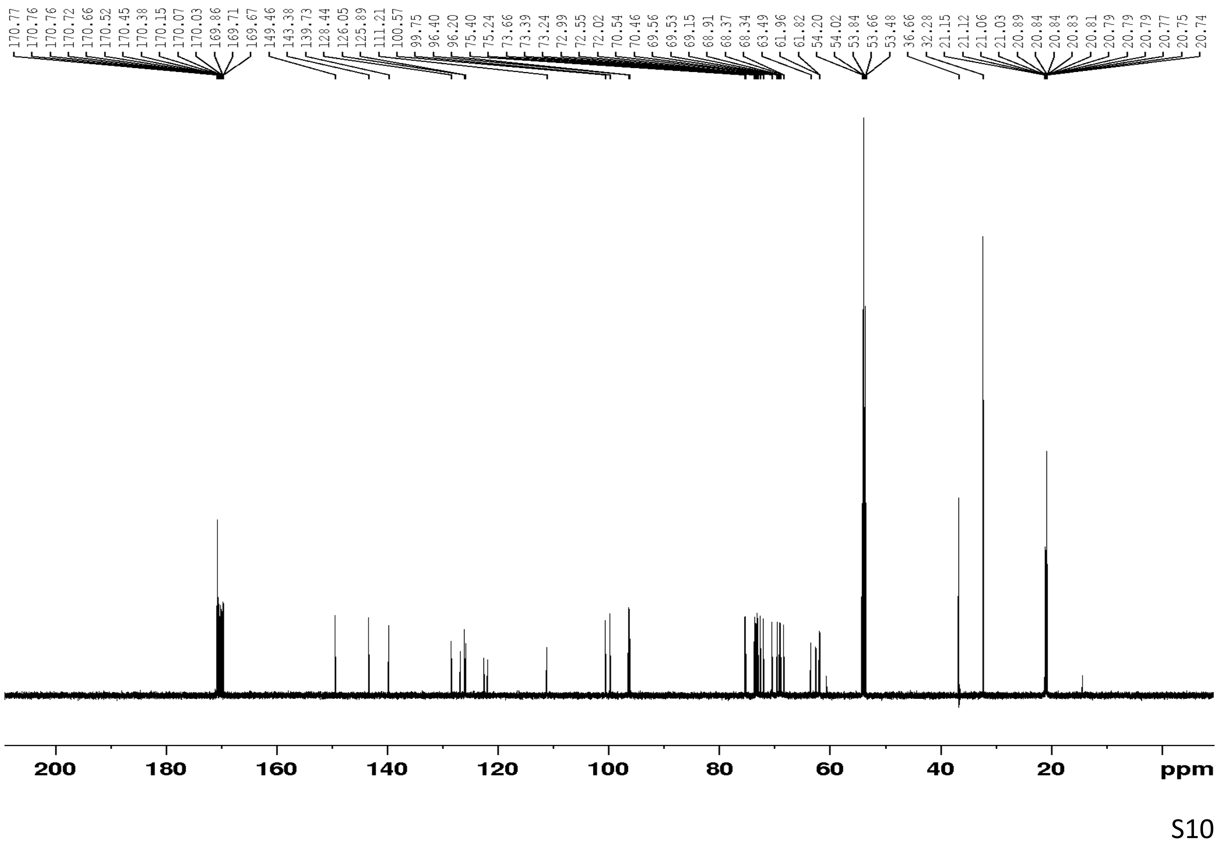

## 3. NMR Spectra of endoperoxides 2

### $^{1}H$ NMR (500 MHz, $CD_2Cl_2$, 195 K) of 2b

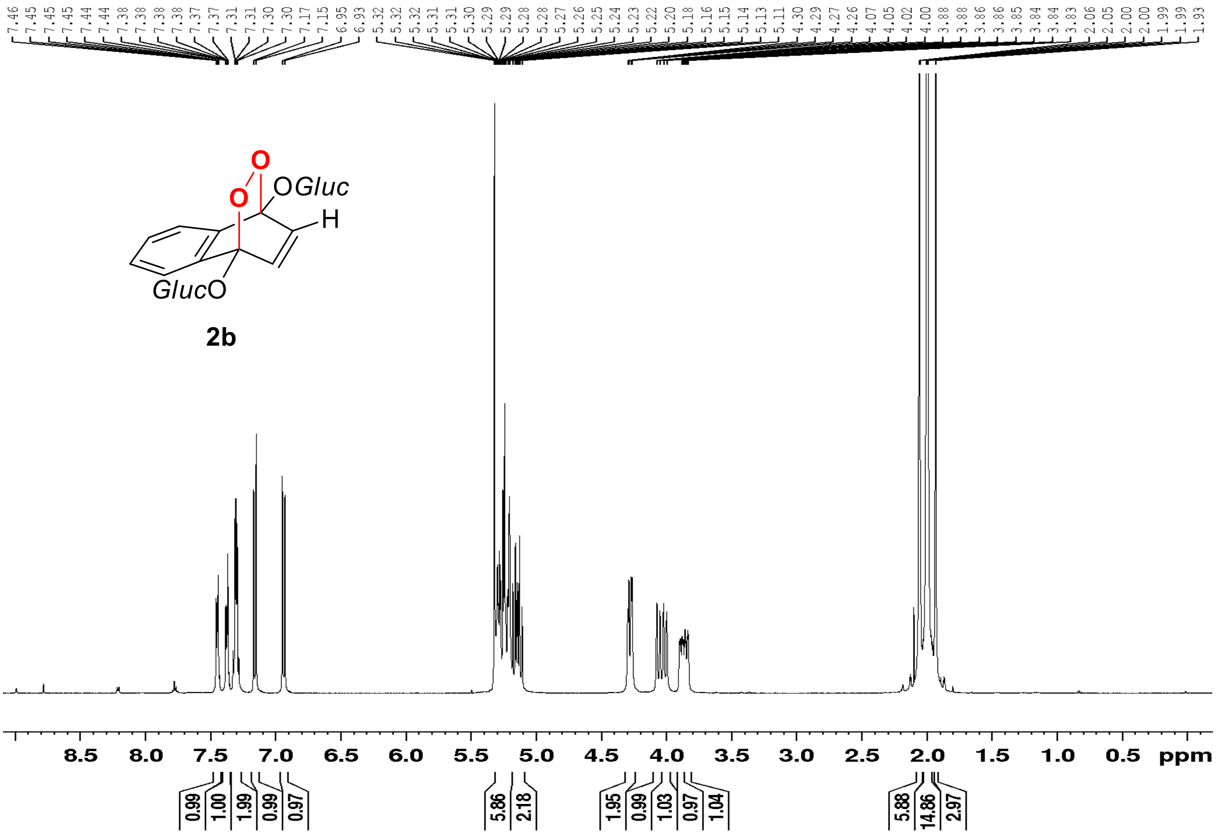


### $^{13}C$ NMR (125 MHz, $CD_2Cl_2$, 195 K) of 2b

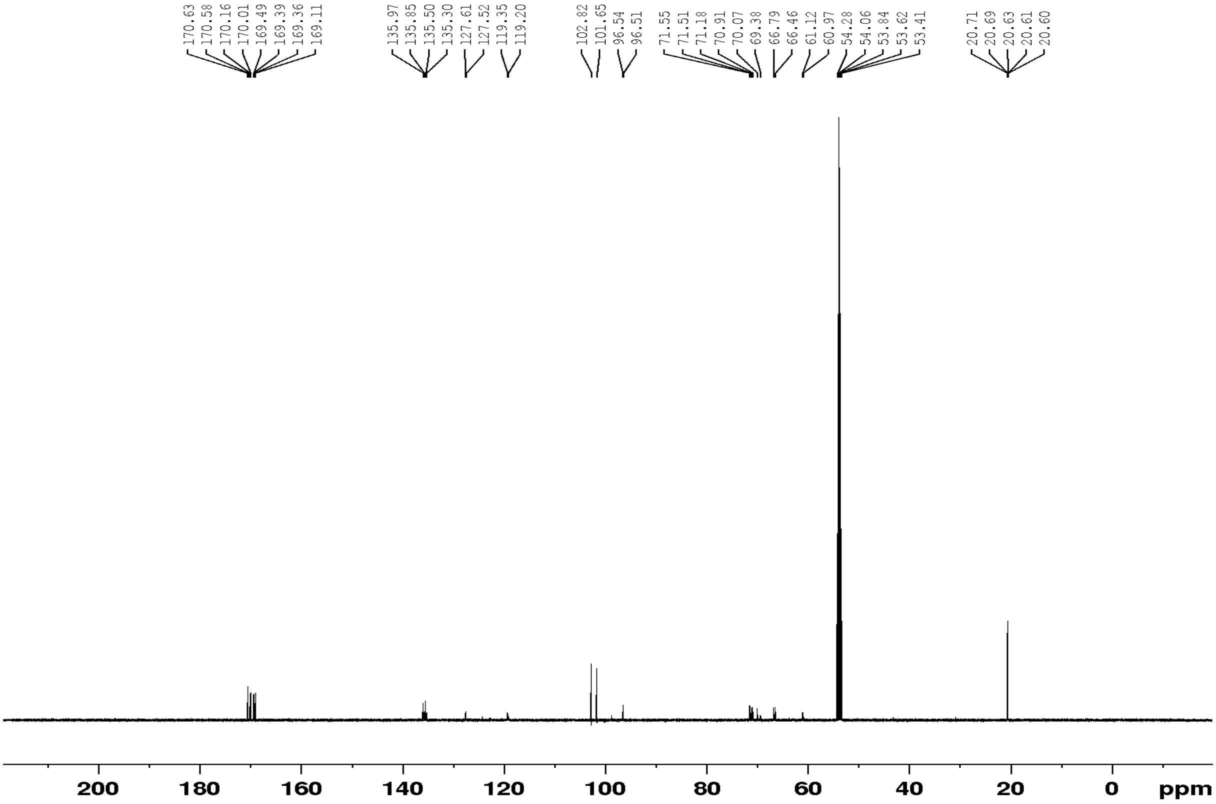

## $^{1}H$ NMR (500 MHz, $CD_2Cl_2$, 195 K) of 2c

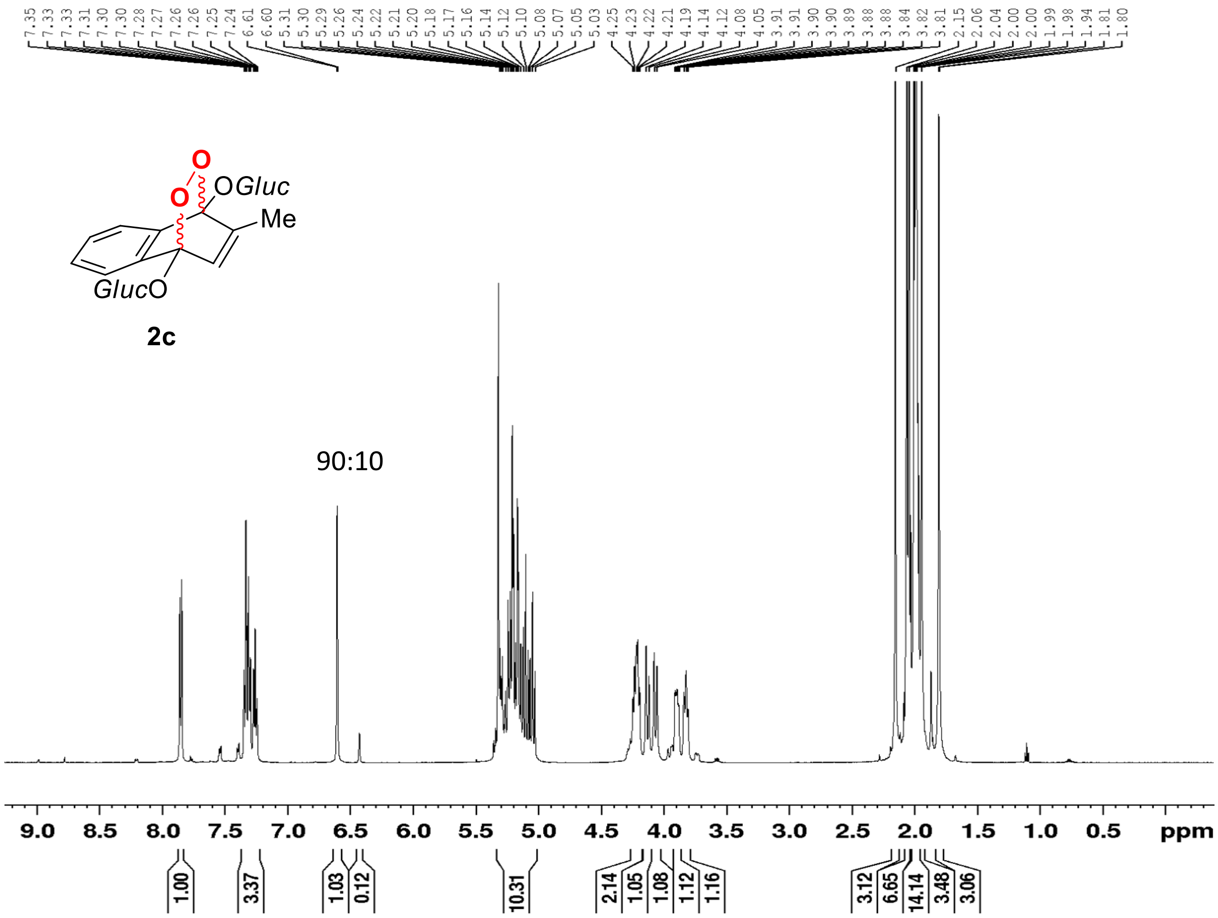


## $^{13}C$ NMR (125 MHz, $CD_2Cl_2$, 195 K) of 2c

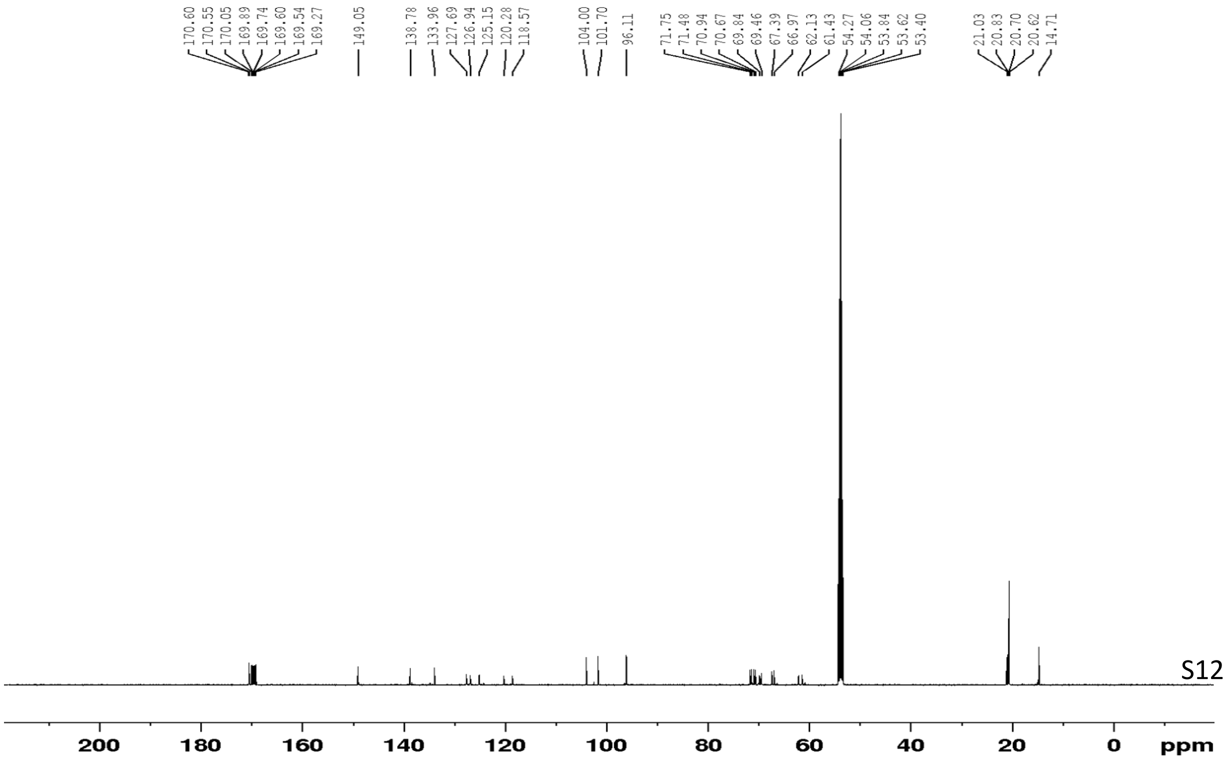

## $^1$H NMR (500 MHz, $CD_2Cl_2$, 253 K) of 2d

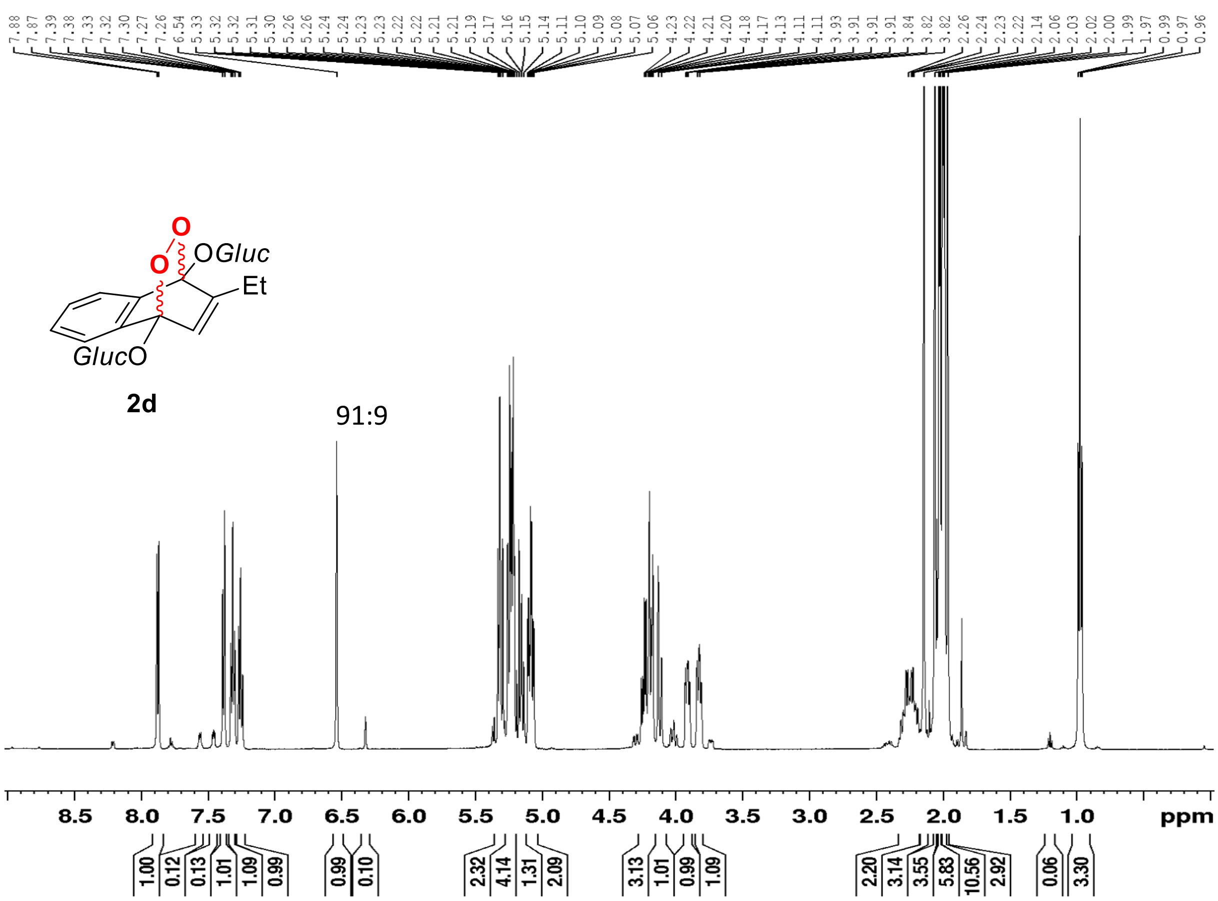


## $^{13}$C NMR (125 MHz, $CD_2Cl_2$, 253 K) of 2d

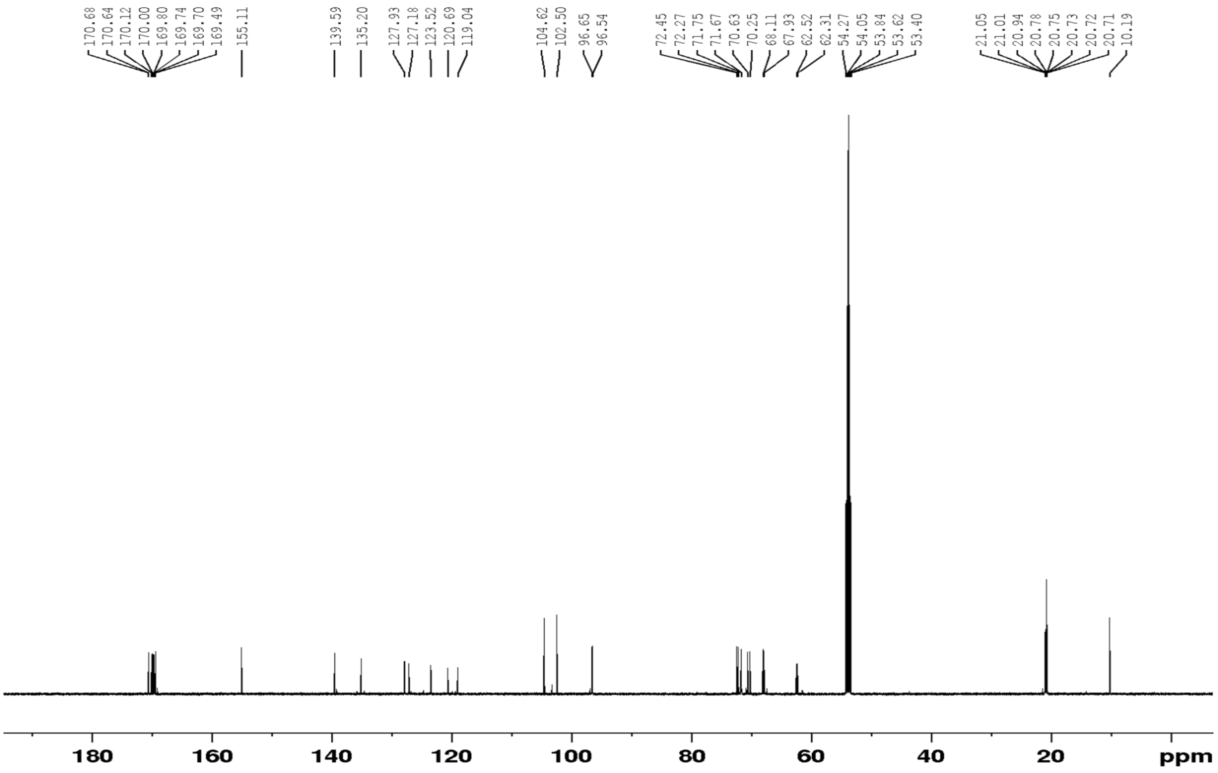

## $^{1}H$ NMR (500 MHz, $CD_2Cl_2$, 253 K) of 2e

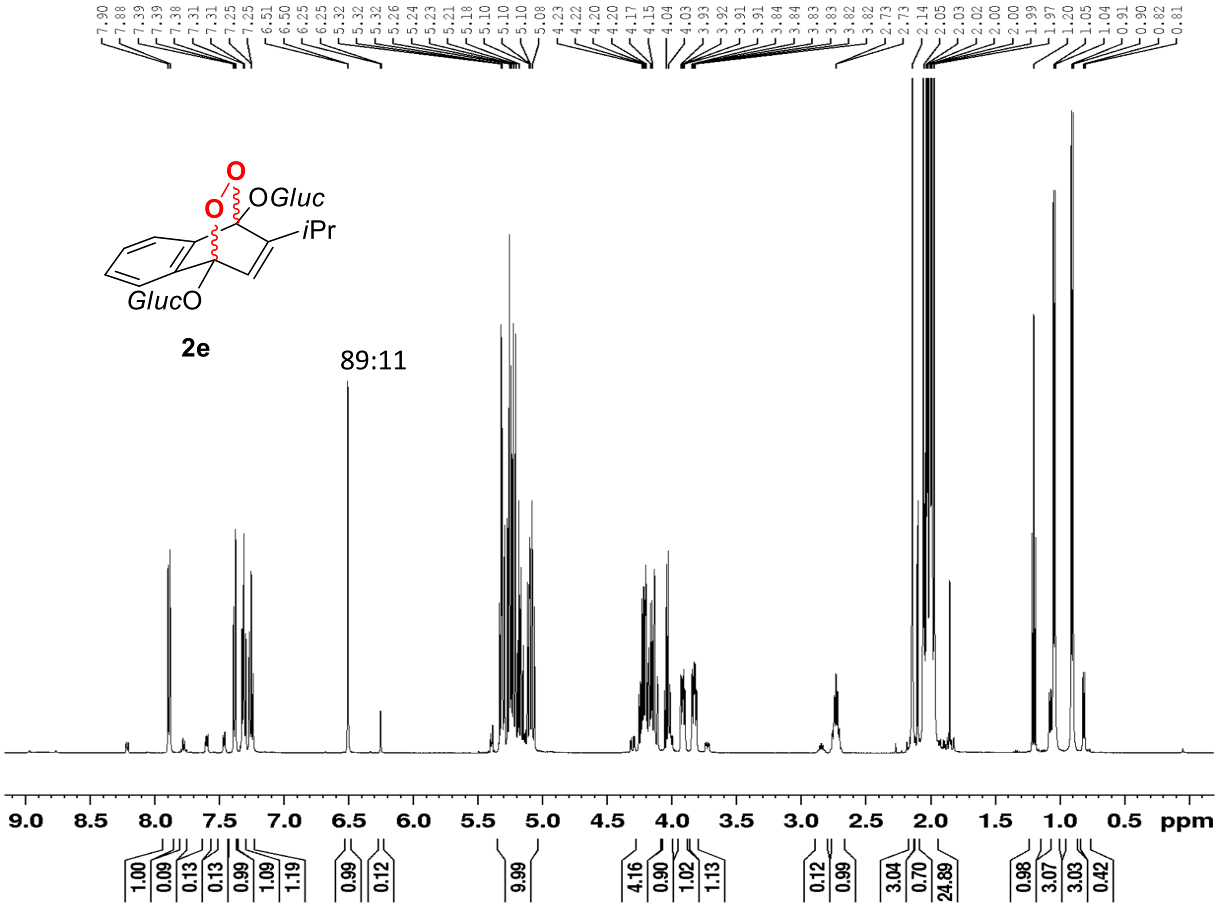


## $^{13}C$ NMR (125 MHz, $CD_2Cl_2$, 253 K) of 2e

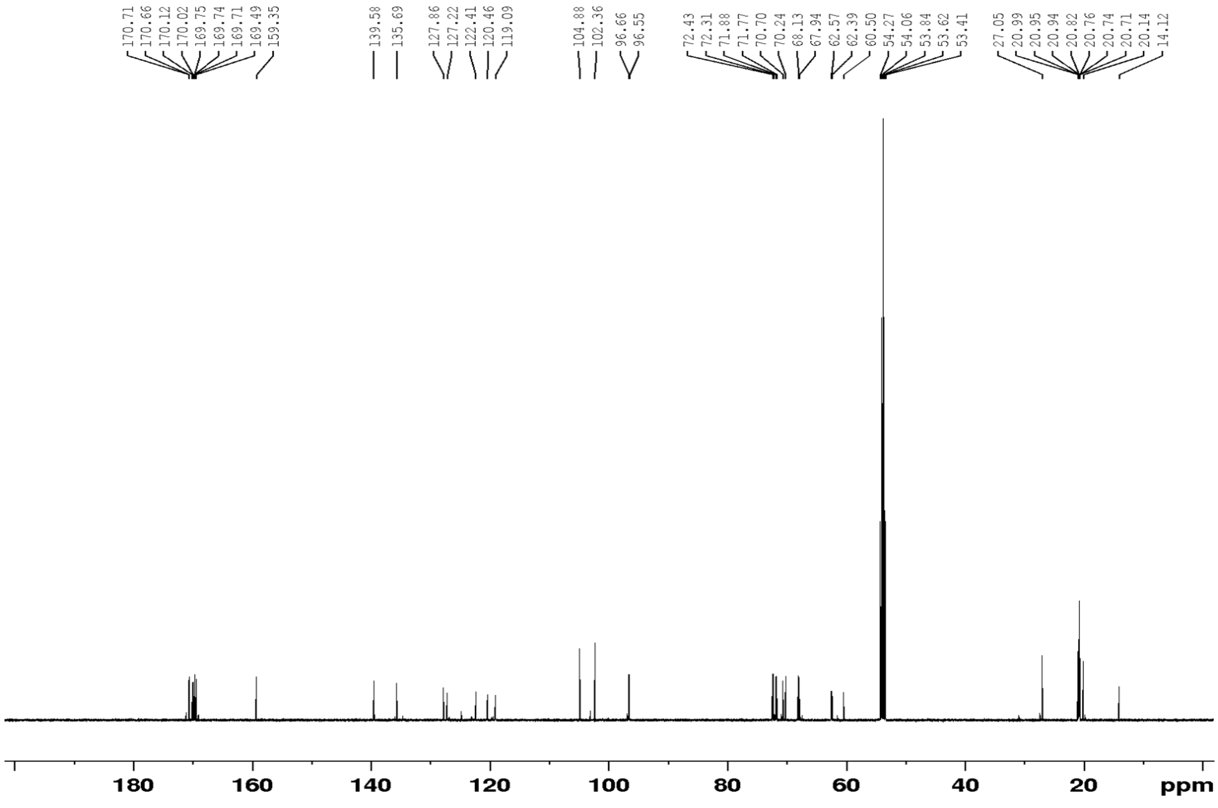

## $^{1}H$ NMR (600 MHz, $CD_2Cl_2$, 298 K) of the crude product of 2f

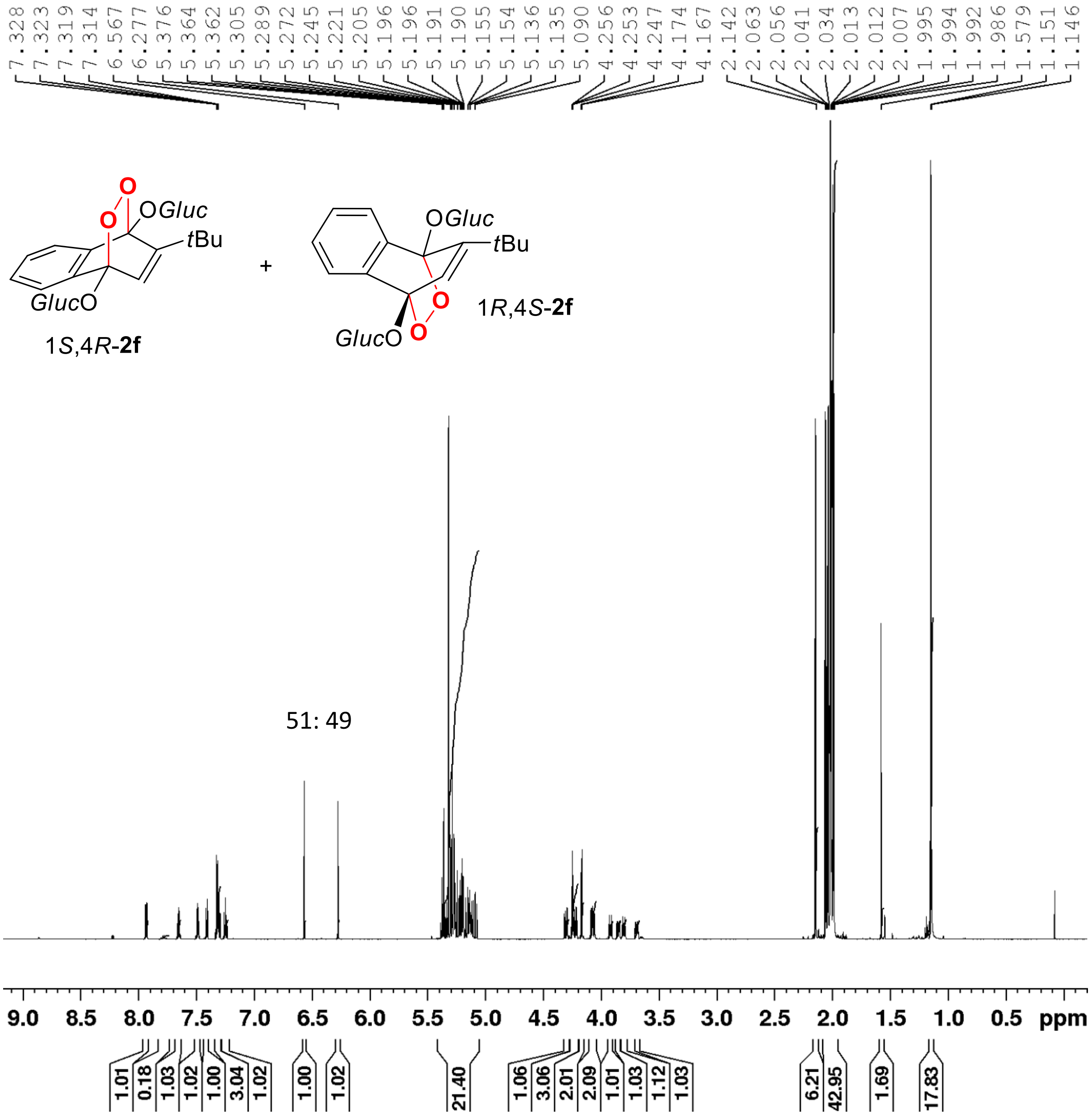

## $^{1}H$ NMR (600 MHz, $CD_2Cl_2$, 298 K) of 1*S*,4*R*-2f

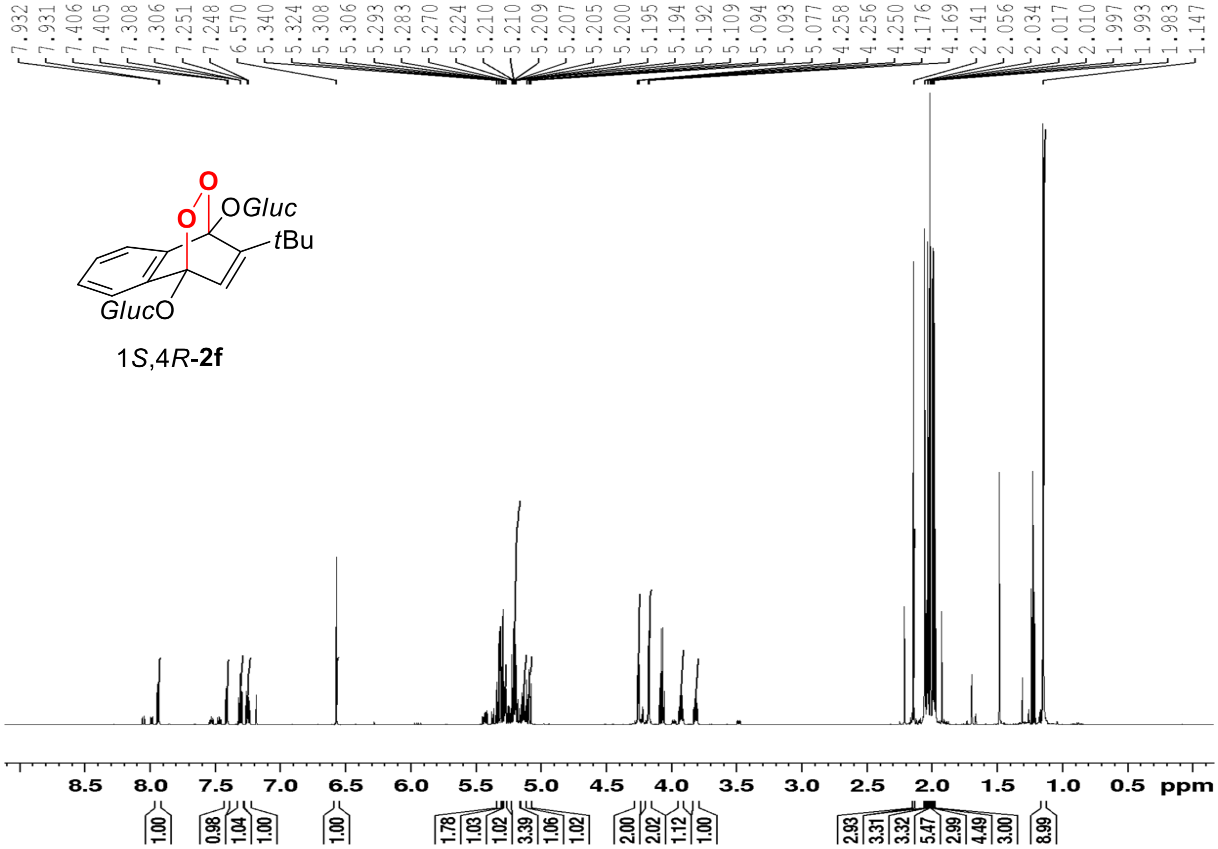


## $^{13}C$ NMR (150 MHz, $CD_2Cl_2$) of 1*S*,4*R*-2f

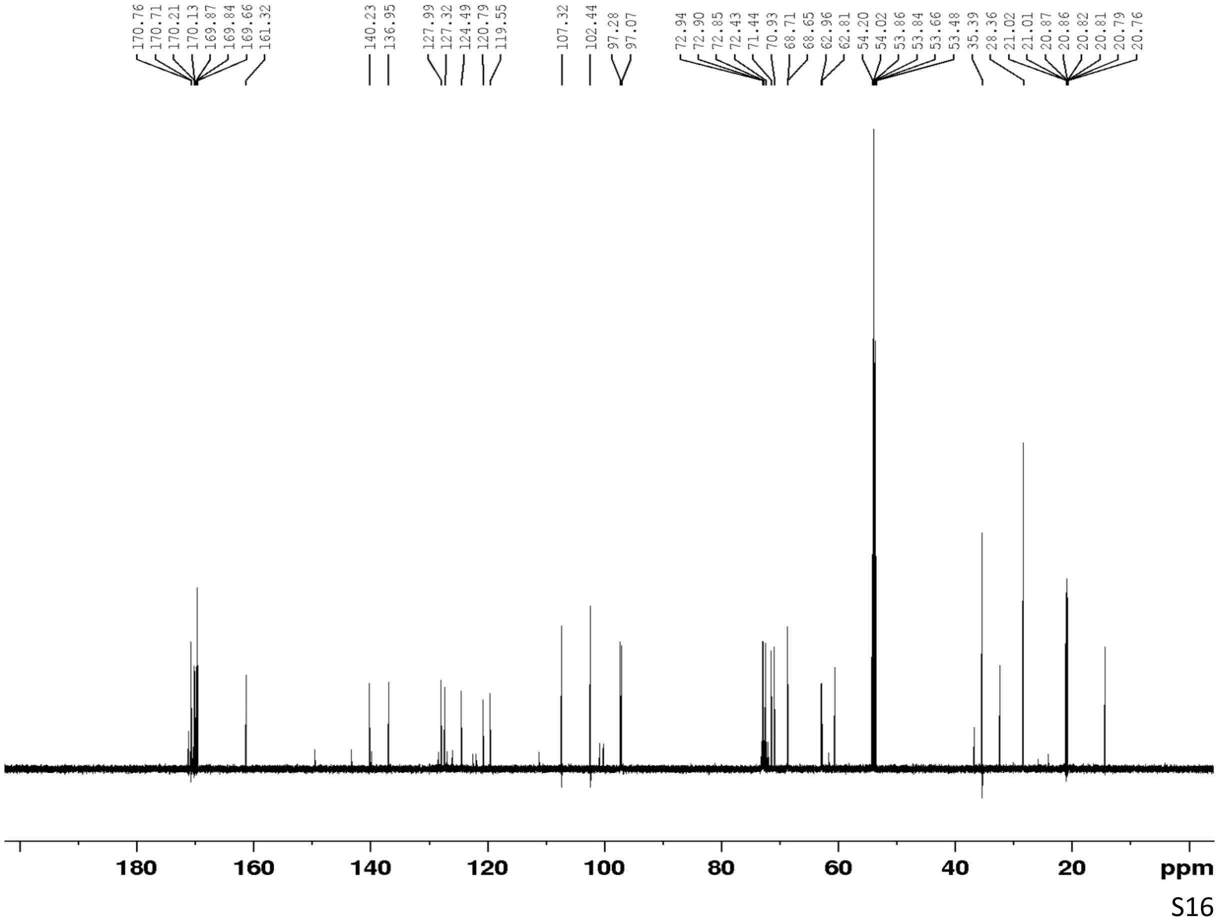

## $^{1}H$ NMR (600 MHz, $CD_2Cl_2$, 298 K) of 1*R*,4*S*-2f

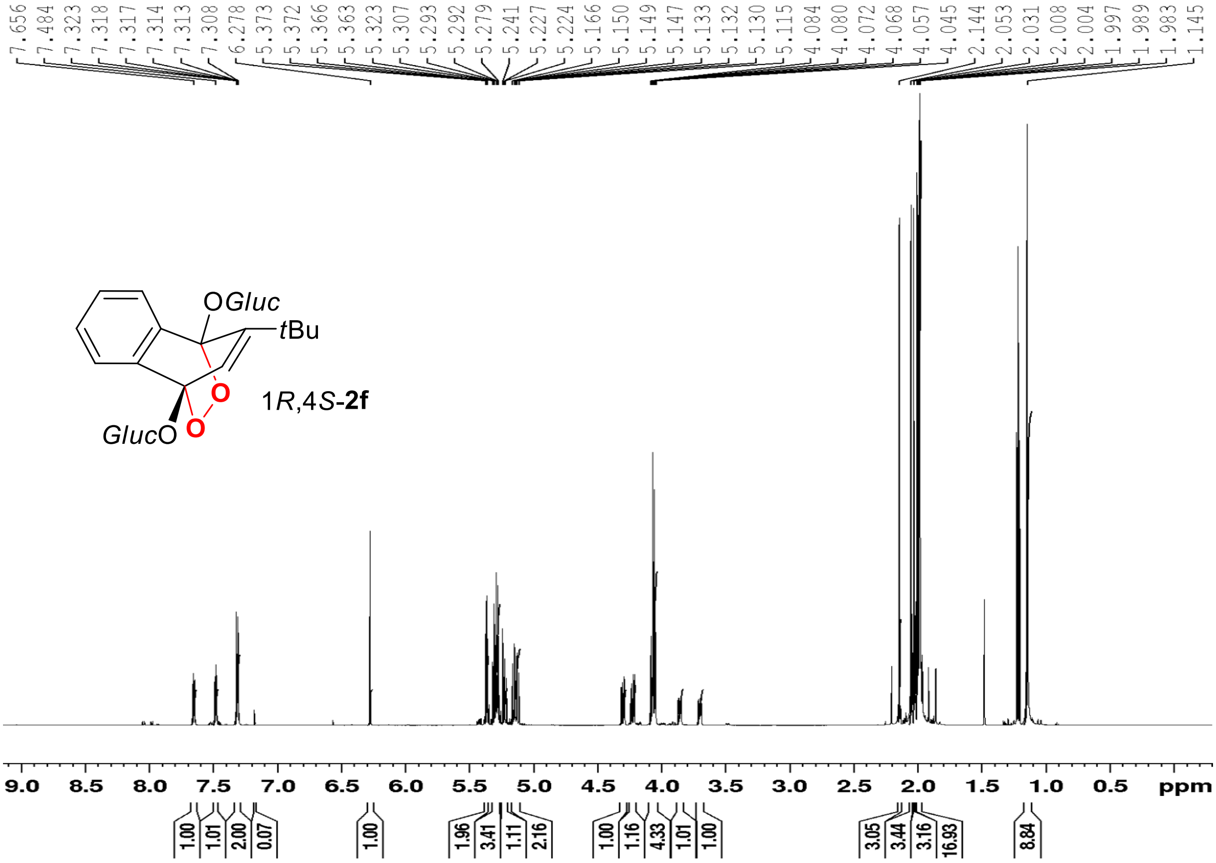


## $^{13}C$ NMR (150 MHz, $CD_2Cl_2$) of 1*R*,4*S*-2f

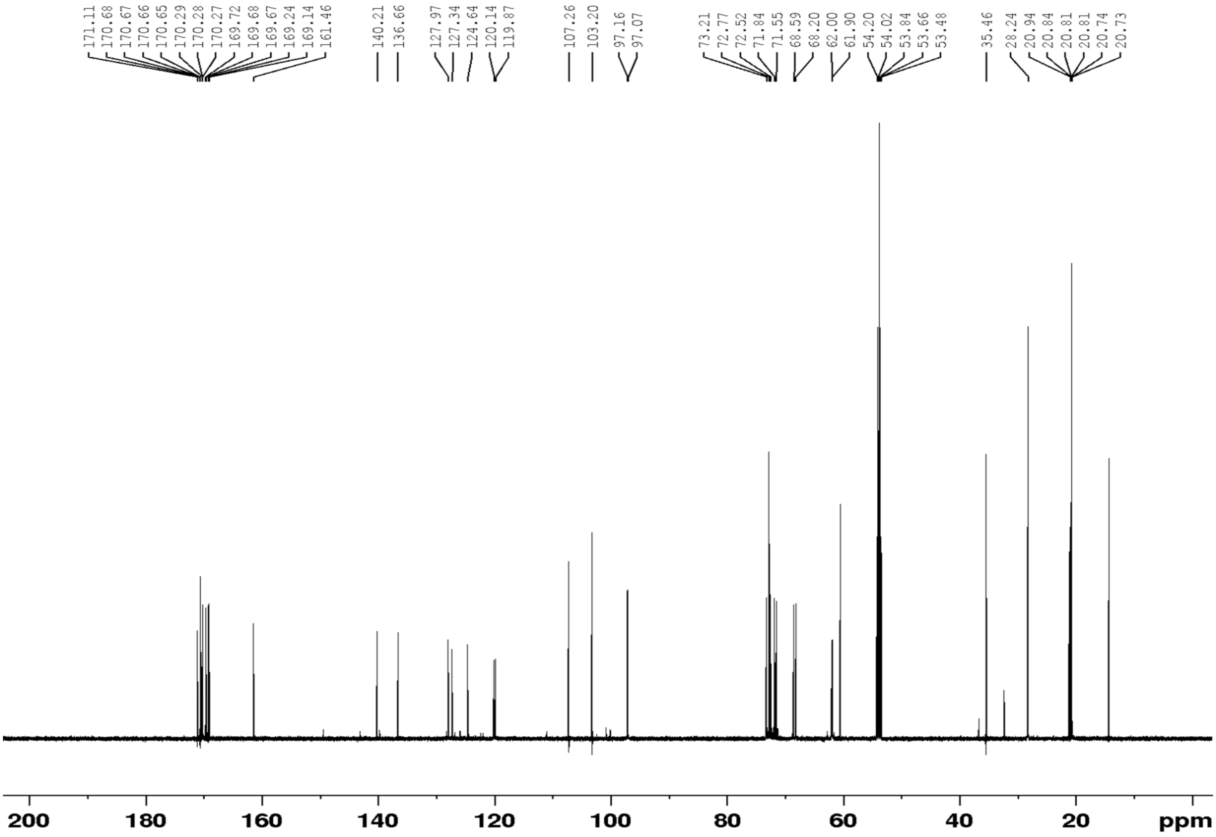

## 4. NOESY (400 MHz, $CD_2Cl_2$) spectrum of endoperoxide 1*S*,4*R*-2f

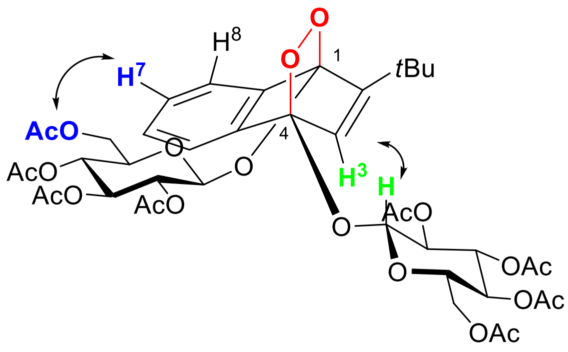


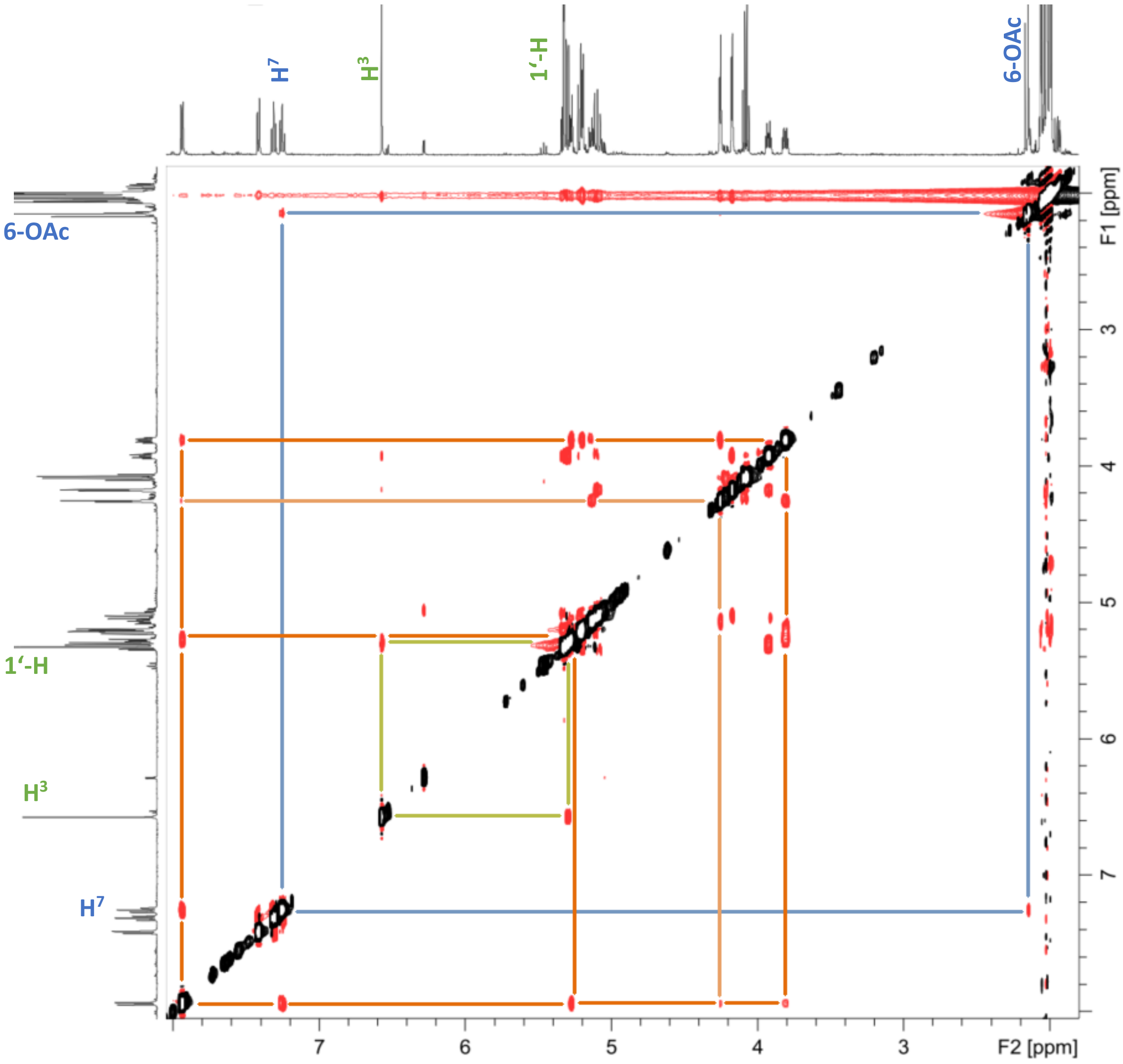

## NOESY (400 MHz, $CD_2Cl_2$) spectrum of endoperoxide 1*R*,4*S*-2f

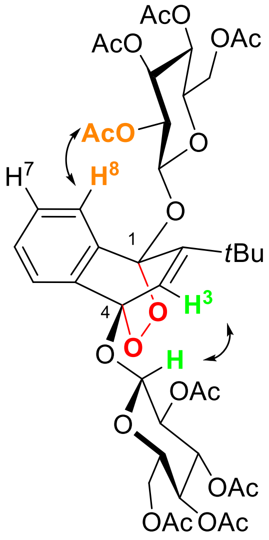


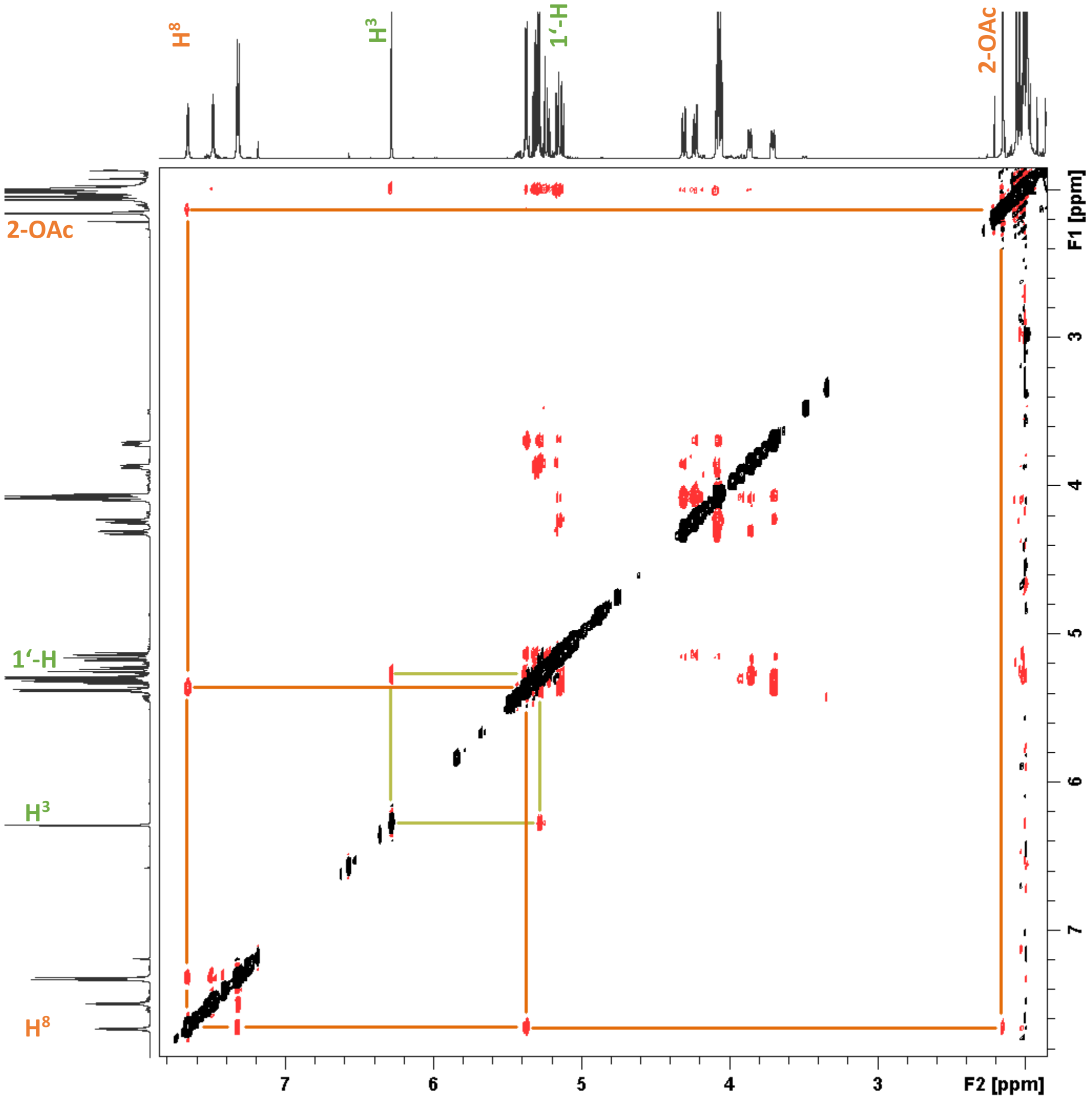

## 5. NMR Spectra of epoxides 7 and 8

### $^1$H NMR (400 MHz, $CDCl_3$)

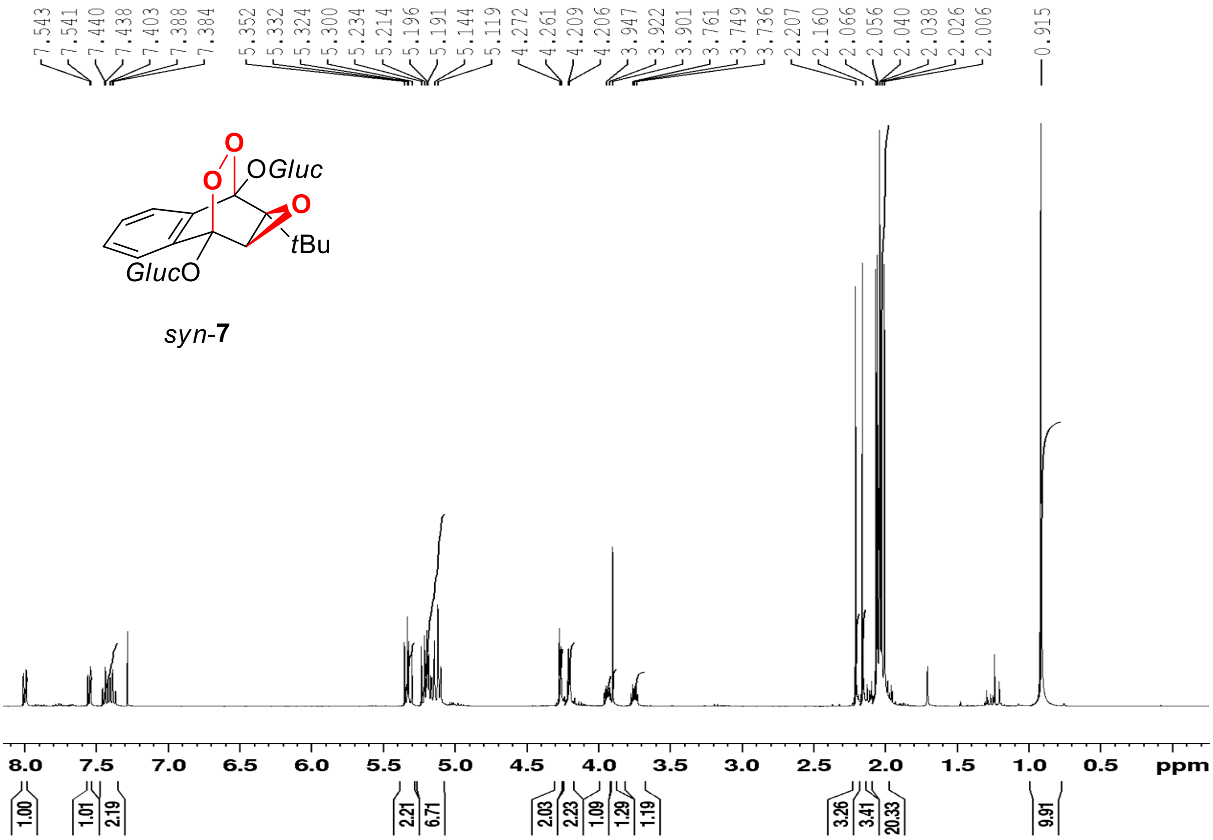


### $^{13}$C NMR (100 MHz, $CDCl_3$)

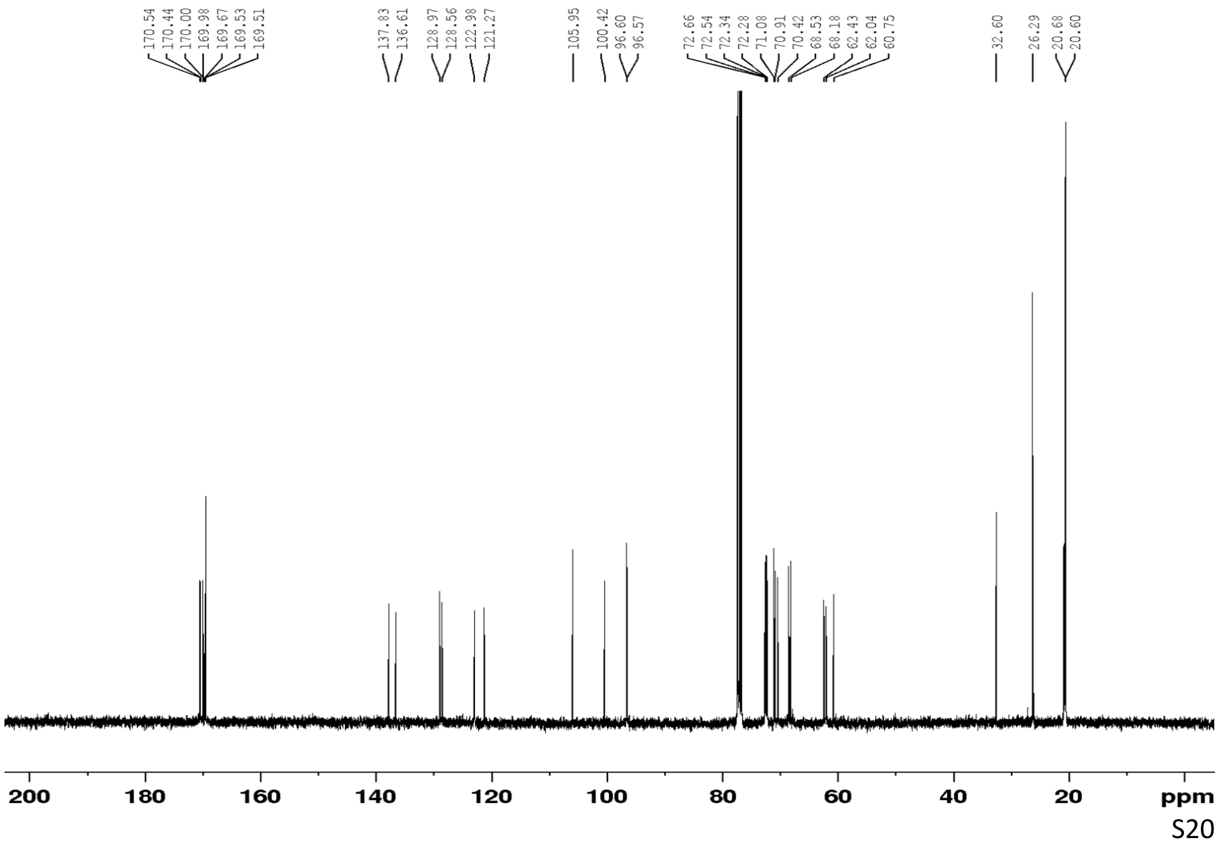

# $^{1}H$ NMR (400 MHz, $CDCl_3$)

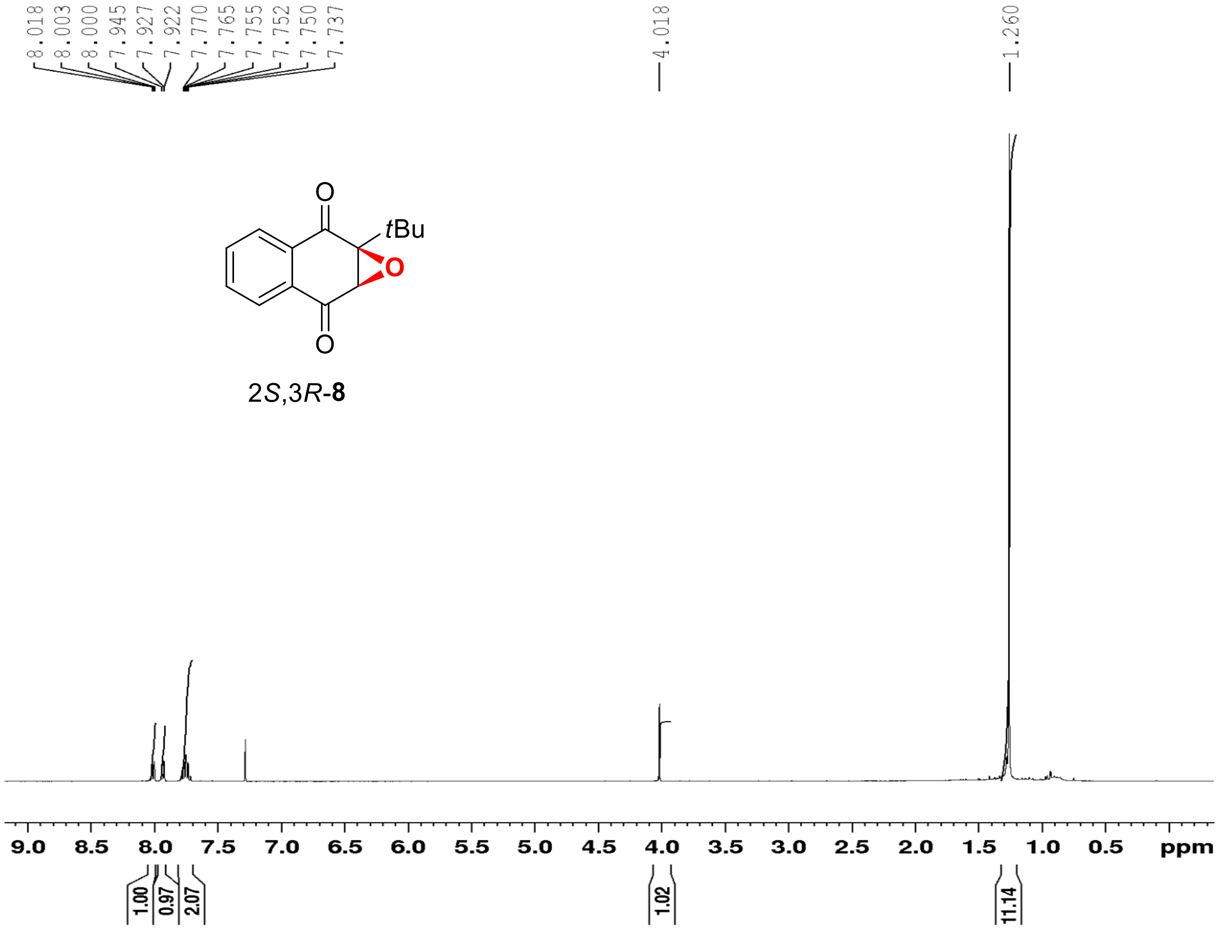


2*S*,3*R*-**8**

# $^{13}C$ NMR (100 MHz, $CDCl_3$)

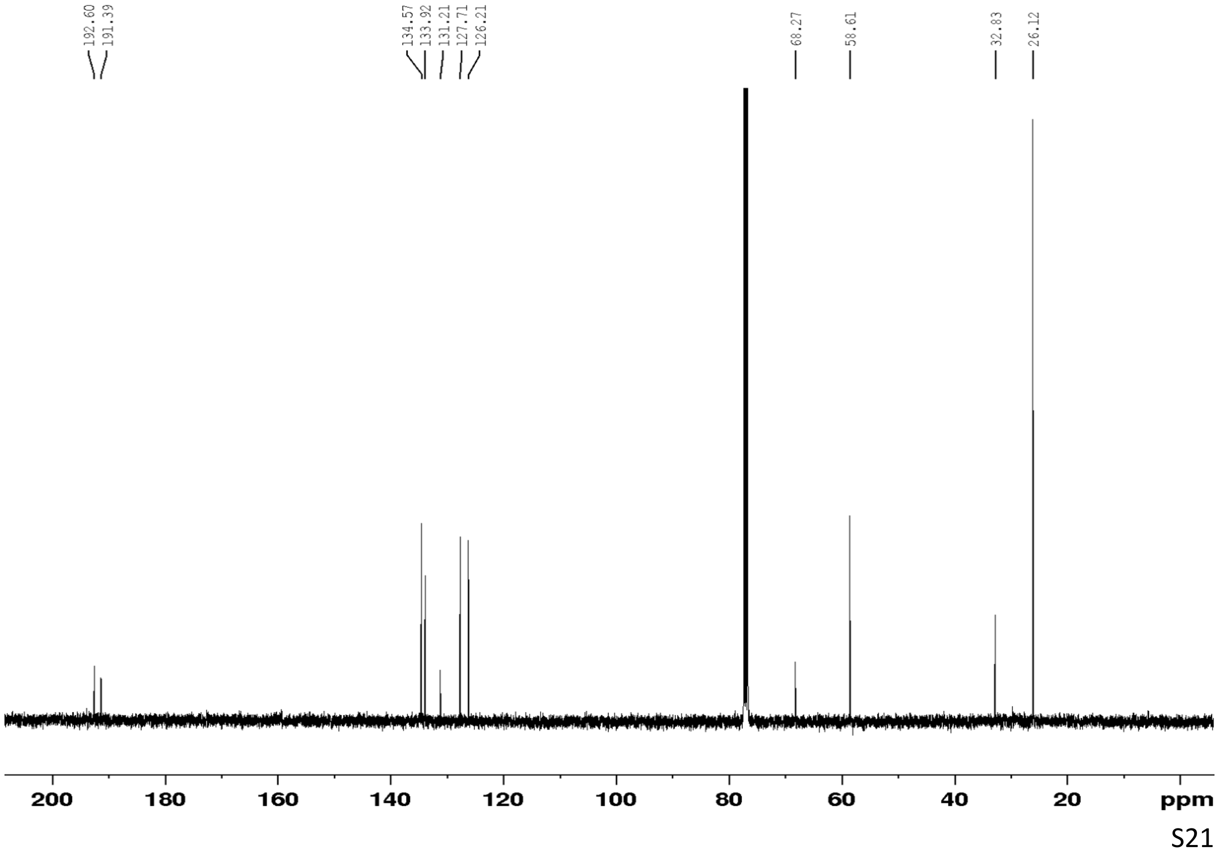

## Theoretical calculations

The structures of the EPOs **2f** were calculated using the Gaussian 09 software. A 6-31G* basis set and the B3LYP functional were applied for the optimization of the ground state structures. This calculation was performed with a frequency analysis revealing no imaginary vibration modes.

Minimum distances between NOESY effective hydrogen pairs were determined from these optimized structures.

| | 1*S*,4*R*-**2f** | 1R,4S-**2f** |
|---|---|---|
| Total energy (hartree) | -3286.6850219 | -3286.6793433 |
| Distance (Å) | 2.402 ($H^7$, 6OAc) | 2.429 ($H^8$, 2OAc) |
| | 2.755 ($H^3$, H1) | 2.345($H^3$, H1) |

Coordinates for 1*S*,4*R*-**2f**

| | | | |
|---|---|---|---|
| C | 2.75900 | 2.35274 | 2.45531 |
| C | 3.10491 | 0.86900 | 2.65257 |
| C | 2.72396 | 0.17494 | 1.56939 |
| H | 2.83093 | -0.89381 | 1.43746 |
| C | 2.11560 | 0.98903 | 0.45385 |
| C | 3.49364 | 2.42267 | -1.20963 |
| H | 3.16424 | 1.83792 | -2.06192 |
| C | 4.37220 | 3.50330 | -1.36055 |
| H | 4.74448 | 3.75893 | -2.34876 |

| | | | |
|---|---|---|---|
| C | 4.77708 | 4.24221 | -0.24993 |
| H | 5.47050 | 5.06972 | -0.37359 |
| C | 4.30209 | 3.93541 | 1.03332 |
| H | 4.62735 | 4.51562 | 1.88949 |
| C | 3.42255 | 2.86801 | 1.18262 |
| C | 3.04785 | 2.10745 | 0.06485 |
| C | 2.55958 | 4.46510 | 3.62016 |
| H | 2.16327 | 4.76692 | 2.64245 |
| C | 1.53816 | 4.73680 | 4.73343 |
| H | 1.88858 | 4.26703 | 5.65552 |
| C | 1.32836 | 6.23985 | 4.94014 |
| H | 0.73249 | 6.65380 | 4.12101 |
| C | 2.65300 | 7.01242 | 5.03105 |
| H | 3.11029 | 6.85114 | 6.01152 |
| C | 3.60312 | 6.57942 | 3.89995 |
| H | 3.17663 | 6.89685 | 2.93530 |
| C | -0.18852 | 3.10289 | 5.05213 |
| C | -1.45077 | 2.58454 | 4.41483 |
| H | -1.91811 | 1.85139 | 5.07319 |
| H | -1.18706 | 2.11323 | 3.46175 |
| H | -2.14317 | 3.40369 | 4.20066 |
| C | -0.39353 | 7.31213 | 6.23217 |
| C | -0.99344 | 7.33760 | 7.61390 |
| H | -1.17586 | 6.32392 | 7.98032 |
| H | -1.91834 | 7.91553 | 7.60096 |
| H | -0.27483 | 7.81302 | 8.29136 |
| C | 2.23420 | 9.20411 | 5.94095 |
| C | 1.85327 | 10.60121 | 5.52714 |
| H | 0.80987 | 10.58352 | 5.19195 |
| H | 2.46752 | 10.94698 | 4.69145 |

| | | | |
|---|---|---|---|
| H | 1.95107 | 11.27441 | 6.37958 |
| C | 4.98482 | 7.18630 | 4.05403 |
| H | 4.91568 | 8.26605 | 4.20631 |
| H | 5.50537 | 6.74363 | 4.90883 |
| C | 6.97670 | 7.41668 | 2.80115 |
| C | 7.65537 | 7.05249 | 1.50269 |
| H | 8.65701 | 7.48290 | 1.48507 |
| H | 7.07421 | 7.42857 | 0.65415 |
| H | 7.71760 | 5.96368 | 1.40340 |
| C | 3.77861 | 0.28829 | 3.89469 |
| C | 4.11317 | -1.19963 | 3.66736 |
| H | 4.77683 | -1.34087 | 2.80672 |
| H | 3.21058 | -1.80120 | 3.51034 |
| H | 4.62191 | -1.59675 | 4.55244 |
| C | 2.84221 | 0.38857 | 5.12278 |
| H | 1.91215 | -0.16406 | 4.94849 |
| H | 2.57829 | 1.41801 | 5.36491 |
| H | 3.33794 | -0.05288 | 5.99618 |
| C | 0.76136 | -0.75685 | -0.46104 |
| H | 0.25049 | -0.64680 | 0.50598 |
| C | -0.25482 | -0.70845 | -1.60983 |
| H | 0.28538 | -0.64529 | -2.55773 |
| C | -1.16276 | -1.93603 | -1.58241 |
| H | -1.85960 | -1.87991 | -0.74083 |
| C | -0.32557 | -3.21304 | -1.49005 |
| H | 0.22586 | -3.36678 | -2.41893 |
| C | 0.65588 | -3.11664 | -0.30927 |
| H | 0.08194 | -3.00467 | 0.62703 |
| C | -0.80500 | 1.53609 | -2.23842 |
| C | -1.56774 | 2.73646 | -1.74161 |

| | | | |
|---|---|---|---|
| H | -1.10563 | 3.07223 | -0.80618 |
| H | -2.60721 | 2.47528 | -1.52359 |
| H | -1.51981 | 3.53476 | -2.48300 |
| C | -3.28206 | -1.96622 | -2.71133 |
| C | -3.88489 | -1.99474 | -4.09397 |
| H | -4.97232 | -2.01581 | -4.01674 |
| H | -3.52736 | -2.87522 | -4.63762 |
| H | -3.56642 | -1.11276 | -4.65922 |
| C | -1.32657 | -5.25449 | -2.26237 |
| C | -2.17243 | -6.41228 | -1.80374 |
| H | -2.97780 | -6.08084 | -1.14421 |
| H | -1.52532 | -7.09855 | -1.24508 |
| H | -2.57333 | -6.93674 | -2.67241 |
| C | 1.53776 | -4.34596 | -0.14100 |
| H | 2.33376 | -4.11797 | 0.57370 |
| H | 0.94644 | -5.18738 | 0.22281 |
| C | 1.94826 | -5.97368 | -1.84127 |
| C | 2.56924 | -6.16298 | -3.19982 |
| H | 3.47246 | -5.56007 | -3.31526 |
| H | 1.83649 | -5.84027 | -3.94869 |
| H | 2.78514 | -7.22111 | -3.35701 |
| O | 2.90232 | 3.09689 | 3.62062 |
| O | 0.27557 | 4.18329 | 4.35636 |
| O | 0.36020 | 2.64012 | 6.02429 |
| O | 0.58854 | 6.37217 | 6.16562 |
| O | -0.69942 | 8.03647 | 5.31109 |
| O | 2.40853 | 8.41765 | 4.83789 |
| O | 2.34424 | 8.80220 | 7.07614 |
| O | 5.71566 | 6.91258 | 2.84592 |
| O | 7.46164 | 8.06694 | 3.69860 |

| | | | |
|---|---|---|---|
| O | 3.75455 | 5.16211 | 3.92455 |
| O | 1.66477 | 0.29903 | -0.66179 |
| O | -0.05798 | 1.50771 | -3.18661 |
| O | -1.92006 | -1.96178 | -2.80096 |
| O | -3.89482 | -1.95334 | -1.66987 |
| O | -1.19502 | -4.33335 | -1.26061 |
| O | -0.79650 | -5.15151 | -3.34506 |
| O | 2.16220 | -4.70786 | -1.38360 |
| O | 1.30964 | -6.80953 | -1.23912 |
| O | 1.49205 | -1.97298 | -0.49735 |
| O | -1.06279 | 0.45855 | -1.43436 |
| C | 5.10370 | 1.04030 | 4.17433 |
| H | 5.78832 | 0.95913 | 3.32164 |
| H | 5.59884 | 0.59584 | 5.04585 |
| H | 4.93541 | 2.09871 | 4.38277 |
| O | 0.91182 | 1.67374 | 1.00368 |
| O | 1.28422 | 2.39161 | 2.21875 |

Coordinates for 1*R*,4*S*-**2f**

| | | | |
|---|---|---|---|
| C | 0.70728 | 3.22334 | 2.22893 |
| C | -0.17498 | 2.06709 | 2.72372 |
| C | -0.28195 | 1.14386 | 1.75582 |
| H | -0.80920 | 0.20252 | 1.84996 |
| C | 0.44570 | 1.45994 | 0.47160 |
| C | -0.43761 | 3.11158 | -1.33152 |
| H | -0.54409 | 2.33173 | -2.07815 |
| C | -0.75466 | 4.44513 | -1.62028 |
| H | -1.12265 | 4.70780 | -2.60820 |
| C | -0.59989 | 5.43512 | -0.64994 |
| H | -0.84723 | 6.46654 | -0.88636 |
| C | -0.12854 | 5.11507 | 0.63086 |
| H | 0.00280 | 5.88868 | 1.37908 |
| C | 0.17075 | 3.78984 | 0.92229 |
| C | 0.01249 | 2.80158 | -0.05690 |
| C | 2.12589 | 4.94698 | 3.15432 |
| H | 2.40393 | 5.15537 | 2.11144 |
| C | 1.90218 | 6.23477 | 3.95658 |
| H | 1.56546 | 5.96655 | 4.96010 |
| C | 3.20935 | 7.03316 | 4.01110 |
| H | 3.42936 | 7.45453 | 3.02591 |
| C | 4.38657 | 6.17511 | 4.48537 |
| H | 4.30556 | 5.98177 | 5.55781 |
| C | 4.43082 | 4.84455 | 3.71132 |
| H | 4.67494 | 5.04552 | 2.65589 |
| C | -0.22014 | 7.35026 | 4.05045 |
| C | -1.14910 | 8.21416 | 3.23414 |
| H | -1.93255 | 8.61264 | 3.87973 |
| H | -1.60961 | 7.60948 | 2.44442 |

| | | | |
|---|---|---|---|
| H | -0.59805 | 9.02617 | 2.75152 |
| C | 3.12615 | 9.39228 | 4.46723 |
| C | 2.92556 | 10.37296 | 5.59664 |
| H | 1.95215 | 10.20642 | 6.06933 |
| H | 2.98484 | 11.39055 | 5.20947 |
| H | 3.68879 | 10.22026 | 6.36671 |
| C | 6.50429 | 7.03394 | 5.23025 |
| C | 7.63851 | 7.93443 | 4.80732 |
| H | 7.25501 | 8.92664 | 4.54884 |
| H | 8.12302 | 7.53126 | 3.91191 |
| H | 8.36280 | 8.01086 | 5.61863 |
| C | 5.43579 | 3.84515 | 4.27214 |
| H | 6.43732 | 4.27988 | 4.29502 |
| H | 5.14378 | 3.52747 | 5.27517 |
| C | 4.86928 | 1.56939 | 3.80911 |
| C | 4.85572 | 0.55131 | 2.69624 |
| H | 4.72011 | -0.44436 | 3.12268 |
| H | 5.77754 | 0.59634 | 2.11038 |
| H | 4.01424 | 0.77455 | 2.03020 |
| C | -0.73799 | 1.94926 | 4.13801 |
| C | 0.42400 | 1.84160 | 5.15854 |
| H | 1.07785 | 2.71383 | 5.13327 |
| H | 1.04137 | 0.95949 | 4.95774 |
| H | 0.01196 | 1.74680 | 6.17061 |
| C | -1.60092 | 0.67747 | 4.26011 |
| H | -1.01524 | -0.23143 | 4.08308 |
| H | -2.44446 | 0.68837 | 3.55945 |
| H | -2.01081 | 0.61225 | 5.27364 |
| C | 0.90995 | -0.78523 | -0.22614 |
| H | 0.98950 | -0.93196 | 0.86112 |

| | | | |
|---|---|---|---|
| C | -0.03626 | -1.82064 | -0.84481 |
| H | -0.21052 | -1.56279 | -1.89180 |
| C | 0.54935 | -3.22858 | -0.72258 |
| H | 0.51194 | -3.56802 | 0.31687 |
| C | 1.99079 | -3.25559 | -1.23547 |
| H | 2.00379 | -3.11751 | -2.31775 |
| C | 2.81138 | -2.14538 | -0.55710 |
| H | 2.83391 | -2.32619 | 0.53176 |
| C | -2.40936 | -1.45490 | -0.83294 |
| C | -3.60073 | -1.41802 | 0.09192 |
| H | -3.50409 | -0.57372 | 0.78372 |
| H | -3.64846 | -2.33135 | 0.69230 |
| H | -4.51172 | -1.30126 | -0.49590 |
| C | -0.82927 | -5.18802 | -0.90807 |
| C | -1.58142 | -6.01004 | -1.92500 |
| H | -2.06083 | -6.85452 | -1.42909 |
| H | -0.88969 | -6.36829 | -2.69460 |
| H | -2.33218 | -5.39148 | -2.42721 |
| C | 2.88332 | -5.37067 | -1.93955 |
| C | 3.62869 | -6.57560 | -1.43070 |
| H | 3.27729 | -6.87458 | -0.44050 |
| H | 4.68844 | -6.30462 | -1.35706 |
| H | 3.52528 | -7.39544 | -2.14321 |
| C | 4.25953 | -2.07596 | -1.02139 |
| H | 4.69747 | -1.13902 | -0.66667 |
| H | 4.82386 | -2.92281 | -0.62873 |
| C | 5.08864 | -3.05535 | -3.03819 |
| C | 4.98835 | -2.98389 | -4.53923 |
| H | 4.84683 | -1.95708 | -4.88304 |
| H | 4.11819 | -3.57713 | -4.84386 |

| | | | |
|---|---|---|---|
| H | 5.88229 | -3.42357 | -4.98496 |
| O | 0.95684 | 4.17483 | 3.22306 |
| O | 0.90604 | 7.05611 | 3.32928 |
| O | -0.42675 | 6.95485 | 5.17238 |
| O | 3.04084 | 8.11505 | 4.94170 |
| O | 3.33073 | 9.67623 | 3.31083 |
| O | 5.58140 | 6.93759 | 4.22944 |
| O | 6.40931 | 6.46212 | 6.29101 |
| O | 5.49374 | 2.71172 | 3.39662 |
| O | 4.39684 | 1.41384 | 4.91037 |
| O | 3.15197 | 4.22720 | 3.80621 |
| O | 0.40662 | 0.49139 | -0.52203 |
| O | -2.42646 | -1.23073 | -2.01862 |
| O | -0.24508 | -4.11779 | -1.52126 |
| O | -0.74129 | -5.42564 | 0.27370 |
| O | 2.58690 | -4.52163 | -0.90984 |
| O | 2.61048 | -5.14600 | -3.09677 |
| O | 4.34202 | -2.07631 | -2.45669 |
| O | 5.71403 | -3.88773 | -2.41701 |
| O | 2.18691 | -0.89069 | -0.82204 |
| O | -1.28036 | -1.77772 | -0.12945 |
| C | -1.63332 | 3.16823 | 4.47117 |
| H | -1.07278 | 4.10457 | 4.47809 |
| H | -2.07418 | 3.03541 | 5.46619 |
| H | -2.45573 | 3.25887 | 3.75091 |
| O | 1.88470 | 1.64033 | 0.81740 |
| O | 2.01640 | 2.59483 | 1.91847 |
